# Proteasome activity contributes to pro-survival response upon mild mitochondrial stress in *Caenorhabditis elegans*

**DOI:** 10.1371/journal.pbio.3001302

**Published:** 2021-07-12

**Authors:** Maria Sladowska, Michał Turek, Min-Ji Kim, Krzysztof Drabikowski, Ben Hur Marins Mussulini, Karthik Mohanraj, Remigiusz A. Serwa, Ulrike Topf, Agnieszka Chacinska

**Affiliations:** 1 ReMedy International Research Agenda Unit, University of Warsaw, Warsaw, Poland; 2 Centre of New Technologies, University of Warsaw, Warsaw, Poland; 3 Institute of Biochemistry and Biophysics, Polish Academy of Sciences, Warsaw, Poland; 4 IMol Polish Academy of Sciences, Warsaw, Poland; University of Newcastle upon Tyne, UNITED KINGDOM

## Abstract

Defects in mitochondrial function activate compensatory responses in the cell. Mitochondrial stress that is caused by unfolded proteins inside the organelle induces a transcriptional response (termed the “mitochondrial unfolded protein response” [UPRmt]) that is mediated by activating transcription factor associated with stress 1 (ATFS-1). The UPRmt increases mitochondrial protein quality control. Mitochondrial dysfunction frequently causes defects in the import of proteins, resulting in the accumulation of mitochondrial proteins outside the organelle. In yeast, cells respond to mistargeted mitochondrial proteins by increasing activity of the proteasome in the cytosol (termed the “unfolded protein response activated by mistargeting of proteins” [UPRam]). The presence and relevance of this response in higher eukaryotes is unclear. Here, we demonstrate that defects in mitochondrial protein import in *Caenorhabditis elegans* lead to proteasome activation and life span extension. Both proteasome activation and life span prolongation partially depend on ATFS-1, despite its lack of influence on proteasomal gene transcription. Importantly, life span prolongation depends on the fully assembled proteasome. Our data provide a link between mitochondrial dysfunction and proteasomal activity and demonstrate its direct relevance to mechanisms that promote longevity.

## Abstract 2

Mitochondrial import stress is a challenge for cellular protein homeostasis but at low level can be beneficial by activating the proteasome and increasing protein degradation. This study shows that activation of proteostasis compensatory mechanisms can lead to extension of lifespan in the nematode Caenorhabditis elegans.

## Introduction

The dual origin of genes that encode mitochondrial proteins constitutes a regulatory challenge for eukaryotes to maintain cellular and mitochondrial homeostasis. The vast majority of proteins that are destined for the mitochondrion are produced by cytosolic ribosomes and subsequently imported into the organelle. Complex import machinery facilitates translocation and sorting of precursor proteins into mitochondrial subcompartments [1–4]. Precursor proteins enter mitochondria through the translocase of the outer mitochondrial membrane (TOM40 complex) [5]. Proteins that are destined for the mitochondrial matrix further relay through the translocase of the inner mitochondrial membrane (TIM23 complex) in cooperation with the adenosine triphosphate (ATP)-driven presequence translocase-associated motor (PAM), which is localized on the matrix side of the inner membrane (IM) [6]. The functional components of mitochondrial import machinery are conserved in eukaryotes, but their function and interplay are best studied in yeast and mammalian cells. In *Caenorhabditis elegans*, genes that encode mitochondrial import components are not very well characterized, with a limited number of tools that are available to study precursor protein import in this multicellular organism [7].

Mitochondria play a crucial role in cellular energy metabolism, various biosynthetic pathways, and cellular signaling that is mediated by the release of reactive oxygen species and calcium [8–11]. Mitochondrial dysfunction can thus be detrimental to the function of cells and is linked to pathophysiology and aging in humans [12–14]. However, protective stress responses have evolved to counteract mitochondrial dysfunction. In the model organism, such responses can be elicited by inducing mild mitochondrial stress [15], which can also have beneficial effects for the life span and overall health. In *C*. *elegans*, the prevailing response to mitochondrial stress is the mitochondrial unfolded protein response (UPRmt) [16–19]. Defects inside the organelle such as low levels of mitochondrial quality control proteins, the inhibition of mitochondrial protein synthesis, the dysregulation of oxidative phosphorylation (OXPHOS), and high levels of reactive oxygen species are potent inducers of the UPRmt [20–24]. The UPRmt activation initiates a transcriptional program that increases the production of mitochondrial chaperones and proteases and stimulates metabolic pathways [25,26]. In *C*. *elegans*, the activating transcription factor associated with stress 1 (ATFS-1) largely mediates the UPRmt. ATFS-1 has a bipartite sorting signal, whereby it localizes to the mitochondrial matrix under basal conditions but accumulates in the nucleus during mitochondrial stress [25,27]. Therefore, the blockade of ATFS-1 translocation to mitochondria upon defects in mitochondrial import also activates the UPRmt [28]. The loss of ATFS-1 abolishes activation of UPRmt. Although activation of the UPRmt has been linked to life span extension, the transcriptional function of ATFS-1 alone is insufficient for life prolongation [29]. The UPRmt is a stress response that is also present in mammalian cells, albeit regulated in a distinct manner [30]. The UPRmt has not yet been described in yeast.

Many efforts focus on understanding responses that restore mitochondrial function, but much less attention has been given to cellular consequences of mitochondrial stress. Given that mitochondrial protein homeostasis is associated with cellular proteostasis, there must be mechanisms that modulate cellular protein homeostasis upon mitochondrial dysfunction [31–33]. When mitochondrial precursor proteins cannot enter the organelle, they subsequently accumulate in the cytosol, causing a burden for cytosolic protein homeostasis. We and others recently discovered that the slowdown of mitochondrial import in yeast induces a posttranscriptional response, leading to the modulation of protein translation and protein degradation [34,35]. The response was termed the “unfolded protein response activated by mistargeting of proteins” (UPRam). Mitochondrial proteins can be degraded by the proteasome, the major protein degradation machinery in the cytosol [36], and proteasomal activity increases upon mitochondrial import stress [34]. The proteasome complex consists of a 20S core particle and two 19S regulatory cap subunits. Together, these subunits form the 26S proteasome complex, which is dedicated mostly to the degradation of ubiquitinated protein targets [37,38]. Mitochondrial import stress in yeast enhances proteasomal activity by increasing assembly of the proteasomal complex rather than increasing the production of new single subunits [34]. Proteotoxic stress can activate the proteasome in mammalian cells [39,40]. Moreover, the overexpression of a proteasome subunit could also increase proteasomal activity, and this was beneficial for the life span of *C*. *elegans* [41]. However, the UPRam has not been identified in higher eukaryotes. In the present study, we explored stress responses that were activated upon impairments in mitochondrial protein import machinery in *C*. *elegans*. Similar to our previous observations in yeast [34], we found that interfering with protein import into mitochondria activates the cytosolic protein degradation machinery. We also found that the activation of this response led to life span extension. Interestingly, this increase in longevity depended on both, the fully functional proteasome and ATFS-1, the central mediator of the UPRmt, thereby demonstrating a cross-talk between the UPRam and UPRmt pathways.

## Results

### Knockdown of *dnj-21* results in mild mitochondrial stress

**Fig 1:**
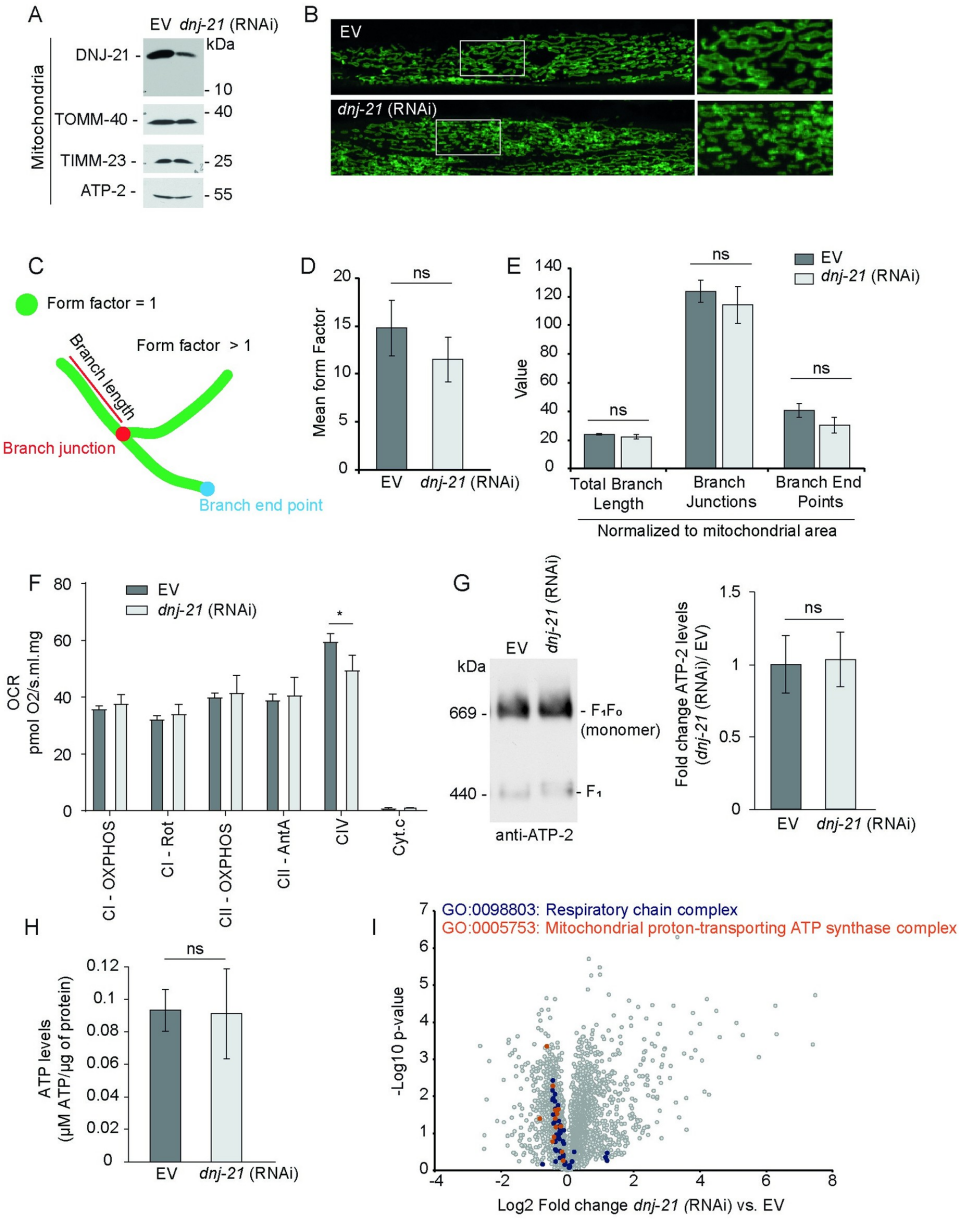
Depletion of mitochondrial import component results in mild mitochondrial stress. (A, F, G) Mitochondria were isolated from wild-type worms that were grown on RNAi bacteria from the embryonic stage to young adulthood. (A) Protein content was analyzed using SDS-PAGE and western blot in at least 2 biological replicates. (B) Worms were cultured on plates until they developed into young adults. Confocal images of *C*. *elegans* that expressed GFP targeted to the mitochondrial outer membrane in the body wall muscle (ACH89) are shown. A magnification of the white rectangle is shown in the right panel. (C) Schema of features that were analyzed to quantify mitochondrial morphology. Depicted features are according to the applied analysis tool “Mitoanalyzer.” Green shows possible shapes of mitochondria (green circle, fragmented mitochondria; elongated lines, mitochondrial network). (D, E) Images of at least 2 ROIs from 10 images per condition were analyzed to quantify mitochondrial morphology. (D) Quantification of the shape of mitochondria, expressed as the mean of the “form factor,” in which a value of 1 corresponds to defragmented mitochondria. The data are presented as mean ± SEM. (E) Quantification of mitochondrial morphology for the features as indicated. Values were normalized to the mitochondrial area in the corresponding ROI. The data are presented as mean ± SEM. (F) The OCR that depended on the activity of respiratory chain complex I (CI), CII, and CIV was analyzed from isolated mitochondria. The data are presented as mean ± SD (*n* = 4). *p < 0.05. (G) Solubilized mitochondria were subjected to BN-PAGE and analyzed by western blot (left panel). The levels of respiratory chain complex V and its homodimer, V2, were quantified by the densitometry analysis of ATP-2 levels from the western blot (right panel). The data are presented as mean fold change ± SEM (*n* = 5). (H) Synchronized worms that were grown on RNAi bacteria to young adulthood were harvested, and total cellular ATP levels were measured in the whole worm lysate. The data are expressed as mean ± SD (*n* = 3). ns, not significant. (I) Wild-type worms that were depleted of DNJ-21 or an EV control were subjected to proteomics analysis. The distribution of fold change in protein levels filtered by specific GO terms is shown (red and blue circles). Proteomics data are also presented in S1 Table and PXD023830. Underlying numerical data are presented in S1 Data. AntA, antimycin A; ATP, adenosine triphosphate; BN-PAGE, blue native polyacrylamide gel electrophoresis; EV, empty vector; GFP, green fluorescent protein; GO, Gene Ontology; ns, not significant; OCR, oxygen consumption rate; OXPHOS, oxidative phosphorylation; RNAi, RNA interference; ROI, region of interest; Rot, rotenone.

Dysfunction of the motor subunit of TIM23 translocase, the PAM complex that is located on the matrix side of the mitochondrial IM in *Saccharomyces cerevisiae*, was shown to decrease mitochondrial protein import and precursor accumulation in the cytosol [34]. We used an analogous model to study the nonmitochondrial response to defective mitochondrial import machinery in *C*. *elegans* (Fig 1).

DNJ-21 is the functional homolog of yeast Pam18. The knockdown of *dnj-21* by RNA interference (RNAi) decreased DNJ-21 protein levels in mitochondria, whereas protein levels of TIMM-23 and TOMM-40 remained unchanged. Furthermore, the subunit of the ATPase, ATP-2, remained largely unchanged (Fig 1A). Continuous exposure of the F0 generation to *dnj-21* RNAi allowed the worms to develop comparably to control worms, only exhibiting a mild brood size defect (S1A Fig). However, the development of F1 progeny was inhibited during embryogenesis or larval development (S1B Fig). Further experiments were conducted with worms that were exposed to RNAi conditions in the F0 generation. To investigate whether the depletion of DNJ-21 alters mitochondrial morphology, we visualized mitochondria by expressing green fluorescent protein (GFP) targeted to the outer mitochondrial membrane in worm body wall muscle cells. Mitochondrial morphology was quantitatively analyzed by assessing the overall shape of the mitochondrial network and determining mitochondrial branch length, junctions, and branch end points (Fig 1C). No significant differences in mitochondrial morphology were found between the empty vector (EV) control and *dnj-21* RNAi (Fig 1D and 1E). To characterize potential defects that were caused by *dnj-21* RNAi inside the organelle, we investigated the function of respiratory chain complexes. An equal concentration of isolated mitochondria was subjected to high-resolution respirometry analysis. The efficiency of DNJ-21 depletion and levels of other mitochondrial proteins were controlled by western blot analysis (S1C–S1E Fig). Mitochondria were intact, indicated by the absence of cytochrome C leakage from mitochondria (Fig 1F). The oxygen consumption rate (OCR) that depended on the activity of respiratory chain complex I (CI), CII, and CIV was determined. Although a significant but small decrease in OCR was observed upon DNJ-21 depletion in CIV, we did not detect other differences in the OCR compared with control conditions (Fig 1F). The functional analysis was complemented by investigations of the integrity of the ATPase complex in isolated mitochondria using blue native polyacrylamide gel electrophoresis (BN-PAGE). No changes in ATP-2 protein levels or ATPase complex assembly were detected (Fig 1G). Moreover, cellular ATP levels in total worm extracts were unchanged upon the knockdown of *dnj-21* (Fig 1H). To achieve broader insights into potential changes that occurred upon DNJ-21 depletion, we performed a quantitative, label-free proteomics analysis (S1 Table). We compared protein levels upon the knockdown of *dnj-21* with control conditions (EV). Worms were grown until the young adult stage, and total worm extracts were subjected to mass spectrometry (MS) (S1F Fig). Our analysis quantified 4,035 proteins (S1 Table). We analyzed the quantified proteins according to Gene Ontology (GO) term. Consistent with our previous findings in yeast [34], proteins that are involved in mitochondrial translation remained mostly not statistically significantly changed (S1G Fig), but protein levels of cytosolic translation machinery decreased (S1H Fig). We more closely examined proteins of respiratory chain complexes and the ATP synthase complex in our dataset and found that these protein levels tended to decrease upon DNJ-21 depletion (Fig 1I). Thus, the knockdown of *dnj-21* caused mild mitochondrial stress, but the changes in mitochondrial protein levels did not change mitochondrial appearance or ATP levels in the cell, which could negatively impact the function of other energy-demanding cellular processes.

### Impairments in mitochondrial import machinery can lead to life prolongation

**Fig 2:**
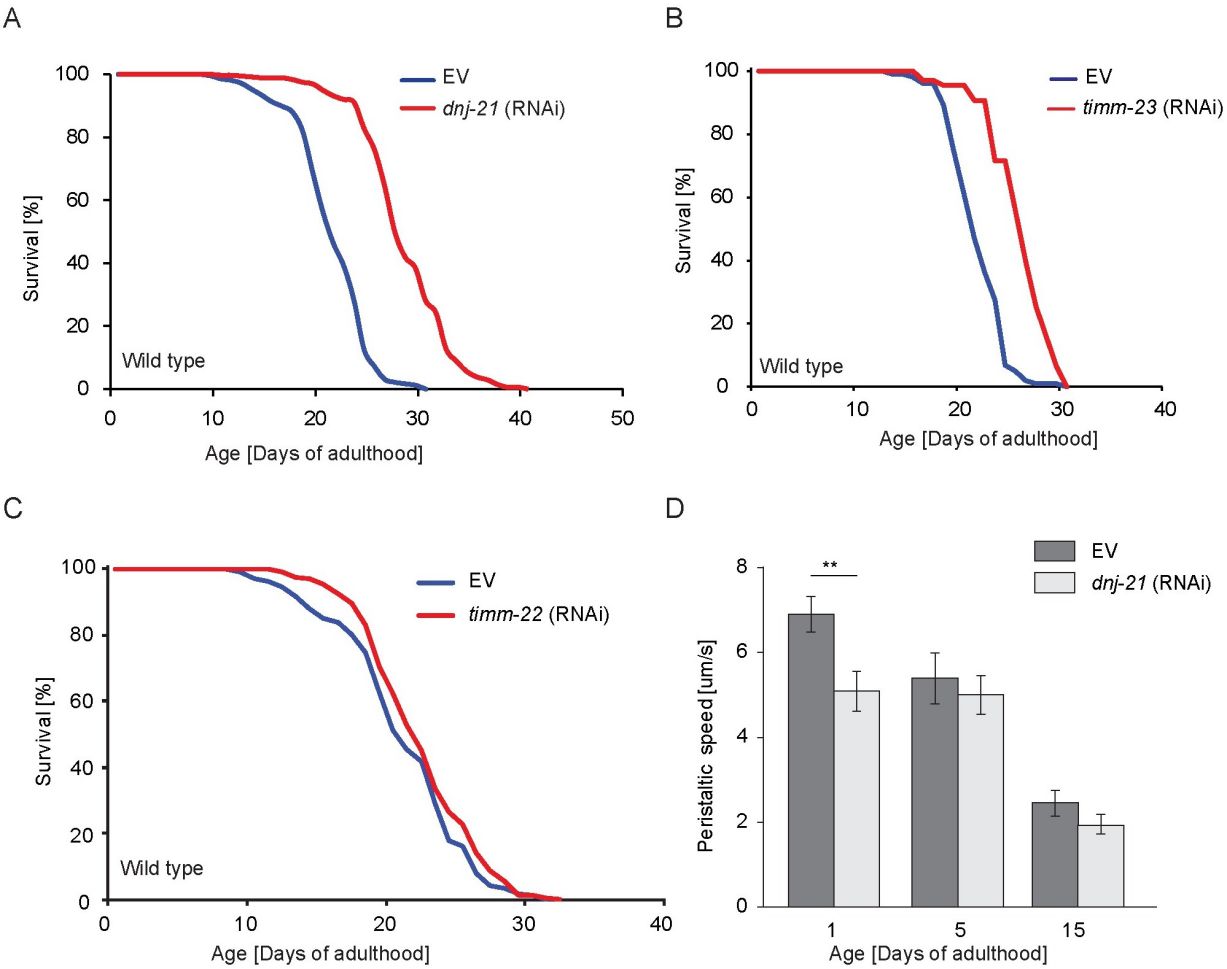
Depletion of mitochondrial import components correlates with life span prolongation. (A–D) Wild-type worms were kept on RNAi plates throughout the experiment. (A–C) Survival curves upon the depletion of mitochondrial import machinery components. Life span curves represent combined data from independent experiments. Life span values are shown in S2 Table. (D) The worm population was assayed for peristaltic speed on the indicated days, starting on the first day of the reproductive phase. Mean ± SEM, *n* = 12 to 18 (*n* indicates the number of worms; 2 biological replicates were performed for each condition). **p < 0.01. Underlying numerical data are presented in S1 Data. EV, empty vector; RNAi, RNA interference.

*C*. *elegans* grown at 20 °C that were treated throughout their life with RNAi that targeted *dnj-21* exhibited a significant increase in median and maximum life span compared with worms that were treated with the control condition (Fig 2A and S2 Table).

The knockdown of *timm-23* by RNAi resulted in lower levels of TIMM-23 protein (S2A Fig). Similar to *dnj-21* knockdown, *timm-23* silencing led to life prolongation (Fig 2B and S2 Table). Extension of the maximum life span upon *timm-23* knockdown was not as pronounced as *dnj-21* knockdown, likely because of a stronger impact on mitochondrial function (S2B Fig). We also investigated whether disrupting other major import pathways activated a beneficial response to result in life span extension. Mitochondrial intermembrane space import and assembly protein 40 (Mia40) in yeast and human cells controls the import of precursor proteins into the IM space [42,43]. The MIA40 pathway has not been characterized in *C*. *elegans*, but we found 4 potential homologs of Mia40, 2 of which seemingly arise from gene duplication (S2C and S2D Fig). The knockdown of 3 of the potential Mia40 homologs (ZK616.2, ZK616.3, and F11C1.1) led to life prolongation (S2E and S2F Fig and S2 Table). In contrast, knockdown of the homolog of human *timm22*, C47G2.3, had a minimal impact on life span (Fig 2C and S2B Fig and S2 Table). Altogether, generally, alterations of mitochondrial protein import resulted in life span extension.

We tested whether life prolongation upon *dnj-21* knockdown also resulted in an extension of health span (i.e., the period of life spent in good health and free from disabilities of aging). In *C*. *elegans*, movement of the worm declines during aging, and measurements of movements are used to assess the worm’s health span [44]. Compared with the control conditions, overall crawling speed and body movement up to day 15 of adulthood were unaltered after DNJ-21 depletion or rather motility of animals was slightly decreased at day 1 of adulthood (Fig 2D and S2G and S2H Fig). Hence, a slight deficiency in mitochondrial protein import competence activated a beneficial response that led to the extension of life span but was insufficient to extend the health span.

### Knockdown of *dnj-21* mildly affects unfolded protein responses

**Fig 3:**
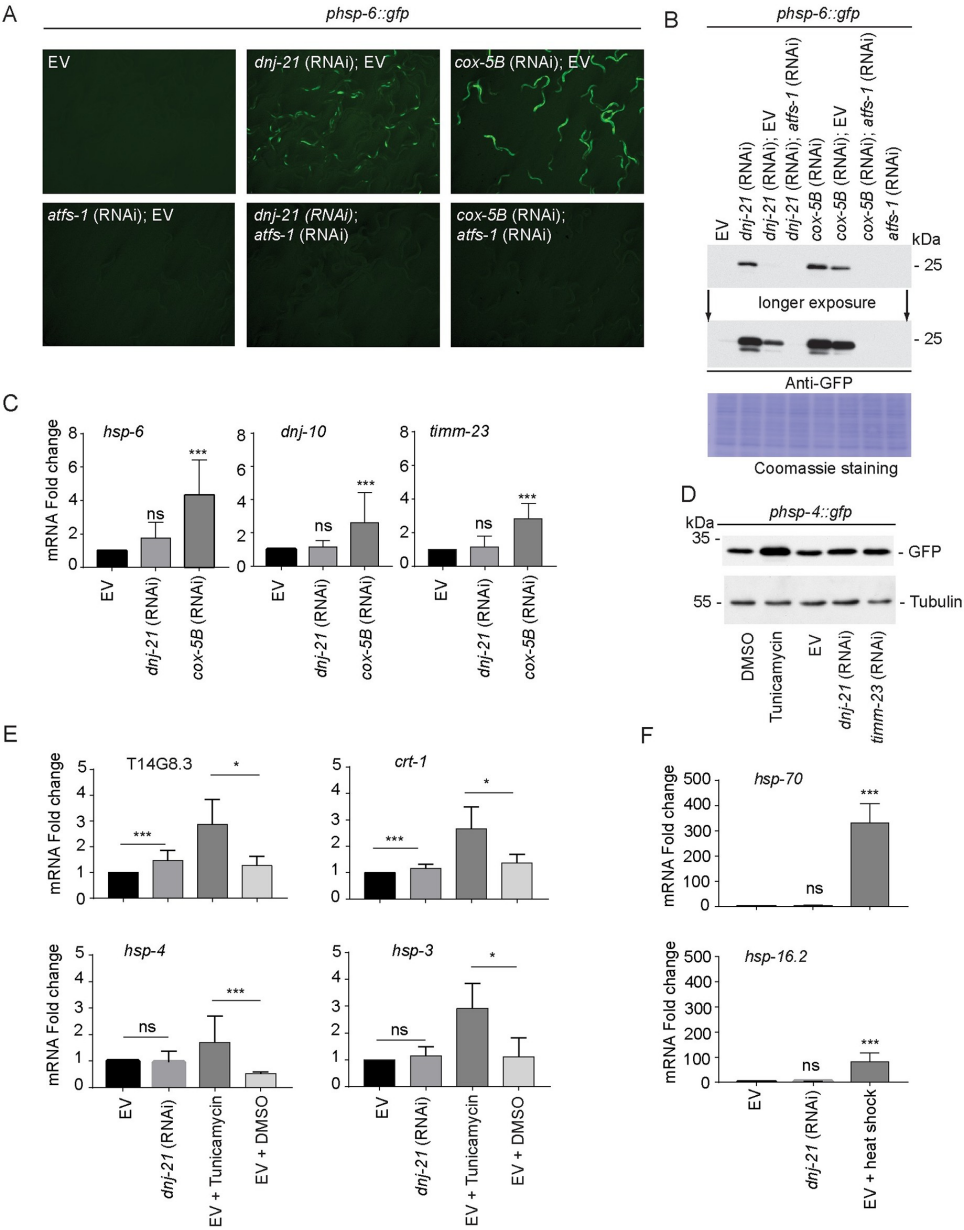
Depletion of DNJ-21 activates some features of transcriptional unfolded protein responses. (A, B) Worms that expressed *hsp-6p*::*gfp*, a transcriptional reporter for activation of the UPRmt, were kept starting at the embryonic stage on RNAi plates throughout the experiment. (A) Representative images of worms on the plate upon depletion of the indicated genes. Images were taken at the same magnification and exposure time when worms reached young adulthood. Microscopy analysis was repeated in 2 biological replicates. (B) Worms were collected when they reached young adulthood. Total worm lysate was separated by SDS-PAGE and analyzed by western blot with an antibody against GFP. Equal loading of proteins was assured by Coomassie staining. (C, E, F) RT-qPCR in wild-type worms that were kept on *dnj-21* RNAi or an EV control from the embryonic stage until young adulthood. The mRNA levels are presented as fold changes relative to the respective EV control (mean ± SD). qPCR analysis was repeated in 3 biological replicates. ***p < 0.005. (C) Expression of the selected target mRNAs that were activated during the UPRmt. Mann–Whitney *U* test was used for statistical analysis. (D) Worms that expressed *hsp-4p*::*gfp*, a transcriptional reporter for activation of the UPR_ER_, were collected when they reached young adulthood. As a positive control, worms were treated with tunicamycin or an equal volume of solvent (DMSO). Total worm lysate was separated by SDS-PAGE and analyzed by western blot against specific antibodies. Western blot analysis was repeated in 2 biological replicates. (E) Expression of selected target mRNAs that were activated during the unfolded protein response (UPR_ER_). As a positive control, worms were treated with tunicamycin or an equal volume of solvent (DMSO). Mann–Whitney *U* test was used for statistical analysis. (F) Expression of the selected target mRNAs that were activated during the HSR. As a positive control, wild-type worms were heat shocked for 15 min at 33 °C. Kruskal–Wallis test was used for statistical analysis. Underlying numerical data are presented in S1 Data. DMSO, dimethylsulfoxide; EV, empty vector; GFP, green fluorescent protein; HSR, heat shock response; ns, not significant; RNAi, RNA interference; RT-qPCR, quantitative real-time PCR; UPR_ER_, endoplasmic reticulum unfolded protein response; UPRmt, mitochondrial unfolded protein response.

Mitochondrial dysfunction frequently activates the UPRmt, which leads to stimulation of the transcriptional response to restore mitochondrial protein homeostasis [17–19,45,46]. UPRmt activation was previously correlated with life prolongation in *C*. *elegans* [17,22,25,46]. To determine whether the knockdown of *dnj-21* activates the UPRmt, we first used a *C*. *elegans* transcriptional reporter strain that expresses GFP under the *hsp-6* promoter (*phsp-6*::*gfp*). This promoter controls the expression of a mitochondrial chaperone of the heat shock protein 70 (Hsp70) family in worms and is one of the classic targets that is activated by ATFS-1, the main transcription factor that mediates the UPRmt [25]. The expression of GFP was observed by fluorescence microscopy upon *dnj-21* RNAi knockdown, suggesting that the UPRmt was activated (Fig 3A, upper panel), although to a lesser extent than following the knockdown of *cox-5B* (also called *cco-1*), a subunit of cytochrome c oxidase and bona fide activator of the *hsp-6* promoter (Fig 3A, top panel).

Moreover, the expression of *hsp-6* promoter-driven GFP, which is strongest in the worm intestine, was not as uniform upon DNJ-21 depletion compared with the bona fide UPRmt activators *cox-5B*, *spg-7*, and *mrps-5* (S3A Fig). The expression of GFP from the *hsp-6* promoter was abolished when ATFS-1 was depleted, as expected for a pathway that is mediated by ATFS-1 (Fig 3A, bottom panel). ATFS-1-dependent activation of the UPRmt upon *dnj-21* RNAi knockdown was consistent with previous findings that impairments in mitochondrial import drive ATFS-1 localization toward the nucleus [18,25,28]. The dilution of RNAi bacteria that targeted *dnj-21* with bacteria that carried the EV control decreased overall activation of the UPRmt, determined by western blot against GFP expression under the *hsp-6* promoter. The equal loading of proteins was assured by Coomassie staining. No GFP fluorescence was detected when ATFS-1 was depleted (Fig 3B).

The transcript levels of *dnj-10* and *timm-23*, the targets of ATFS-1, were not changed as assessed by quantitative real-time polymerase chain reaction (RT-qPCR; Fig 3C). In contrast, the knockdown of the control *cox-5B* significantly activated all ATFS-1 target transcripts. The *hsp-6* mRNA levels upon *dnj-21* knockdown were variable between experiments (compare Fig 3C and S8D Fig); however, the changes in mRNA levels were less pronounced compared to the positive control, knockdown of *cox-5B*. Additionally, we tested genes that code for metabolic enzymes and were previously reported to be increased upon UPRmt activation (S3C Fig; [47]). The tested genes (*gpd-2*, *aldo-1*, and *acs-2*) were increased upon *cox-5B* RNAi and also upon *dnj-21* RNAi, albeit to a lesser extent. Our data were supported by the proteomics analysis upon *dnj-21* RNAi (S1 Table). Only some canonical targets of ATFS-1, such as *timm-17B*.*1*, *glna-1*, and *aldo-1*, showed an increase in their protein levels [S3 Table; assuming significance when log2 fold change (*dnj-21* RNAi/EV) > 0.58 and *p*-value < 0.05], suggesting that *dnj-21* knockdown did not robustly activate the UPRmt. Next, we investigated whether other stress-inducible transcriptional responses were activated upon DNJ-21 depletion. We used a reporter strain that expressed GFP driven by the *hsp-4* promoter. HSP-4 belongs to the HSP70 family and is activated upon the endoplasmic reticulum unfolded protein response (UPR_ER_) [48]. Exposure to tunicamycin stimulates activation of the UPR_ER_. We observed an increase in *hsp-4*-driven GFP protein levels upon tunicamycin treatment compared with solvent control (dimethylsulfoxide [DMSO]) by western blot analysis. The knockdown of *dnj-21* or *timm-23* resulted in a mild increase in GFP levels compared with EV control (Fig 3D). The western blot results were consistent with fluorescent images of worms that expressed *phsp-4*::*gfp*, showing the strongest activation of GFP expression upon tunicamycin treatment (S3B Fig). We analyzed established transcriptional targets of UPR_ER_ activation by quantitative real time PCR [48,49]. Tunicamycin treatment resulted in a significant increase in all of the analyzed transcript levels (Fig 3E). The levels of *hsp-4* did not increase upon *dnj-21* RNAi knockdown, which was consistent with the analysis of the GFP reporter strain. The mRNA levels of T14G8.3 and *crt-1* upon *dnj-21* RNAi knockdown did not increase beyond the solvent control of tunicamycin treatment (Fig 3E).

Finally, we investigated the possibility that *dnj-21* knockdown activates the heat shock response (HSR; or cytosolic unfolded protein response). The transcript levels of heat stress–inducible chaperones (*hsp-16*.*2* and *hsp-70*) were highly activated upon mild heat shock, but DNJ-21 depletion itself failed to activate their expression (Fig 3F). Our analysis of the activation of transcriptional reporters and mRNA levels of established targets of the different unfolded protein responses was in agreement with our proteomics data upon *dnj-21* knockdown. Some proteins annotated with the GO terms mitochondrial unfolded protein response, endoplasmic reticulum unfolded protein response, and response to heat showed only a tendency toward an increase upon DNJ-21 depletion compared with the EV control (S3D and S3E Fig). This indicates that DNJ-21 depletion did not efficiently activate the canonical transcriptional stress responses.

### Depletion of DNJ-21 activates the proteasome

**Fig 4:**
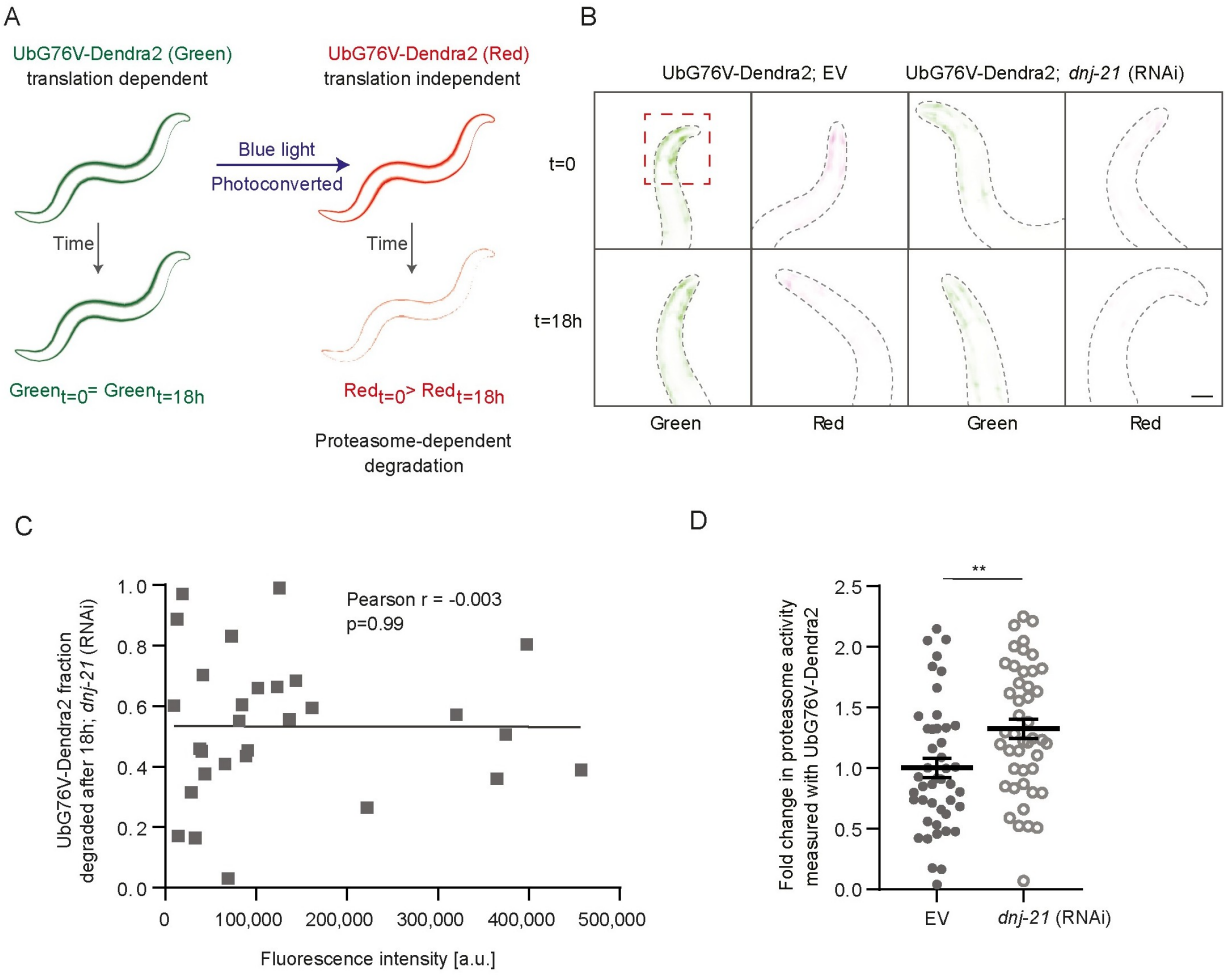
Mild mitochondrial stress regulates proteasomal activity. (A) Schema for measuring proteasomal activity using UbG76V-Dendra2 worms. Worms that expressed the photoconvertible GFP variant were exposed to blue light, thereby generating a red fluorescent variant. The time-dependent loss of red fluorescence reflects the speed of degradation by the proteasome. (B) Synchronized worms that expressed UbG76V-Dendra2 under the muscle-specific promoter (*unc-54*p) were exposed to RNAi until young adulthood. Green Dendra2 fluorescent protein was photoconverted, and the intensity of the red florescent variant was measured immediately and 18 h later. Representative images of 3 independent biological replicates are shown. Scale bar = 20 μm. (C) Proteasome activity (shown as UbG76V-Dendra2 fraction that degraded after 18 h upon *dnj-21* RNAi) does not correlate with expression of the reporter (shown as its fluorescence intensity). The gray line is the linear regression line, and each gray point represents one worm. *n* = 29. (D) Quantification of proteasomal activity upon DNJ-21 depletion. Shown are the ratios of t = 18 h/t = 0 that were normalized to the EV control. Data are presented as mean ± SEM; **p < 0.01, *n* = 45, where *n* indicates the number of worms; 3 biological replicates were measured for each condition. Underlying numerical data are presented in S1 Data. EV, empty vector; GFP, green fluorescent protein; RNAi, RNA interference.

In yeast, stimulation of the UPRam resulted in activation of the proteasome [34]. Therefore, we investigated whether *dnj-21* knockdown in worms elicited an equivalent response. To assess proteasomal activity, we used a *C*. *elegans* reporter strain that expressed photoconvertible GFP Dendra2 fused to a mutated version of ubiquitin (UbG76V). The fusion protein UbG76V-Dendra2 cannot be hydrolyzed and thus is polyubiquitinated and targeted to the proteasome. Exposure to blue light irreversibly photoconverted UbG76V-Dendra2 to a red fluorescent protein (Fig 4A) [50], a property that has been exploited to assess ubiquitin-dependent degradation by the proteasome in *C*. *elegans* [51–53].

Using our reporter strain, we compared the red fluorescent signal immediately after photoconversion and after 18 h (Fig 4B). The fraction of UbG76V-Dendra2 that degraded after 18 h did not correlate with fluorescent intensity of the reporter either upon *dnj-21* RNAi treatment (Fig 4C) or EV control (S4A Fig). This indicated that the readout of the assay was independent of initial expression of the reporter fluorophore. Compared with control worms, *dnj-21* knockdown resulted in a greater loss of red fluorescence, indicating an increase in the degradation of UbG76V-Dendra2 (Fig 4D). We observed an approximately 30% increase in proteasomal activity upon *dnj-21* knockdown. An increase of this magnitude was frequently observed in other studies that investigated the modulation of proteasomal activity [34,41,52]. No increase in proteasome activity was observed in a strain that only expressed Dendra2 without the ubiquitin modification (S4B Fig). Thus, the increase in proteasome activity upon DNJ-21 depletion may be specific for the function of the 26S proteasome, which works in a ubiquitin-dependent manner [54].

### Prolongation of life upon DNJ-21 depletion does not depend on ATFS-1 transcriptional function

**Fig 5:**
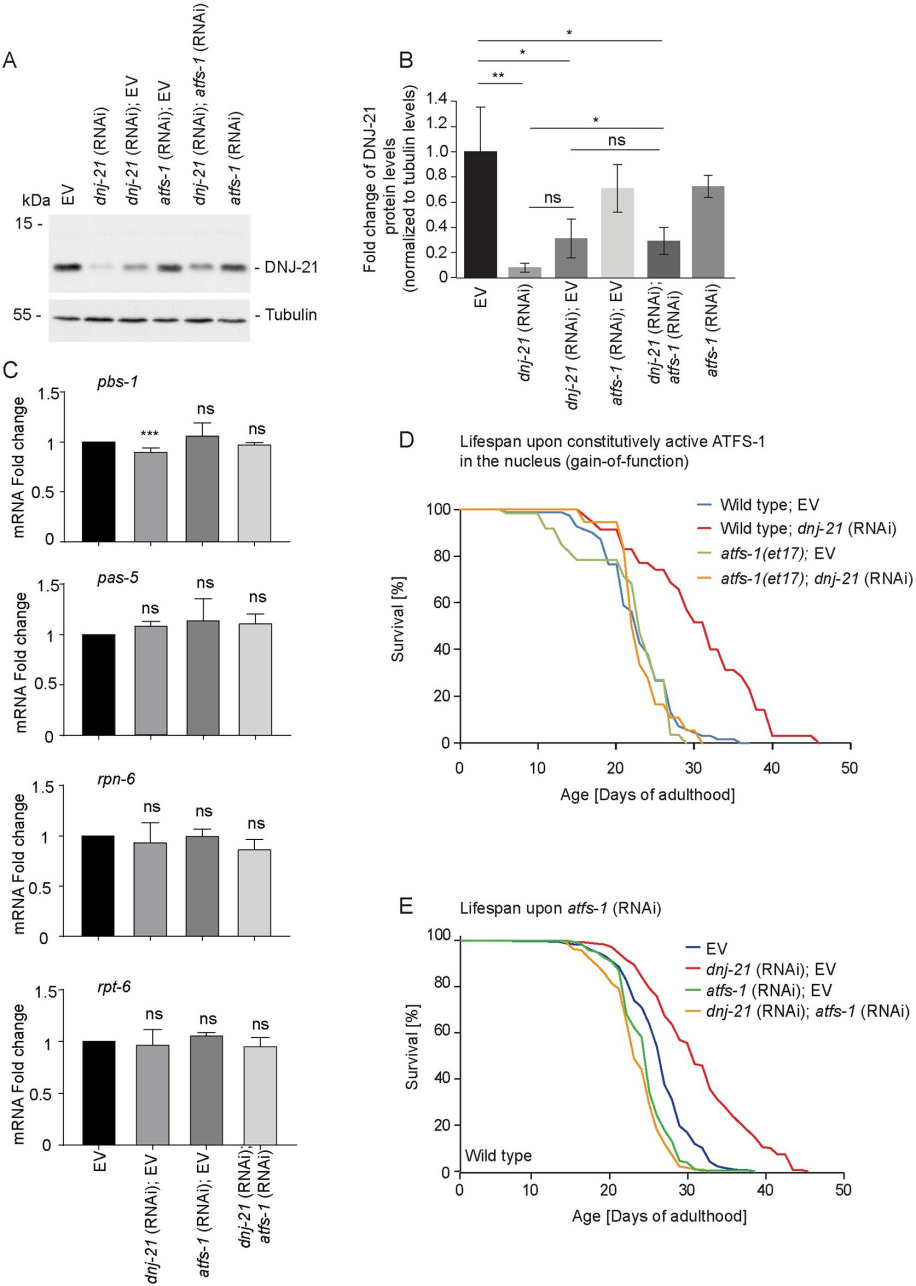
Life span extension upon DNJ-21 depletion does not depend on ATFS-1 nuclear function. (A) Wild-type worms were kept from L1 larvae until young adulthood on plates with RNAi that contained bacteria or an EV. Total worm extract was separated by SDS-PAGE and analyzed by western blot against specific antibodies. (B) Quantification of DNJ-21 protein expression levels that are shown in (A). Levels were normalized to tubulin expression levels. The data are expressed as mean ± SD. *n* = 3. *p < 0.05, **p < 0.01. (C) Wild-type worms were fed bacteria that contained the indicated RNAi from the L1 larval stage until young adulthood. RT-qPCR was performed to quantify levels of the indicated mRNAs. Mean ± SD. The qPCR analysis was repeated in 3 biological replicates. ***p < 0.005. Kruskal–Wallis test was used for statistical analysis. (D, E) Worms were fed RNAi that contained bacteria starting from the L1 larvae stage throughout the experiments. Survival curves of wild-type and mutant worms with silencing of the indicated genes. Life span values are presented in S2 Table. Underlying numerical data are presented in S1 Data. ATFS-1, activating transcription factor associated with stress 1; EV, empty vector; ns, not significant; RNAi, RNA interference; RT-qPCR, quantitative real-time PCR.

UPRmt activation that is mediated by ATFS-1 has been implicated in life span extension upon impairments in genes that are associated with OXPHOS, mitochondrial chaperones and proteases, and the inhibition of mitochondrial translation [21,22,46]. Although we did not observe the consistent activation of UPRmt targets upon *dnj-21* knockdown, we found the ATFS-1-dependent activation of at least *hsp-6* transcriptional reporter (Fig 3A and S3A Fig). This raised the issue of whether the life span extension upon DNJ-21 depletion depends on ATFS-1 function. We first assayed efficiency of the depletion of DNJ-21 protein levels under RNAi conditions that included diluting RNAi bacteria in a 1:1 ratio with either the EV control or bacteria for *atfs-1* RNAi (Fig 5A).

The depletion of DNJ-21 was less effective under double RNAi conditions compared with undiluted RNAi bacteria. The efficiency of *dnj-21* knockdown was comparable when bacteria were diluted with EV or *atfs-1* RNAi-mediating bacteria (Fig 5B). However, the dilution of bacteria that expressed *dnj-21*-targeting double stranded RNA (dsRNA) did not affect the increase in proteasome activation (S5A Fig) or life span extension (S5B Fig) compared with undiluted bacteria. Thus, DNJ-21 depletion-dependent proteasome activation was robust. The increase in proteasome activation by the overexpression of proteasome subunits was previously linked with life prolongation in *C*. *elegans* [41]. Activation of the UPRmt causes ATFS-1 to localize to the nucleus, activating a comprehensive transcriptional program that results in an increased expression of mitochondrial chaperones and proteases and the stimulation of OXPHOS and metabolic pathways [25,55]. However, no connection between ATFS-1 activity and proteasome regulation was found. Thus, we sought to determine whether ATFS-1 function as a transcription factor is necessary for the increase in proteasomal activity upon DNJ-21 depletion. We reanalyzed published transcriptomic data from *atfs-1* mutants [47]. We analyzed RNA sequencing data in *atfs-1* gain-of-function mutants [*atfs-1(et15)* and *atfs-1(et17)*] and an *atfs-1* deletion mutant [*atfs-1(gk3094)*] (S6A Fig). In the gain-of-function mutants, ATFS-1 localizes to the nucleus and constitutively activates a transcriptional program [29,56]. Interestingly, all quantified proteasome subunits in gain-of-function mutants decreased compared with wild-type worms, although not all significantly (S6B and S6C Fig; i.e., when assuming significance with a 2-fold change and *p*-value < 0.05). In contrast to the gain-of-function mutants, in the *atfs-1* deletion mutant, all of the quantified proteasomal subunits exhibited only a nonsignificant tendency toward an increase in expression (S6D Fig). Additionally, we analyzed expression levels of selected proteasome subunits by RT-qPCR based on *dnj-21* or *atfs-1* function. The knockdown of either gene or in combination with RNAi did not alter the expression of core subunits (*pbs-1* and *pas-5*) or regulatory subunits (*rpn-6*.*1* and *rpt-6*) of the proteasome (Fig 5C). The knockdown of bona fide activators of the UPRmt and ATFS-1 nuclear translocation (*cox-5B* and *mrps-5*) did not activate the proteasome analyzed using the UbG76V-Dendra2 strain (S5C Fig). Thus, our analysis excluded the possible ATFS-1-dependent transcriptional contribution to proteasome regulation under unstressed conditions and upon DNJ-21 depletion.

Finally, we analyzed the dependence of life span extension on *dnj-21* knockdown on ATFS-1 that was constitutively targeted to the nucleus (Fig 5D). We performed a life span assay in the *atfs-1(et17)* gain-of-function mutant [56] in combination with *dnj-21* RNAi. The *atfs-1(et17)* mutant alone did not exhibit life span extension, which is consistent with previous observations [29]. If the nuclear function of ATFS-1 contributes to DNJ-21 depletion-dependent life span prolongation, then life span extension should be unaffected in the *atfs-1(et17)* mutant background. However, we observed that *dnj-21* knockdown failed to extend life span in the *atfs-1* gain-of-function mutant (Fig 5D). This confirms that the nuclear function of ATFS-1 is negligible in response to DNJ-21 depletion but suggests that a nontranscriptional function of ATFS-1 outside the nucleus is required for DNJ-21 depletion–dependent life prolongation. This possibility was substantiated by the life span analysis that decreased *atfs-1* levels by RNAi (Fig 5E) or abolished ATFS-1 function (S5D Fig). In both cases, with the depletion of ATFS-1 and nonfunctional ATFS-1 protein, *dnj-21* knockdown failed to prolong life span.

### Involvement of ATFS-1 in the regulation of proteasomal activation

**Fig 6:**
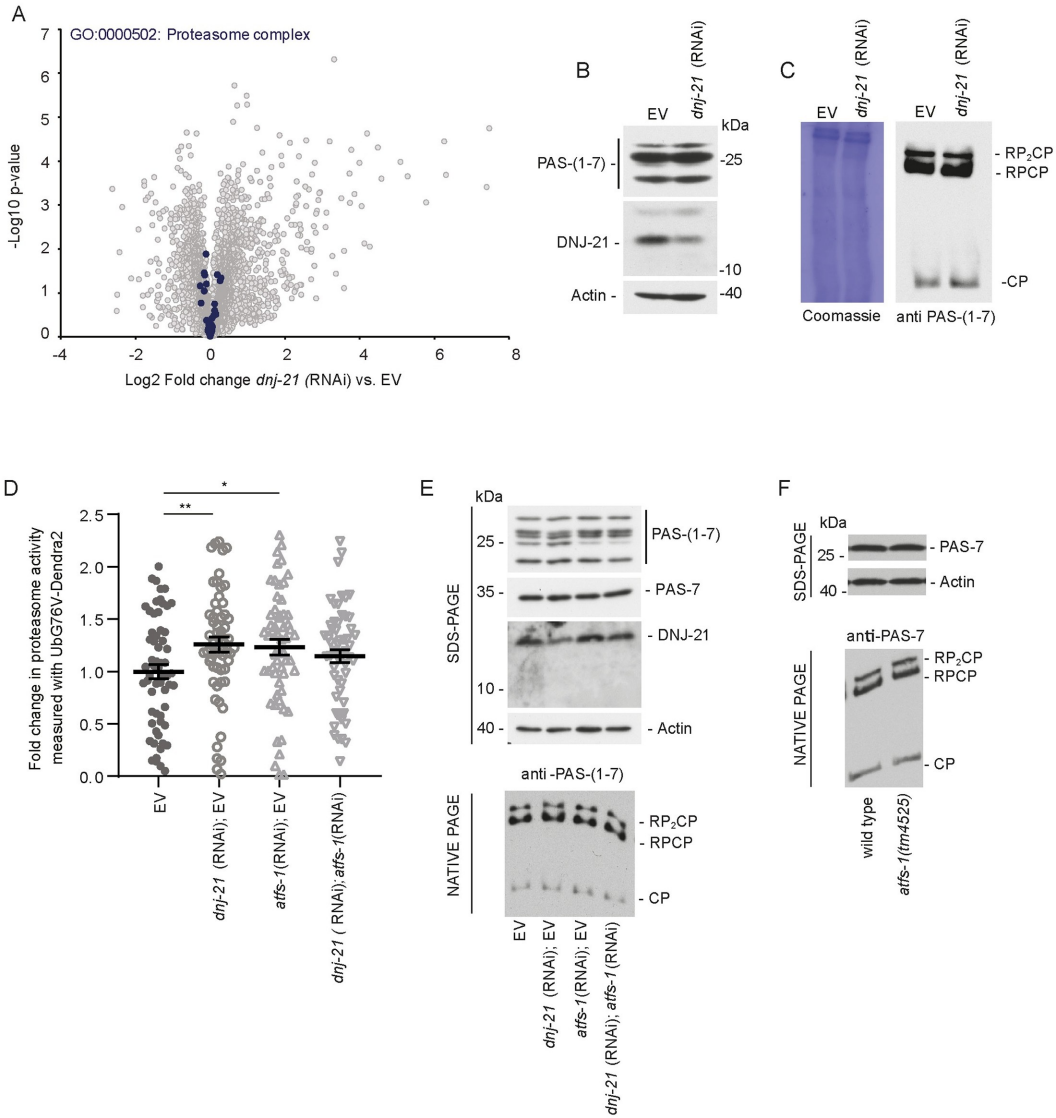
Proteasome activation upon DNJ-21 depletion partially depends on ATFS-1 function. (A) Distribution of fold change of protein levels filtered by specific GO terms (blue circles). Proteomics data are also presented in S1 Table and PXD023830. (B, C) Wild-type worms were grown on RNAi plates until young adulthood. (B) Total protein lysate was separated by SDS-PAGE and analyzed by western blot. (C) Protein extracts were separated using native PAGE and analyzed by western blot. Coomassie staining of the membrane is shown to demonstrate equal loading of the protein. Western blot analysis was repeated using at least 3 biological replicates. (D) Quantification of proteasomal activity upon the indicated RNAi treatment in worms that expressed UbG76V-Dendra2. Data are presented as mean ± SEM; *n* = 57–62 (*n* indicates the number of worms). Three biological replicates were measured for each condition. *p < 0.05, **p < 0.01. (E, F) Total protein extracts were separated by native PAGE or SDS-PAGE and analyzed by western blot using specific antibodies. (E) Synchronized worms were grown on agar plates that were seeded with RNAi bacteria. Western blot analysis was repeated in 3 biological replicates. (F) Synchronized worms were grown on plates that were seeded with HT115 bacteria. Western blot analysis was repeated in 2 biological replicates. Underlying numerical data are presented in S1 Data. ATFS-1, activating transcription factor associated with stress 1; EV, empty vector; GO, Gene Ontology; RNAi, RNA interference.

The activation of proteasome activity upon DNJ-21 depletion did not appear to have its origin in a transcriptional process (Fig 5C and 5D). Therefore, we investigated whether changes in the synthesis of proteasome subunits were the cause of the increase in proteasomal activity. All known proteasome subunits of the worm proteome were detected using our proteomics approach, but none of them showed a significant increase in protein abundance upon DNJ-21 depletion compared with the EV control (Fig 6A).

This was confirmed by the analysis of proteasome subunits using denaturing PAGE (Fig 6B). Using native PAGE, we assessed the composition of the proteasome complex using 2 different antibodies that detect alpha subunits of the proteasome core (PAS-7 and PAS-(1–7)). Upon *dnj-21* knockdown, we did not detect rearrangements of proteasome core particles or proteasome capped with the 19S regulatory subunit (Fig 6C and S7A Fig). We investigated whether a mild increase in proteasomal activity can change the abundance of ubiquitinated species in our model. A total protein extract of worms was probed with an anti-ubiquitin antibody, but *dnj-21* knockdown did not change the levels of ubiquitinated protein species compared with the control condition (S7B Fig). Thus, the mild impairment in mitochondrial import capacity led to an increase in proteasomal activity, possibly through an increase in assembly of the proteasome complex, similar to previous observations in yeast [34], demonstrating that the UPRam is a phenomenon that also exists in higher eukaryotes.

Next, we investigated the potential contribution of ATFS-1 to proteasome activation. Using worms that expressed UbG76V-Dendra2 to measure proteasomal activity (Fig 6D), we found that the dilution of RNAi bacteria that targeted *dnj-21* resulted in an increase in proteasomal activity, similar to undiluted bacteria (Fig 6D; see S5A Fig). Interestingly, the knockdown of *atfs-1* led to significant proteasome activation. Surprisingly, the simultaneous targeting of *dnj-21* and *atfs-1* had an antagonistic effect rather than a synergistic effect, causing the slight attenuation of proteasomal activity in response to *dnj-21* knockdown” alone (Fig 6D). Using fluorogenic peptides to assess proteasome activity in lysates of whole worms, we obtained results that were comparable to the fluorescent UbG76V-Dendra2 strain (S7D Fig). Although these changes were not significant, we observed a significant increase in proteasomal activity upon DNJ-21 depletion in a fraction that was enriched with mitochondria in the chymotrypsin activity assay (S7C Fig), suggesting spatial regulation of the proteasome. Similar to *dnj-21* knockdown, the activation of proteasomal activity upon *atfs-1* knockdown did not affect the amount of ubiquitinated protein species in a total worm extract (S7E Fig). Native PAGE analysis of the 26S proteasome from total worm extracts did not reveal any changes in the amount or arrangement of the proteasome complex when ATFS-1 was depleted (Fig 6E). We analyzed the proteasome complex in mutant worms that harbored a mutant allele of *atfs-1*, *atfs-1(tm4525)*, of which no functional ATFS-1 protein can be produced [25,29,55]. Using SDS-PAGE, we did not observe any changes in proteasome subunit abundance in *atfs-1(tm4525)* mutant compared with wild-type worms (Fig 6F, SDS-PAGE). Additionally, no changes in proteasome complex assembly were observed in *atfs-1(tm4525)* mutant in the native PAGE analysis or western blot analysis using antibodies against proteasomal core subunits (Fig 6F and S7F and S7G Fig). The *atfs-1* mutants did not show alterations in levels of ubiquitinated protein species (S7H Fig). Our data suggest that ATFS-1 depletion alone activated proteasomal activity similarly to DNJ-21 depletion. However, upon mild mitochondrial stress that was induced by *dnj-21* knockdown, a nontranscriptional function of ATFS-1 appears to be required to sufficiently increase proteasomal activity.

### Life span prolongation upon *dnj-21* knockdown requires a functional proteasome

**Fig 7:**
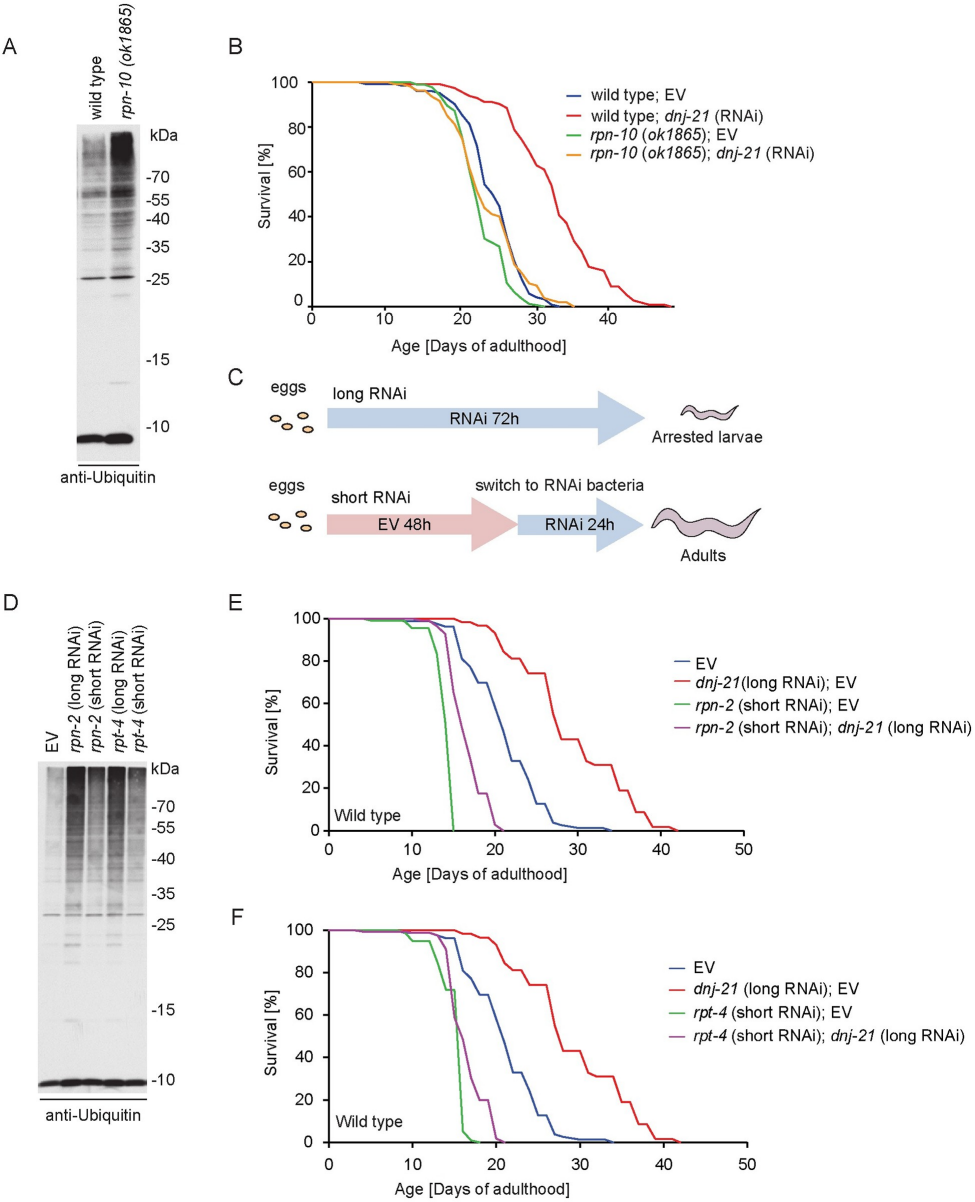
Life span prolongation depends on the proteasome. (A, D) Total worm lysates were separated by SDS-PAGE and analyzed by western blot using ubiquitin or specific antibody. Western blot analysis was repeated in 3 biological replicates. (B) Survival curve of wild-type worms and worms with the deletion of *rpn-10* that were treated with RNAi as indicated. Life span values are presented in S2 Table. (C) RNAi treatment schema for the depletion of *rpn-2* and *rpt-4*. The depletion of either target throughout development resulted in strong larval arrest, and the worms did not reach adulthood. Embryos were placed on plates with EV RNAi for the first 48 h of development and then switched to plates with RNAi for certain genes. This allowed the worms to develop to adulthood. For western blot analysis, worms were harvested after 24 h of treatment with RNAi. (E, F) Survival curve of wild-type worms that were treated with RNAi as indicated. Life span values are presented in S2 Table. Underlying numerical data are presented in S1 Data. EV, empty vector; RNAi, RNA interference.

To determine whether a functional proteasome is required for life span extension upon DNJ-21 depletion, we analyzed life span in the context of alterations that affect the efficient degradation of ubiquitinated protein species. The deletion of *rpn-10* is the only viable mutant of the proteasome in *C*. *elegans* [57–59]. RPN-10 is a non-ATPase subunit of the proteasome lid and serves as a ubiquitin receptor [57]. Deletion worms were superficially wild type and developed comparably to wild-type worms [59]. *Rpn-10* mutant worms expressed higher protein levels of proteasome core subunits (S8A Fig) and consequently had higher levels of the assembled proteasome core observed by native PAGE (S8B Fig). The deletion of *rpn-10* resulted in higher proteasomal activity, measured by fluorogenic peptides (S8C Fig), which likely is a consequence of higher levels of the 20S proteasome. However, ubiquitinated protein species accumulated in *rpn-10* deletion worms [57], indicating that higher proteasome abundance does not compensate for functional deficits in protein degradation (Fig 7A).

*rpn-10* deletion worms alone and upon *dnj-21* knockdown did not exhibit alterations of expression of the UPRmt targets *hsp-6* and *dnj-10* compared with control conditions (S8D Fig). This excludes the possibility that dysfunctional proteasomal degradation activates the UPRmt (S8D Fig). *rpn-10* deletion worms have a life span that is comparable to control conditions. The depletion of DNJ-21 in *rpn-10* deletion mutant worms abolished DNJ-21 depletion-dependent life span extension (Fig 7B and S2 Table).

To further elucidate the potential relationship between ATFS-1 and RPN-10 function in *dnj-21* knockdown–dependent life prolongation, we crossed the mutant *atfs-1(tm4525)* with the *rpn-10* deletion *rpn-10(ok1865)* (S8E Fig). The double mutant, ACH200 *atfs-1(tm4525);rpn-10(ok1865)*, had a shorter life span compared with *atfs-1* deletion alone and an even shorter life span compared with wild-type worms. The depletion of DNJ-21 in the *atfs-1* deletion mutant prevented life span extension. However, the additional deletion of *rpn-10* further shortened life span compared with DNJ-21 depletion in the absence of *atfs-1*. Thus, ATFS-1 and RPN-10 function are necessary for life span extension beyond the life span of wild-type worms.

We tested the effect of the knockdown of 2 additional subunits of the proteasome on DNJ-21 depletion–dependent life span prolongation. The depletion of RPN-2 (a component of the 19S regulatory particle base subcomplex) or RPT-4 (ATPase subunit of the 19S regulatory complex) throughout the worm’s life led to 100% larval arrest (see Fig 7C; treatment scheme referred to as “long RNAi”) because of severe impairments in proteasome function. To avoid detrimental effects on worm development, we adapted a “short RNAi” feeding protocol to achieve the moderate depletion of RPN-2 and RPT-4 (see Fig 7C; treatment scheme referred to as “short RNAi”). This allowed worms to reach adulthood. Representative bright-field images of worms that fed on *rpn-2* and *rpt-4* RNAi bacteria in the long or short treatment schema showed clear differences in developmental stage (S8F Fig). “Long RNAi” treatment resulted in a pronounced increase in ubiquitinated protein species, a hallmark of the proteasomal defect. “Short RNAi” treatment led to a milder proteasomal defect, but the accumulation of ubiquitinated species still increased compared with the control condition (Fig 7D). Although “short RNAi” treatment allowed worms to develop and exhibited only a mild defect in proteasome degradation capacity, the depletion of RPN-2 and RPT-4 resulted in a strong decrease in life span (Fig 7E and 7F). The concurrent knockdown of *rpn-2* and *dnj-21* (Fig 7E) or *rpt-4* and *dnj-21* (Fig 7F) resulted in the loss of DNJ-21 depletion–dependent life span extension. These results indicate that the functional 26S proteasome is necessary for a *dnj-21*-dependent beneficial effect on *C*. *elegans* life span.

## Discussion

Mild deficits in mitochondrial function have been linked to life span prolongation in *C*. *elegans* [45,46,60–62]. Importantly, the beneficial response was activated when stress was imposed during the early development of the worm. In early studies, compromised function of the respiratory chain resulted in low oxygen consumption, altered levels of reactive oxygen species, and generally reduced metabolism [63–66]. However, mechanisms that can explain the life span extension remained elusive. Independent of mitochondrial involvement, the proteasome (i.e., the major degradation machinery in the cytosol) protects the cell from proteotoxic stress. An increase in proteasomal activity by overexpression of the proteasome subunit RPN-6.1 resulted in life span extension in *C*. *elegans* [41]. Thus, mild mitochondrial dysfunction and proteasome activation both correlated with the positive regulation of longevity. The present findings reveal a connection between proteasome activation and mitochondrial dysfunction that is relevant to life span extension.

Our data suggest that impairments in mitochondrial import machinery can activate a universal stress response that results in an increase in the activity of cytosolic degradation machinery, namely the proteasome. To date, this phenomenon has been described only in yeast and named unfolded protein response activated by protein mistargeting (UPRam) [34]. The UPRam was defined as a response to the accumulation of mitochondrial precursor proteins in the cytosol, one characteristic feature of which is activation of the proteasome at the level of proteasome complex rearrangements. Nonimported mitochondrial precursor proteins can also cause clogging of the main gateway to mitochondria, the TOM translocase. In yeast, clogging of the translocase activated a transcriptional response that induced the clearance and proteasomal degradation of proteins that failed to be imported [67]. In our experimental model, we did not observe transcriptional reprogramming connected with proteasomal degradation, although we do not exclude possible contributions of transcriptional responses to this mechanism. In *C*. *elegans*, we showed that the depletion of DNJ-21 (i.e., the import motor that is associated with TIMM-23 during *C*. *elegans* development) led to activation of the 26S proteasome. Proteasome activation was not mediated by an increase in the expression of proteasome subunits. Thus, the UPRam exerts beneficial effects that lead to life span prolongation in higher eukaryotes.

Consistent with earlier findings [29], the depletion of DNJ-21 activated the *hsp-6* promoter in a *C*. *elegans* reporter strain. The *hsp-6* promoter drives expression of the mitochondrial HSP70 chaperone, and its activity is frequently used as readout for the transcriptional function of ATFS-1 in the nucleus.

The UPRam is a stress response mechanism that primarily adjusts cellular protein homeostasis in response to mitochondria-derived proteotoxic stress that occurs in the cytosol by modulating posttranscriptional responses. Accumulating nonimported mitochondrial proteins cause a proteotoxic stress in the cytosol. In the case of insufficient mitigation mechanisms, this can lead to acceleration of the formation of toxic protein aggregates in yeast and *C*. *elegans* [68]. In contrast, the UPRmt is activated upon the accumulation of unfolded proteins inside mitochondria, which triggers a transcriptional program to restore mitochondrial protein homeostasis and is mediated by the transcription factor ATFS-1. The activity of ATFS-1 contributes to the beneficial effect that is observed upon DNJ-21 depletion but importantly via a mechanism that does not involve the transcription of proteasomal genes. A decrease in mitochondrial import inhibits the translocation of ATFS-1 into the organelle [28]. Although a mechanistic role for ATFS-1 during proteasome activation remains to be determined, the lack of imported ATFS-1 might act as an additional stress signal for activation of the UPRam in *C*. *elegans*.

Mitochondrial dysfunction is linked to aging and neurodegeneration in humans. The importance of degradative cleaning pathways in limiting mitochondrial damage during cellular stress and aging is evident from model systems. Severely damaged mitochondria are degraded by an autophagy process to preserve cellular function [69]. Surveillance strategies involve the ubiquitin proteasome system that targets ubiquitinated outer membrane and retro-translocated mitochondrial proteins [33,70]. Our findings reveal a possible way in which such a protein-degradative pathway can be regulated to exert a beneficial response at the organism level.

## Methods

### Worm maintenance and strains

Standard conditions were used to cultivate *C*. *elegans* [71]. Briefly, nematodes were grown at 20 °C on plates that contained nematode growth medium (NGM; 1 mM CaCl_2_, 1 mM MgSO_4_, 5 μg/ml cholesterol, 25 mM KPO_4_ buffer [pH 6.0], 17 g/L agar, 3 g/L NaCl, and 2.5 g/L peptone) seeded with the *E*. *coli* strain HB101 or OP50 as a food source. To synchronize the worms, they were treated with hypochlorite to release the embryos, washed several times with M9 buffer (3 g/L KH_2_PO_4_, 6 g/L Na_2_HPO_4_, 5 g/L NaCl, and 1 M MgSO_4_), and either placed directly on NGM plates or hatched overnight in M9 buffer. *C*. *elegans* strains that were used in this study are listed in S4 Table.

### Generation of plasmids

RNAi constructs that targeted the *dnj-21*, *atfs-1*, C47G2.3 *(timm-22)*, *timm-23*, F11C1.1, F42H10.2, *rpn-2*, *and rpt-4* genes were created by the PCR amplification of genes from cDNA pools that were generated from total RNA. The PCR product was digested with XhoI and KpnI restriction enzymes and cloned into the XhoI- and KpnI- digested L4440 vector. Cloned constructs were sequenced for insert verification. The construct that harbored *cox-5B* for RNAi was a gift from Aleksandra Trifunovic (University of Cologne, Germany). Constructs that targeted ZK616.2 and ZK616.3 were purchased from Open Biosystems.

The construct for the expression of GFP on the mitochondrial outer membrane in worm body wall muscles was created using the SLiCE method [72]. Briefly, codon optimized sequences [73] for GFP, a linker that contained the attB5 sequence, and the first 55 amino acids from TOMM-20 protein [74] were amplified by PCR and inserted into the pCG150 vector that contained the *myo-3* promoter and *unc-54* 3′-untranslated region sequence. The cloned construct was sequenced for insert verification.

### Proteomic sample preparation

Approximately 18,000 to 20,000 young adult (day 1 old) worms were washed from NGM plates with M9 buffer and frozen in liquid nitrogen. After thawing, samples were washed twice with 100 mM Tris buffer (pH 8.5). The worms were then suspended in 200 μl of 8 M Urea buffer (8 M Urea and 100 mM Tris [pH 8.5]) and probe sonicated 4 times for 10 s each. The lysate was centrifuged at 2,800 × *g* for 5 min at 4 °C. The pellet that contained debris was discarded. The supernatant was diluted 8 times with 100 mM Tris buffer and incubated overnight at 37 °C with sequencing grade modified trypsin (Promega, Madison, USA) in the presence of 10 mM TCEP and 40 mM chloroacetamide. The samples were acidified with 1% trifluoroacetic acid (TFA), loaded on 3 AttractSPE Discs Bio C18 (Affinisep, Petit-Couronne, Normandy, France), and desalted by a previously reported stage-tip protocol [75]. Peptides were eluted from the SPE discs in 60% acetonitrile in water, and the solvent was removed using a Savant SpeedVac Concentrator (Thermo Fisher Scientific, Massachusetts, USA). Before the liquid chromatography (LC)–MS measurements, the peptides were reconstituted in 0.1% FTA, 2% acetonitrile in water.

### LC–MS/MS analysis

Chromatographic separation was performed on an Easy-Spray Acclaim PepMap column (50 cm long × 75 μm inner diameter; Thermo Fisher Scientific, Massachusetts, USA) at 45 °C by applying 150 min acetonitrile gradients in 0.1% aqueous formic acid at a flow rate of 300 nl/min. Each sample was measured in duplicate. An UltiMate 3000 nano-LC system was coupled to a Q Exactive HF-X mass spectrometer via an easy-spray source (all Thermo Fisher Scientific). The Q Exactive HF-X was operated in data-dependent mode, with survey scans acquired at a resolution of 120,000 at *m/z* 200. Up to 15 of the most abundant isotope patterns with charges 2 to 5 from the survey scan were selected with an isolation window of 1.3 m/z and fragmented by higher-energy collision dissociation (HCD) with normalized collision energies of 27, and the dynamic exclusion was set to 40 s. The maximum ion injection times for the survey scan and MS/MS scans (acquired at a resolution of 15,000 at *m/z* 200) were 45 and 22 ms, respectively. The ion target value for MS was set to 3e6 and for MS/MS was set to 4.4e2, and the intensity threshold for MS/MS was set to 2.0e4.

### Proteomics data processing

The data were processed using MaxQuant v. 1.6.10.43 software [76]. Peptides were identified from MS/MS spectra that were searched against the Uniprot *C*.*elegans* reference proteome (UP000001940) using the built-in Andromeda search engine. Cysteine carbamidomethylation was set as a fixed modification, and methionine oxidation and protein N-terminal acetylation were set as variable modifications. For in silico digests of the reference proteome, cleavages of arginine or lysine followed by any amino acid were allowed (trypsin/P), and up to 2 missed cleavages were allowed. The false discovery rate (FDR) was set to 0.01 for peptides, proteins, and sites. Match between runs was enabled. Other parameters were used as preset in the software. Unique and razor peptides were used for quantification to enable protein grouping (razor peptides are the peptides that are uniquely assigned to protein groups and not to individual proteins). The data were further analyzed using Perseus version 1.6.6.0 and Microsoft Office Excel 2016 software.

### LFQ-based differential analysis of protein levels

LFQ values for protein groups were loaded into Perseus v. 1.6.6.0 software [77]. Standard filtering steps were applied to clean up the dataset: Reverse (matched to decoy database), only identified by site, and potential contaminant (from a list of commonly occurring contaminants that are included in MaxQuant) protein groups were removed. Protein groups that were identified by less than 2 razor + unique peptides were removed. LFQ intensities were log2 transformed. Protein groups with LFQ values in less than 3 of 6 samples were removed, and all of the remaining missing values were imputed from the normal distribution (width = 0.3, down shift = 1.8 × standard deviation). The Gaussian distribution of log2-transformed LFQ intensities post-imputation was confirmed for each sample by histogram analysis. Student *t* tests (permutation-based FDR with 250 randomizations = 0.01, S0 = 0.1) were performed on the dataset to return proteins whose levels were statistically significantly changed in response to *dnj-21* knockdown (S1 Table).

### RNA interference

Synchronized *C*. *elegans* were fed *E*. *coli* HT115(DE3) that were transformed with a construct that targeted a specific gene or with the empty L4440 vector as a control. Bacterial cultures with an OD600 in the range 0.4 to 0.6 were induced with 1 mM IPTG for 2 h. When 2 different RNAi conditions were used for the knockdown of multiple genes (or diluted with the EV), bacterial cultures were mixed 1:1 according to their OD600 after induction with IPTG. For liquid culture, the bacterial pellet was added to S-medium [78] and used for further worm cultures. For cultures on solid medium, bacteria were seeded on standard NGM plates that were supplemented with 1 mM IPTG, 12.5 μg/ml tetracyclin, and 100 μg/ml ampicillin. Plates that were seeded with RNAi bacteria were used within 1 week after preparation.

### *C*. *elegans* transformation

The *C*. *elegans* transgenic strain ACH89 (see S4 Table) was created using biolistic bombardment as described previously [79]. The *unc-119* rescue was used as a selection marker.

### Life span and health span assays

Synchronized worms were grown on RNAi plates throughout the life span (approximately 30 worms per plate and for health span assay approximately 10 animals per plate) measurements. The first day of adulthood was set as day 0 of aging. The worms were transferred daily to fresh plates during the egg-laying period, after which time they were transferred every 2 to 3 days. During the life span assays, the worms were examined every day for touch-provoked movement and pharyngeal pumping until death. Worms that crawled off the plate were killed during the transfer process, or exhibited phenotypes of bagging phenotype or protruding vulva were censored from the experiments.

During the health span assays on days 1, 5, and 15 of adulthood, movements were recorded for 5 min using the WormLab Imaging System (MBF Bioscience, Williston, Vermont, USA). The frame rate, exposure time, and gain were set to 7.5 frames per second, 0.0031 s, and 1 s, respectively. Next, recorded worms were tracked using WormLab software (MBF Bioscience), and their health span was assessed based on their distance traveled, direction of movement, and overall movement (e.g., the number of reversals or omega turns).

### Total protein isolation and western blot

Total protein was isolated from frozen worm pellets. Samples were thawed on ice in lysis buffer (20 mM Tris [pH 7.4], 200 mM NaCl, and 2 mM phenylmethylsulfonyl fluoride [PMSF]) and sonicated 3 times for 10 s each. The lysate was centrifuged at 2,800 × *g* for 5 min at 4 °C. The pellet that contained debris was discarded. Protein concentrations were measured using the DirectDetect system (Millipore, Billerica, MA, USA). Proteins were separated by SDS-PAGE (using 15% acrylamide gels) and transferred to polyvinylidene difluoride [PVDF] membranes. Immunodetection was performed using standard chemiluminescence techniques. Primary antibodies against DNJ-21, TOMM-40, TIMM-23, and SCPL-4 were custom made in rabbit, and antisera were used at a concentration of 1:500. The primary antibody against ATP5B (which detects *C*. *elegans* ATP-2) was custom made and suitable for the analysis of complexes using native PAGE (gift from Peter Rehling). Other primary antibodies were purchased: tubulin (Sigma, catalog no. T9026), PAS-7 (Developmental Studies Hybridoma Bank [DSHB], catalog no. AB_10571458), ATP-5A (Abcam, catalog no. ab14748; detects *C*. *elegans* ATP-2); anti-Proteasome 20S alpha 1+2+3+5+6+7 antibody [MCP231] (PAS-[1-7] in *C*. *elegans*) (Abcam, catalog no. ab22674), GFP (Sigma, catalog no. 11814460001), ubiquitin (Santa Cruz, catalog no. sc-8017), NDUFS3 (Abcam, catalog no. ab14711; detects *C*. *elegans* NDUF-3), NDUFS1 (Santa Cruz, catalog no. sc-50132, detects *C*. *elegans* NDUF-1), cytochrome C (Abcam, catalog no. ab110325), and actin (Millipore, catalog no. MAB1501). Unprocessed images of western blots are presented in S1 Raw Images.

### Mitochondria isolation and analysis by high-resolution respirometry

Worms were washed with M9 buffer, and the pellet was resuspended in 5 ml of homogenization buffer (220 mM mannitol, 70 mM sucrose, 10 mM Tris [pH 7.4], 2 mM ethylenediaminetetraacetic acid [EDTA], and 2 mM PMSF). To obtain a mitochondrion-enriched fraction, a Balch homogenizer (Isobiotec, Heidelberg, Germany) was used. Nematodes were gently passed through the homogenizer chamber with 1 ml syringes 5 times. To fracture the nematode cuticle, a 12-μm ball clearance was applied. The homogenate was centrifuged at 800 x *g* for 5 min at 4 °C to sediment debris and larger worm fragments. The mitochondria-containing supernatant was collected and centrifuged at 9,000 x *g* for 10 min at 4 °C, and the pellet was resuspended in 300 μl of MIR05 mitochondrial respiration medium (0.5 mM ethyleneguanosinetetraacetic acid, 3 mM MgCl2, 60 mM lactobionic acid, 20 mM taurine, 10 mM KH2PO4, 110 mM sucrose, 0.1% bovine serum albumin, and 20 mM HEPES/KOH [pH 7.1]). Isolated mitochondria (approximately 0.300 μg) were added to an Oxygraph-2k respirometer (Oroboros, Innsbruck, Austria) that contained MIR05 medium at 20 °C. Complex I OXPHOS was evaluated after sample oxygen consumption stabilization in an Oxygraph-2k chamber. Complex I was stimulated by Malate (0.5 mM), pyruvate (5 mM), and glutamate (10 mM) in the presence of ADP+Mg+2 (2.5 mM). Complex II OXPHOS was evaluated after complex I inhibition by rotenone (0.05 μM) followed by succinate (10 mM) stimulation. At this point, the outer membrane integrity of mitochondria was assessed by cytochrome c (10 μM). Complex IV oxygen consumption was evaluated after complex III inhibition by antimycin A (2.5 μM), supported only by the remaining activity of complex II. Complex IV was stimulated by ascorbate (2 mM) and N,N,N′,N′-tetramethyl-p-phenylenediamine dihydrochloride (TMPD; 0.5 mM). Measurements were concluded after complex IV inhibition by sodium azide (50 mM). The data were analyzed using DatLab 7 software (Oroboros, Innsbruck, Austria).

### Mitochondria isolation and analysis of respiratory chain complexes by native electrophoresis

Worms were washed with M9 buffer, and the pellet was resuspended in 5 ml of homogenization buffer (220 mM mannitol, 70 mM sucrose, 10 mM Tris [pH 7.4], 2 mM EDTA, and 2 mM PMSF). Worms were homogenized in a glass homogenizer (15 strokes; Sartorius, catalog no. BBI-8540705), and the total volume was increased to 10 ml with homogenization buffer. The lysate was centrifuged at 1,000 x *g* for 5 min at 4 °C. The pellet was resuspended in another 5 ml of homogenization buffer, and the homogenization procedure was repeated. After a clearing spin, supernatants that contained mitochondria were combined and spun at 12,000 x *g* for 10 min at 4 °C. The pellet was resuspended in 10 ml of homogenization buffer and spun at 10,000 x *g* for 5 min at 4 °C. The resulting pellet that contained mitochondria was resuspended in a buffer that contained 250 mM sucrose and 10 mM Tris (pH 7.4), frozen in liquid nitrogen, and stored at −80 °C.

To analyze respiratory chain complexes, mitochondria were suspended at a concentration of 1 mg/ml in solubilization buffer (1.5 M aminocaproic acid and 50 mM Bis-Tris–HCl [pH 7.0]) that contained 0.5% n-dodecyl-β-D-maltoside (DDM) for 5 min at 4 °C. The mitochondrial suspension was mixed by gentle pipetting and incubated at 4 °C for 5 min. After lysis, the cell suspension was centrifuged at 20,000 x *g* for 15 min at 4 °C. The supernatant was transferred to a prechilled Eppendorf tube, 10× loading dye (500 mM ε-amino-N-caproic acid, 100 mM Bis-Tris, and 5% Coomassie G-250) was added, and the sample was spun at 20,000 × *g* for 15 min at 4 °C. The sample was directly loaded into a 4% to 13% gradient polyacrylamide gel and resolved at 4 °C. Protein complexes were transferred to PVDF membranes and immunodetected with specific antibodies. The High Molecular Weight Calibration Kit for native electrophoresis (Amersham, GE Healthcare, Little Chalfont, UK) was used as a molecular weight standard.

### Adenosine triphosphate measurement

The protocol was based on a previous study [80], with some modifications. Briefly, 50 μl of worms were collected in 50 μl of sterile M9 buffer and frozen in liquid nitrogen. Samples were stored at −80 °C. Worms were thawed and incubated in a thermoblock at 99 °C for 15 min, after which they were incubated for 5 min on ice. Samples were centrifuged at 14,800 × *g* for 10 min at 4 °C. The supernatant was transferred to fresh tubes and kept on ice. Protein concentrations were measured using the DirectDetect system. ATP levels were measured in a 96-well plate using the ATP Bioluminescence Assay Kit CLS II (Roche, Mannheim, Germany). Worm lysates (50 μg) were transferred to the plate, and luciferase buffer was added. Each sample was measured using at least 3 technical replicates. A standard curve for ATP was prepared for each experiment. The results were normalized to the protein concentration, and ATP content was calculated using the ATP standard curve.

### Gene expression analysis

To assess gene expression levels, synchronized young adult worms were grown and frozen in liquid nitrogen. RNA extraction was performed according to a previously published method, with minor modifications [49]. Briefly, samples were collected and immediately snap frozen in liquid nitrogen. The following steps were performed after the samples were stored at −80 °C. Samples were resuspended in 500 μl of Trizol (catalog no. 10296010, Thermo Fisher). After 3 freeze–thaw cycles, 100 μl of chloroform (Merck, catalog no. C2432) was added. After centrifugation, the upper phase was collected and mixed with the same volume of 70% ethanol. The RNeasy Plus Mini Kit (Qiagen, catalog no. 74134) was used for further total RNA isolation according to the manufacturer’s instructions. Total RNA (500 ng) was used to generate cDNA using the SuperScript IV First-Strand Synthesis System (Invitrogen, catalog no. 18091050, Thermo Fisher). RT-qPCR was performed using SensiFAST SYBR Hi-ROX mix (2x; Bioline, catalog no. BIO-92020) in a LightCycler480 (Roche) with a 96-well white plate (Roche, catalog no. 4729692001). Fold changes in mRNA expression of the target genes were calculated using the ΔΔCt method. The mean expression levels of *act-1* and *cdc-42* were used as internal controls. The data are presented as mean ± SD (*n* = 3). The primers that were used in this study are shown in S5 Table.

### Proteasomal activity assay and native gel electrophoresis of proteasome complexes

To assess proteasomal activity in whole worm lysates, synchronized young adult worms were sonicated in proteasomal activity lysis buffer (50 mM Tris-HCl [pH 7.4], 100 mM ATP, 5 mM MgCl_2_, 250 mM sucrose, 0.5 mM EDTA, 2 mM ATP, and 1 mM DTT). The lysate was cleared by centrifugation at 18,000 × *g* for 15 min at 4 °C and used immediately for further measurements. Chymotrypsin-like activity was analyzed in the presence of 5 mM of the fluorogenic peptide Suc-LLVY-AMC (Bachem, catalog no. 4011369). Specific activity was determined in the presence of 50 μM MG132 (Enzo Life Sciences, catalog no. BML-PI102). Fluorescence was measured using a fluorescence spectrophotometer (Hybrid Multi-Mode Reader Synergy, catalog no. H1MFDG) in 5-min intervals for a total of 120 min. To assess proteasomal activity in the mitochondrial fraction, mitochondria were isolated as described above for the BN-PAGE analysis. The pellet that contained mitochondria was resuspended in proteasomal activity lysis buffer, and samples were analyzed for chymotrypsin-like activity as described above.

For native gel electrophoresis, synchronized worms were collected, washed, resuspended in proteasomal activity lysis buffer, and homogenized by sonication. Protein was separated on native 4% polyacrylamide gel that was supplemented with 5 mM MgCl_2_ and 1 mM ATP. Electrophoresis was performed at 4 °C for 3.5 h at 30 mA using running buffer (90 mM Tris, 90 mM boric acid, 5 mM MgCl_2_, and 1 mM ATP).

### Analysis of proteasomal activity using the UbG76V-Dendra2 strain

Freshly starved L1 larvae that expressed UbG76V-Dendra2 protein in body wall muscles were placed on RNAi plates. When they reached the L4 larval stage, the worms were transferred to single plates. On the next day, young adult worms were exposed to blue light for 60 s to photoconvert green UbG76V-Dendra2 protein into its red variant. Photoconversion was performed using a Leica M165FC stereomicroscope that was equipped with a Leica EL6000 lamp and standard GFP filter set (Chroma, Vermont, USA). Directly after photoconversion (t = 0), the red fluorescent signal from the body wall muscles was captured using the aforementioned stereomicroscope that was equipped with a Leica DFC365 FX CCD camera and standard Texas Red filter set (Chroma). The exposure time was 200 ms, and the gain was set to 2, which allowed for minimum blurring and did not cause photobleaching. Eighteen hours later, measurements were repeated (t = 18 h), and the fluorescent signal from the head region was analyzed using Las X software. Proteasomal activity was calculated as the ratio between the red fluorescent signal at t = 18 h and t = 0 and normalized to the signal from worms that were grown on control RNAi plates.

### Microscopy analysis

To investigate mitochondrial morphology, we used a transgenic worm strain (ACH89) that expressed GFP attached to the mitochondrial outer membrane in body wall muscles. Fluorescence images of muscle cells from the middle part of the worm’s body were captured using a Zeiss 700 laser-scanning confocal microscope with a 40× oil objective (NA 1.3), PMT detector, and 488 nm laser for GFP excitation. Worm muscular mitochondria were visualized using a 1AU confocal pinhole, and z-stacks with 21 focal planes were acquired, each with an optical thickness of 0.5 μm. Next, z-stacks were merged using Maximum Intensity Projection. Images were processed using ImageJ software. Mitochondrial morphology was analyzed using the ImageJ Fiji plugin tool “Mitochondria Analyzer” version 2.0.2 (https://sites.imagej.net/ACMito/). At least 2 regions of interest in at least 10 images of worms per condition were analyzed.

Live images of worms that expressed transcriptional reporters for stress responses were taken either when the worms were on plates or of single worms at higher magnification. For the latter, worms were immobilized in 20 mM sodium azide on an agar pad. Worms were observed under a Zeiss Axio Observer.D1 microscope. The acquisition time was set to 300 ms with no gain or image enhancement.

### Evolutionary analysis

Evolutionary history was inferred using the Maximum Likelihood method and Whelan And Goldman model [81]. The bootstrap consensus tree, inferred from 1,000 replicates, is taken to represent evolutionary history of the analyzed taxa [82]. Branches that correspond to partitions that are reproduced in less than 50% bootstrap replicates are collapsed. The percentage of replicate trees in which the associated taxa clustered together in the bootstrap test (1,000 replicates) are shown next to the branches [82]. Initial trees for the heuristic search were obtained automatically by applying the Neighbor-Join and BioNJ algorithms to a matrix of pairwise distances that were estimated using the JTT model, followed by selecting the topology with a superior log likelihood value. This analysis involved 6 amino acid sequences. There were a total of 126 positions in the final dataset. Evolutionary analyses were conducted using MEGA X [83].

### Statistical analysis

Proteasomal activity measurements and health span assessments were analyzed using two-tailed, unpaired *t* tests, assuming equal or unequal variance. Values of *p* ≤ 0.05 were considered statistically significant. Survival curves were created using the product-limit method of Kaplan and Meier. The log-rank (Mantel-Cox) test was used to evaluate differences between survival curves and to determine *p*-values for all independent data [84].

We reanalyzed the RNA sequencing data that were originally published by Wu and colleagues [47] using the web-based tool “NetworkAnalyst” (https://www.networkanalyst.ca) [85]. Differentially expressed genes were obtained from original gene expression tables using Limma statistical analysis and specific comparisons between all wild-type samples and all *atfs-1* mutant samples.

## Supporting information

S1 Fig Mild mitochondrial defect upon DNJ-21 depletion. (A, B) Phenotypic analysis of parental (P0) and F1 progeny upon DNJ-21 depletion. Wild-type worms were fed RNAi bacteria starting from the first larval stage. (A) The brood size of at least 8 worms per condition was counted. Data are presented as mean ± 95% confidence level. ***p < 0.001. (B) Emb and Lva/Lvl of F1 progeny were counted. The data are expressed as a percentage of the total number of embryos (*n*) with 95% confidence level. *n* (EV) = 2,701. *n* (*dnj-21* RNAi) = 1,949. ***p < 0.001. (C) Isolated mitochondria from 3 biological replicates that were used for the analysis of high-resolution respirometry were solubilized, subjected to SDS-PAGE, and analyzed by western blot using specific antibodies. (D, E) Quantification of densitometry measurements of signals that are shown in panel (C) for DNJ-21 (D) and NDUF-3 (E), normalized to the signal of ATP-2. Data are presented as mean ± SD (*n* = 4). **p < 0.01. (F) Illustration of proteomics approach. Synchronized wild-type worms were grown from the first larval stage on RNAi bacteria and harvested at the young adult stage. The experiment was repeated in 3 biological replicates. (G, H) Distribution of fold change on protein levels filtered by specific GO terms (blue circles). Proteomics data are also presented in S1 Table and PXD023830. Underlying numerical data are presented in S1 Data. Emb, embryonic lethality; EV, empty vector; GO, Gene Ontology; Lva/Lvl, larval arrest/lethality; MS/MS, tandem mass spectrometry; ns, not significant; RNAi, RNA interference.
(TIF)

S2 Fig Depletion of mitochondrial import components can prolong life. (A) Isolated mitochondria were solubilized, subjected to SDS-PAGE, and analyzed by western blot using specific antibodies. (B) Survival curve of wild-type worms that were treated with RNAi as indicated. Life span values are presented in S2 Table. (C) Protein sequence alignment of putative homologs of MIA40 and MIA40 from *H*. *sapiens* and *S*. *cerevisiae*. The N-terminal part of ScMia40 was omitted for alignment because it contains a transmembrane part that is specific only for yeast MIA40. Classic, conserved cysteine-residue motives are indicated. (D) Phylogenetic tree of putative homologs of *C*. *elegans*. The percentage of replicative trees is indicated on the branches. (E, F) Survival curve of the RNAi-sensitive *rrf-3* mutant that was treated with RNAi as indicated. Life span values are presented in S2 Table. (G, H) The worm population was assayed for movement behavior, showing the forward movement percentage (G) and number of reversals (H) on the indicated days, starting on the first day of the reproductive phase. The data are expressed as mean ± SEM. *n* = 12–18 (*n* indicates the number of worms). Two biological replicates were performed for each condition. *p < 0.05, **p < 0.01. Underlying numerical data are presented in S1 Data. EV, empty vector; MIA40, mitochondrial intermembrane space import and assembly protein 40; RNAi, RNA interference.
(TIF)

S3 Fig Activation of stress responses upon DNJ-21 depletion. (A) The strain that expressed the transcriptional reporter for activation of the UPRmt (*phsp-6*::*gfp*) was cultured on plates with RNAi bacteria as indicated from the L1 larval stage to day 1 of adulthood. Fluorescent images with the same exposure time are shown. The exposure time was adjusted to minimize the time necessary to detect background fluorescence in control worms (EV). (B) The strain that expressed the transcriptional reporter for activation of the UPR_ER_ (*phsp-4*::*gfp*) was cultured on plates with RNAi bacteria as indicated from the L1 larval stage to young adulthood. Tunicamycin treatment was performed in liquid for 4 h when worms were young adults. Fluorescent images were taken with the same exposure time. Scale bar = 500 μm. Microscopy analysis was repeated in 2 biological replicates. (C) RT-qPCR in wild-type worms that were kept on *dnj-21* RNAi or an EV control from the embryonic stage until young adulthood. The mRNA levels are presented as fold changes relative to the respective EV control (mean ± SD). The qPCR analysis was repeated in 3 biological replicates. ***p < 0.005. Mann–Whitney *U* test was used for statistical analysis. (D, E) Distribution of fold changes in protein levels filtered by specific GO terms (blue and red circles). Proteomics data are also presented in S1 Table and PXD023830. Underlying numerical data are presented in S1 Data. DMSO, dimethylsulfoxide; EV, empty vector; GFP, green fluorescent protein; GO, Gene Ontology; RNAi, RNA interference; RT-qPCR, quantitative real-time PCR; UPR_ER_, endoplasmic reticulum unfolded protein response; UPRmt, mitochondrial unfolded protein response.
(TIF)

S4 Fig Proteasomal activity upon DNJ-21 depletion. (A) Measured proteasome activity (shown as UbG76V-Dendra2 fraction that degraded after 18 h) does not correlate with expression of the reporter (shown as its fluorescence intensity). The gray line is the linear regression lines. Each gray point represents one worm. *n* = 40. (B) Dendra2 tagged with UbG76V efficiently measures protein degradation. The data are expressed as mean ± SEM. *n* = 9–40 (*n* represents the number of individual worms analyzed). ***p < 0.001. Underlying numerical data are presented in S1 Data. EV, empty vector; RNAi, RNA interference.
(TIF)

S5 Fig Life span upon DNJ-21 depletion also depends on ATFS-1 function. (A) Quantification of proteasomal activity upon the depletion of DNJ-21 only or 1:1 diluted with bacteria that contained empty plasmid. The data are expressed as mean ± SEM. *n* = 29–40 (*n* represents the number of individual worms analyzed). *p < 0.05. (B, D) Survival curve of wild-type or mutant worms that were treated with RNAi as indicated. Life span values are presented in S2 Table. (C) Quantification of proteasomal activity upon *cox-5B* or *mrps-5* depletion. Shown are the ratios of t = 18 h/t = 0 that were normalized to the EV control. The data are expressed as mean ± SEM. *n* = 36–40 (*n* represents the number of individual worms analyzed). Underlying numerical data are presented in S1 Data. ATFS-1, activating transcription factor associated with stress 1; EV, empty vector; RNAi, RNA interference.
(TIF)

S6 Fig Meta-analysis of RNA sequencing data depending on ATFS-1 function. The data are from Wu and colleagues [47]. (A) Schema of the localization of ATFS-1 depending on *atfs-1* mutation. (B, C) The expression of proteasome subunits tends to decrease in the *atfs-1* gain-of-function mutant. (D) The expression of proteasome subunits tends to increase in the *atfs-1* deletion mutant. Underlying numerical data are presented in S1 Data. ATFS-1, activating transcription factor associated with stress 1; EV, empty vector; MTS, mitochondrial targeting sequence; NLS, nuclear localization signal; UPRmt, mitochondrial unfolded protein response; WT, wild type.
(TIF)

S7 Fig Analysis of proteasome composition depending on ATFS-1 function. (B, E, F, and H) Total worm lysates were separated by SDS-PAGE and analyzed by western blot using ubiquitin or specific antibodies. (A) Total worm lysates were separated by native PAGE and analyzed by western blot. Equal loading was controlled by Coomassie staining. (B, E) Western blot analysis was repeated in at least 3 biological replicates. (C) Chymotrypsin-like proteasome activity measurement in the fraction of isolated mitochondria. The data are expressed as mean ± SEM. *n* = 7. *p = 0.02. (D) Synchronized worms were cultured in liquid medium that contained RNAi bacteria. Chymotrypsin-like activity was measured with fluorogenic peptides. The data are expressed as mean ± SD. *n* = 3. (F–H) Synchronized populations of WT worms and mutants of *atfs-1* were cultured on plates that were seeded with HT115(DE3) bacteria. Total worm extracts were separated by SDS-PAGE or native PAGE (G) and analyzed by western blot using specific antibodies. Western blot analysis was repeated in 2 biological replicates. Underlying numerical data are presented in S1 Data. ATFS-1, activating transcription factor associated with stress 1; EV, empty vector; ns, not significant; RNAi, RNA interference; WT, wild type.
(TIF)

S8 Fig Analysis of dysfunctional proteasome mutants. (A, B) Total worm lysates were separated by SDS-PAGE or native PAGE and analyzed by western blot. Western blot analysis was repeated in at least 3 biological replicates. (C) Synchronized worms were fed empty HT115(DE3) bacteria. Proteasomal activity was measured with fluorogenic peptides. The data are expressed as mean ± SD. *n* = 2. (D) WT and *rpn-10* deletion mutant worms were fed bacteria that contained indicated RNAi from the L1 larval stage until young adulthood. RT-qPCR was performed to quantify levels of the indicated mRNAs. **p < 0.01. Kruskal–Wallis test was used for statistical analysis. The qPCR analysis was repeated in 3 biological replicates. (E) Worms were kept on RNAi plates throughout the experiment. Survival curves upon the depletion of DNJ-21 depending on RPN-10 and ATFS-1 function are shown. Life span values are presented in S2 Table. (F) Representative images of worms according to short and long RNAi treatment schema (see also Fig 7C). Images were taken at the same magnification as worms that were continuously kept on control plates (EV) until adulthood. Underlying numerical data are presented in S1 Data. ATFS-1, activating transcription factor associated with stress 1; EV, empty vector; ns, not significant; RNAi, RNA interference; RT-qPCR, quantitative real-time PCR; WT, wild type.
(TIF)

S1 Table Proteomics analysis upon *dnj-21* RNAi. Identification and quantification of protein levels via MS. Related to Figs 1I and 6A and S1F–S1H, S3C and S3D Figs. MS, mass spectrometry; RNAi, RNA interference.
(XLSX)

S2 Table Adult life span analysis. Related to Figs 2A–2C, 5D, 5E, 7B, 7E and 7F and S2B, S2E, S2F, S5B, S5D and S8E Figs.
(PDF)

S3 Table Targets of the UPRmt. Listed are proteins identified by MS (see S1 Table) upon *dnj-21* RNAi, which were shown previously in the literature to be up-regulated on the transcriptional level upon induction of the UPRmt. Related to S1 Table. MS, mass spectrometry; RNAi, RNA interference; UPRmt, mitochondrial unfolded protein response.
(PDF)

S4 Table *C*. *elegans* strains used in this study. (PDF)

S5 Table Primers used for gene expression analysis. (PDF)

S1 Data Numerical data underlying Figs 1D–1H, 2A–2D, 3C, 3E, 3F, 4C, 4D, 5B–5E, 6A, 6D, 7B, 7E and 7F and S1A, S1B, S1D, S1E, S1G, S1H, S2B, S2E–S2H, S3C, S3D, S4A, S4B, S5A–S5D, S6B–S6D, S7C, S7D and S8C–S8E Figs. (XLSX)

S1 Raw Images Uncropped blots underlying Figs 1A, 3B, 3D, 5A, 6B, 6C, 6E, 6F, 7A and 7D and S1C, S2A, S7A, S7B, S7E–S7H, S8A and S8B Figs. (PDF)

## Abbreviations

ATFS-1: activating transcription factor associated with stress 1
ATP: adenosine triphosphate
BN-PAGE: blue native polyacrylamide gel electrophoresis
DDM: n-dodecyl-β-D-maltoside
DMSO: dimethylsulfoxide
DSHB: Developmental Studies Hybridoma Bank
dsRNA: double stranded RNA
EDTA: ethylenediaminetetraacetic acid
EV: empty vector
FDR: false discovery rate
GFP: green fluorescent protein
GO: Gene Ontology
HCD: higher-energy collision dissociation
Hsp70: heat shock protein 70
HSR: heat shock response
IM: inner membrane
LC: liquid chromatography
Mia40: mitochondrial intermembrane space import and assembly protein 40
MS: mass spectrometry
NGM: nematode growth medium
OCR: oxygen consumption rate
OXPHOS: oxidative phosphorylation
PAM: presequence translocase-associated motor
PMSF: phenylmethylsulfonyl fluoride
PVDF: polyvinylidene difluoride
RNAi: RNA interference
RT-qPCR: quantitative real-time polymerase chain reaction
TFA: trifluoroacetic acid
TMPD: N,N,N′,N′-tetramethyl-p-phenylenediamine dihydrochloride
UPRam: unfolded protein response activated by mistargeting of proteins
UPR_ER_: endoplasmic reticulum unfolded protein response
UPRmt: mitochondrial unfolded protein response
