# Supplementary figures and images for: Proteasome activity contributes to pro-survival response upon mild mitochondrial stress in Caenorhabditis elegans
Source: PLoS Biol. 2021 Jul 12;19(7):e3001302. doi: 10.1371/journal.pbio.3001302 (PMC8274918; doi:10.1371/journal.pbio.3001302)

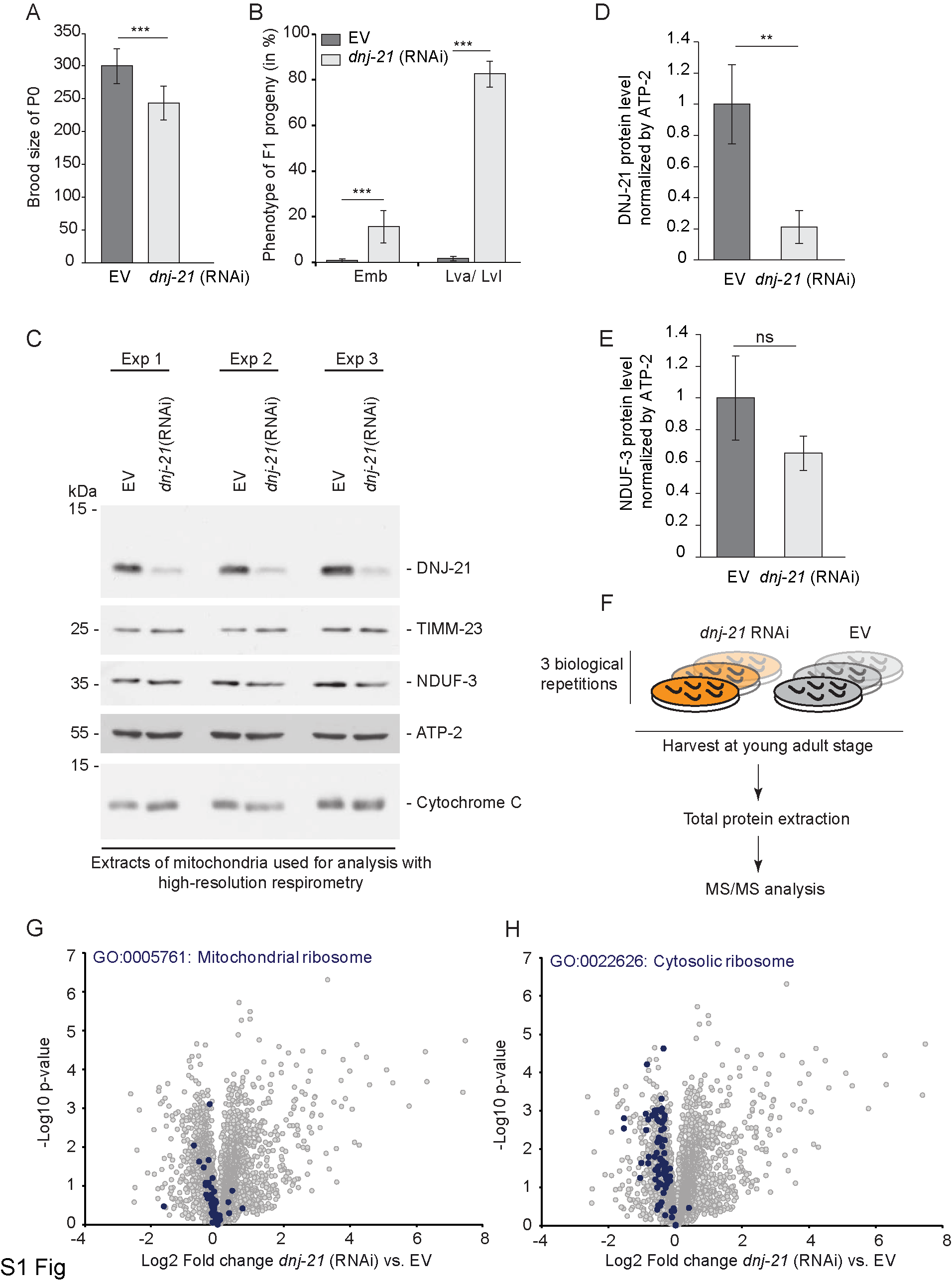

Supplement: S1 Fig — (A, B) Phenotypic analysis of parental (P0) and F1 progeny upon DNJ-21 depletion. Wild-type worms were fed RNAi bacteria starting from the first larval stage. (A) The brood size of at least 8 worms per condition was counted. Data are presented as mean ± 95% confidence level. ***p < 0.001. (B) Emb and Lva/Lvl of F1 progeny were counted. The data are expressed as a percentage of the total number of embryos (n) with 95% confidence level. n (EV) = 2,701. n (dnj-21 RNAi) = 1,949. ***p < 0.001. (C) Isolated mitochondria from 3 biological replicates that were used for the analysis of high-resolution respirometry were solubilized, subjected to SDS-PAGE, and analyzed by western blot using specific antibodies. (D, E) Quantification of densitometry measurements of signals that are shown in panel (C) for DNJ-21 (D) and NDUF-3 (E), normalized to the signal of ATP-2. Data are presented as mean ± SD (n = 4). **p < 0.01. (F) Illustration of proteomics approach. Synchronized wild-type worms were grown from the first larval stage on RNAi bacteria and harvested at the young adult stage. The experiment was repeated in 3 biological replicates. (G, H) Distribution of fold change on protein levels filtered by specific GO terms (blue circles). Proteomics data are also presented in S1 Table and PXD023830. Underlying numerical data are presented in S1 Data. Emb, embryonic lethality; EV, empty vector; GO, Gene Ontology; Lva/Lvl, larval arrest/lethality; MS/MS, tandem mass spectrometry; ns, not significant; RNAi, RNA interference. (TIF) [file pbio.3001302.s001.tif]

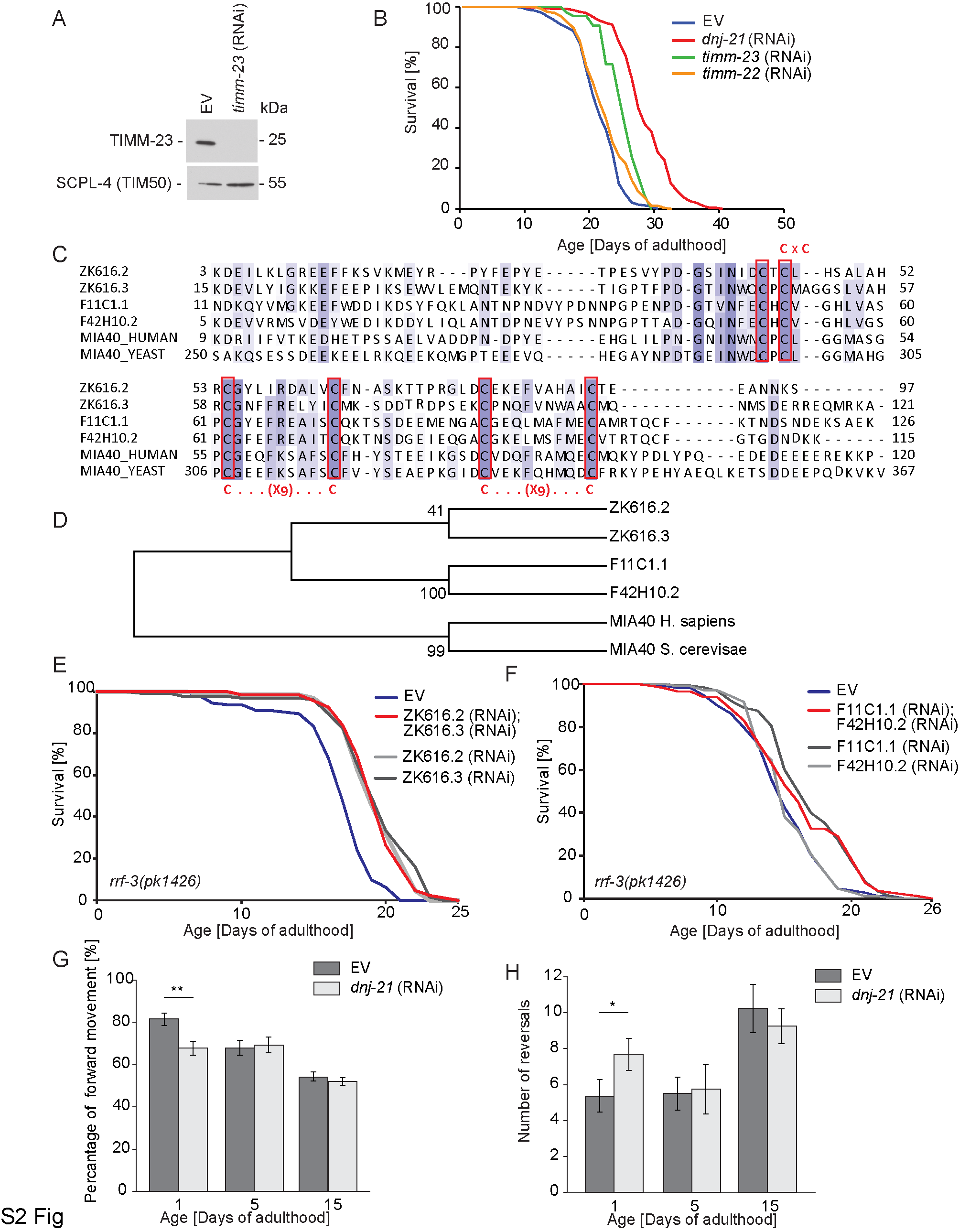

Supplement: S2 Fig — (A) Isolated mitochondria were solubilized, subjected to SDS-PAGE, and analyzed by western blot using specific antibodies. (B) Survival curve of wild-type worms that were treated with RNAi as indicated. Life span values are presented in S2 Table. (C) Protein sequence alignment of putative homologs of MIA40 and MIA40 from H. sapiens and S. cerevisiae. The N-terminal part of ScMia40 was omitted for alignment because it contains a transmembrane part that is specific only for yeast MIA40. Classic, conserved cysteine-residue motives are indicated. (D) Phylogenetic tree of putative homologs of C. elegans. The percentage of replicative trees is indicated on the branches. (E, F) Survival curve of the RNAi-sensitive rrf-3 mutant that was treated with RNAi as indicated. Life span values are presented in S2 Table. (G, H) The worm population was assayed for movement behavior, showing the forward movement percentage (G) and number of reversals (H) on the indicated days, starting on the first day of the reproductive phase. The data are expressed as mean ± SEM. n = 12–18 (n indicates the number of worms). Two biological replicates were performed for each condition. *p < 0.05, **p < 0.01. Underlying numerical data are presented in S1 Data. EV, empty vector; MIA40, mitochondrial intermembrane space import and assembly protein 40; RNAi, RNA interference. (TIF) [file pbio.3001302.s002.tif]

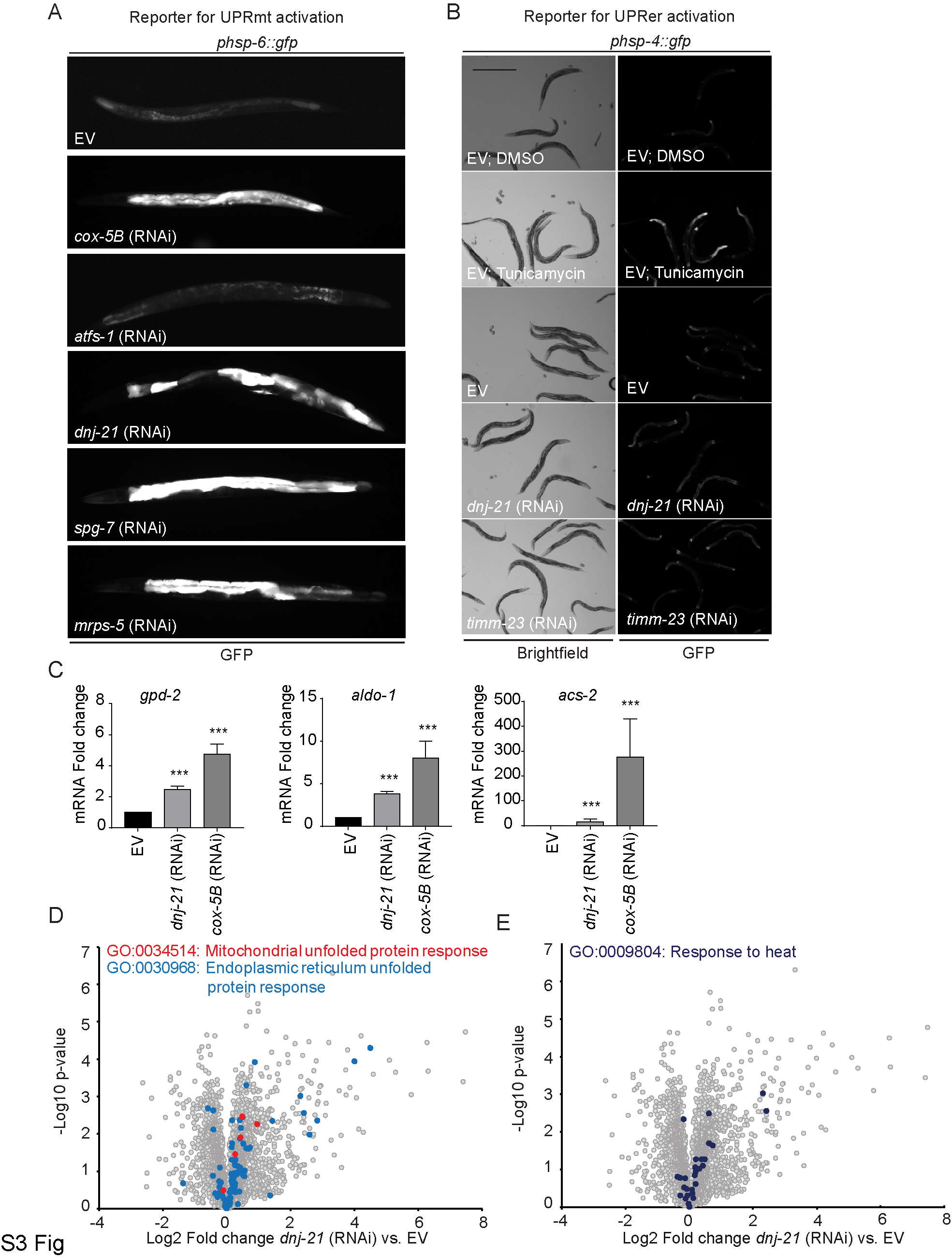

Supplement: S3 Fig — (A) The strain that expressed the transcriptional reporter for activation of the UPRmt (phsp-6::gfp) was cultured on plates with RNAi bacteria as indicated from the L1 larval stage to day 1 of adulthood. Fluorescent images with the same exposure time are shown. The exposure time was adjusted to minimize the time necessary to detect background fluorescence in control worms (EV). (B) The strain that expressed the transcriptional reporter for activation of the UPRER (phsp-4::gfp) was cultured on plates with RNAi bacteria as indicated from the L1 larval stage to young adulthood. Tunicamycin treatment was performed in liquid for 4 h when worms were young adults. Fluorescent images were taken with the same exposure time. Scale bar = 500 μm. Microscopy analysis was repeated in 2 biological replicates. (C) RT-qPCR in wild-type worms that were kept on dnj-21 RNAi or an EV control from the embryonic stage until young adulthood. The mRNA levels are presented as fold changes relative to the respective EV control (mean ± SD). The qPCR analysis was repeated in 3 biological replicates. ***p < 0.005. Mann–Whitney U test was used for statistical analysis. (D, E) Distribution of fold changes in protein levels filtered by specific GO terms (blue and red circles). Proteomics data are also presented in S1 Table and PXD023830. Underlying numerical data are presented in S1 Data. DMSO, dimethylsulfoxide; EV, empty vector; GFP, green fluorescent protein; GO, Gene Ontology; RNAi, RNA interference; RT-qPCR, quantitative real-time PCR; UPRER, endoplasmic reticulum unfolded protein response; UPRmt, mitochondrial unfolded protein response. (TIF) [file pbio.3001302.s003.tif]

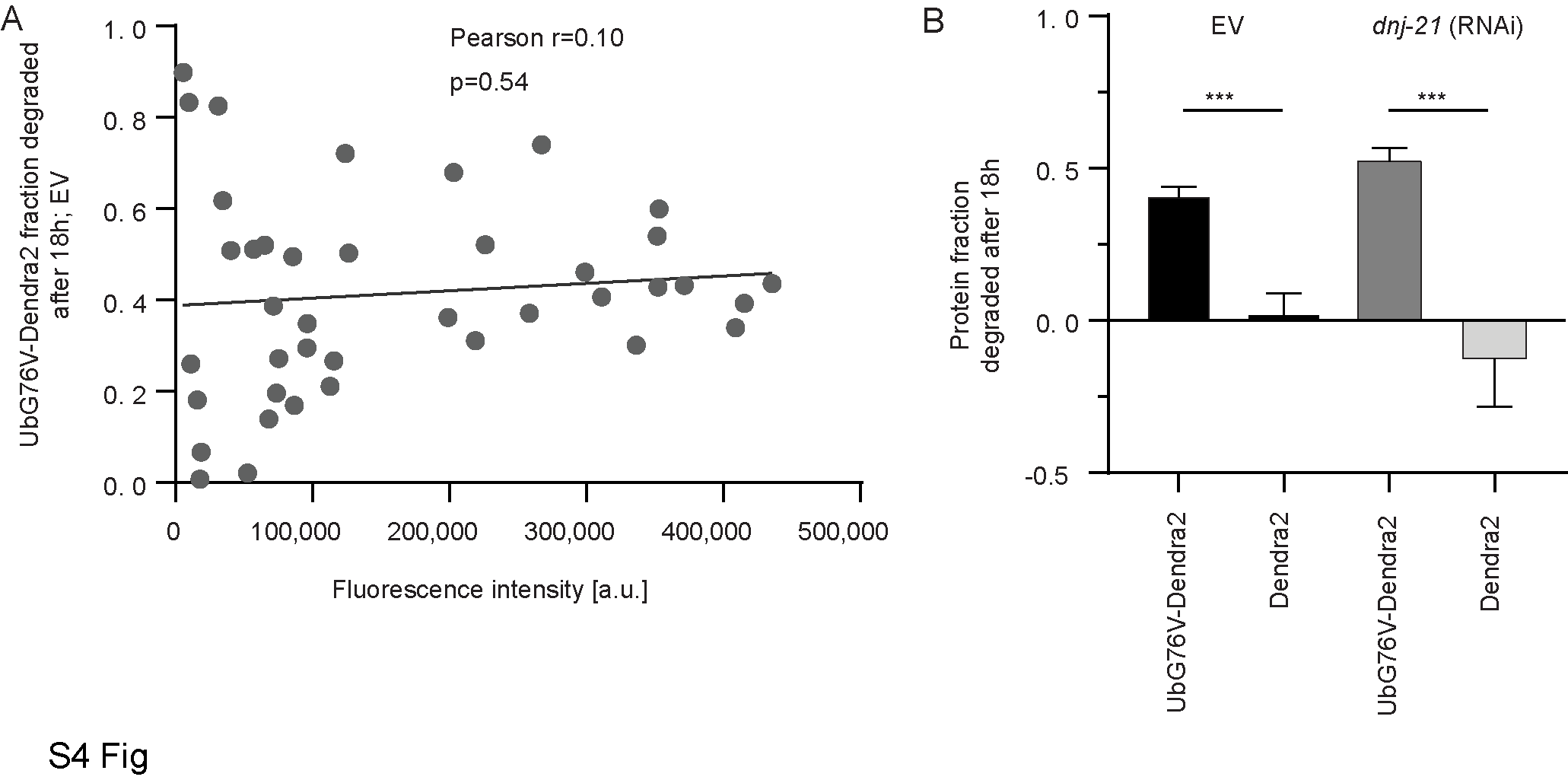

Supplement: S4 Fig — (A) Measured proteasome activity (shown as UbG76V-Dendra2 fraction that degraded after 18 h) does not correlate with expression of the reporter (shown as its fluorescence intensity). The gray line is the linear regression lines. Each gray point represents one worm. n = 40. (B) Dendra2 tagged with UbG76V efficiently measures protein degradation. The data are expressed as mean ± SEM. n = 9–40 (n represents the number of individual worms analyzed). ***p < 0.001. Underlying numerical data are presented in S1 Data. EV, empty vector; RNAi, RNA interference. (TIF) [file pbio.3001302.s004.tif]

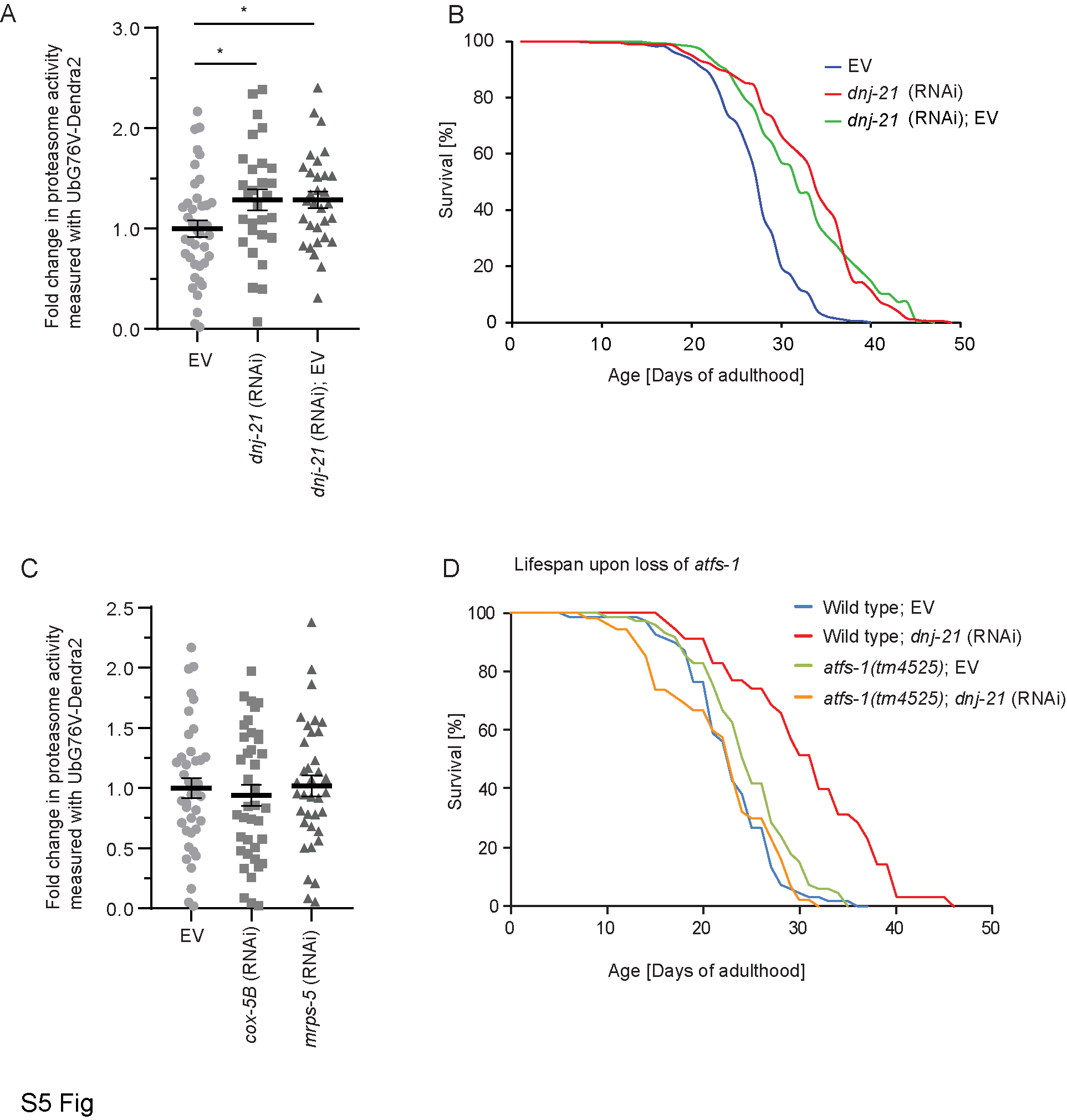

Supplement: S5 Fig — (A) Quantification of proteasomal activity upon the depletion of DNJ-21 only or 1:1 diluted with bacteria that contained empty plasmid. The data are expressed as mean ± SEM. n = 29–40 (n represents the number of individual worms analyzed). *p < 0.05. (B, D) Survival curve of wild-type or mutant worms that were treated with RNAi as indicated. Life span values are presented in S2 Table. (C) Quantification of proteasomal activity upon cox-5B or mrps-5 depletion. Shown are the ratios of t = 18 h/t = 0 that were normalized to the EV control. The data are expressed as mean ± SEM. n = 36–40 (n represents the number of individual worms analyzed). Underlying numerical data are presented in S1 Data. ATFS-1, activating transcription factor associated with stress 1; EV, empty vector; RNAi, RNA interference. (TIF) [file pbio.3001302.s005.tif]

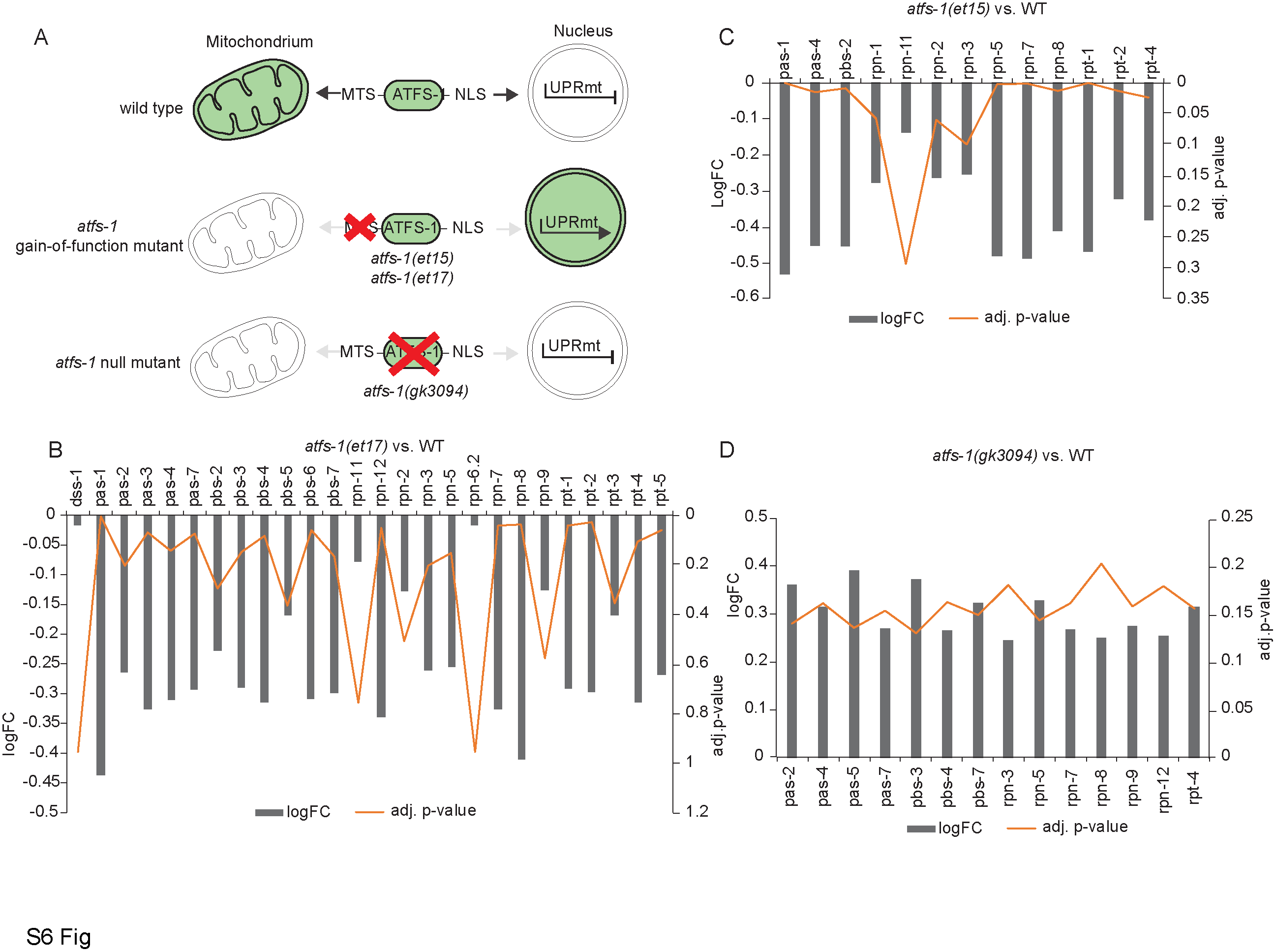

Supplement: S6 Fig — The data are from Wu and colleagues [47]. (A) Schema of the localization of ATFS-1 depending on atfs-1 mutation. (B, C) The expression of proteasome subunits tends to decrease in the atfs-1 gain-of-function mutant. (D) The expression of proteasome subunits tends to increase in the atfs-1 deletion mutant. Underlying numerical data are presented in S1 Data. ATFS-1, activating transcription factor associated with stress 1; EV, empty vector; MTS, mitochondrial targeting sequence; NLS, nuclear localization signal; UPRmt, mitochondrial unfolded protein response; WT, wild type. (TIF) [file pbio.3001302.s006.tif]

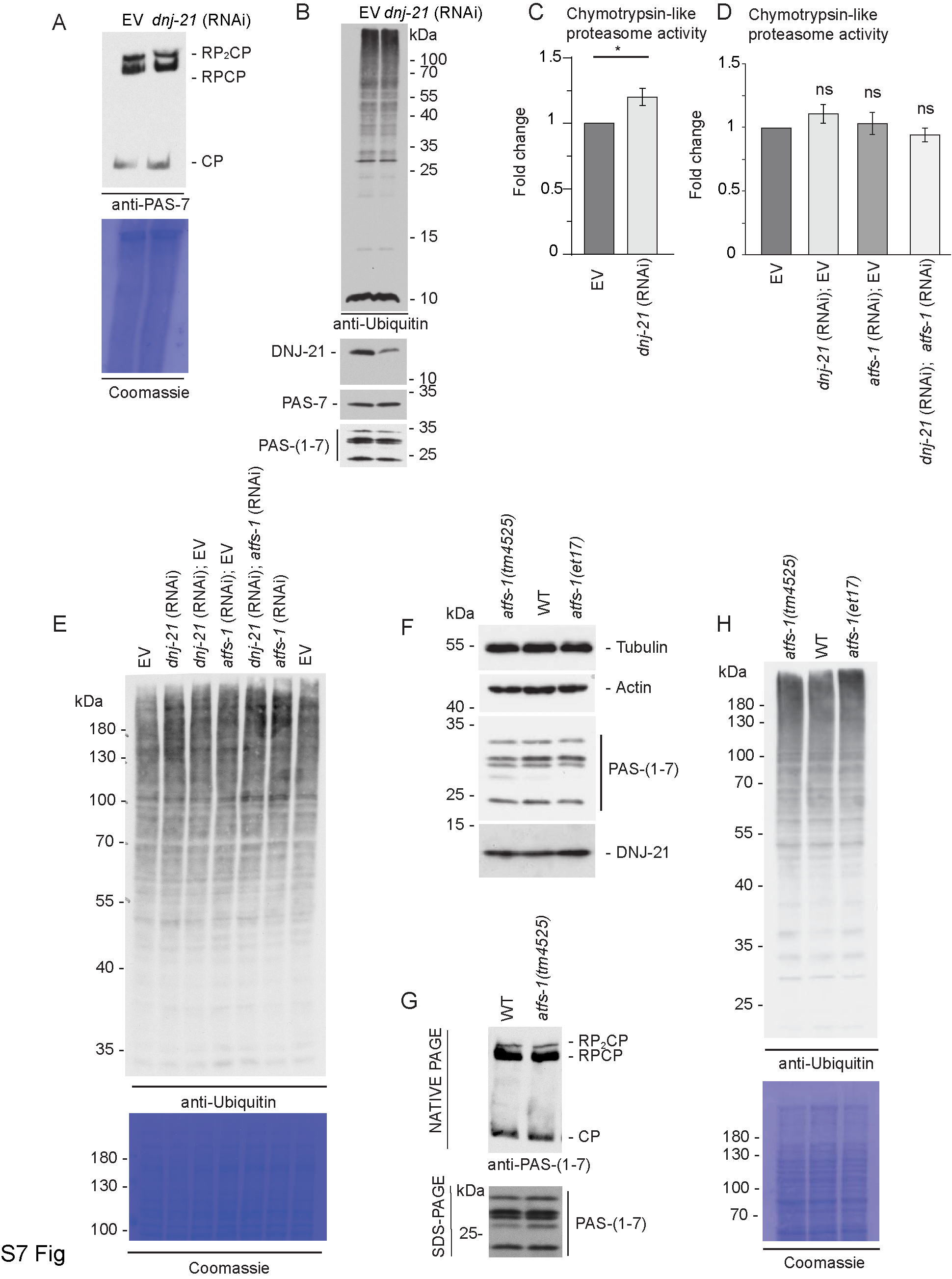

Supplement: S7 Fig — (B, E, F, and H) Total worm lysates were separated by SDS-PAGE and analyzed by western blot using ubiquitin or specific antibodies. (A) Total worm lysates were separated by native PAGE and analyzed by western blot. Equal loading was controlled by Coomassie staining. (B, E) Western blot analysis was repeated in at least 3 biological replicates. (C) Chymotrypsin-like proteasome activity measurement in the fraction of isolated mitochondria. The data are expressed as mean ± SEM. n = 7. *p = 0.02. (D) Synchronized worms were cultured in liquid medium that contained RNAi bacteria. Chymotrypsin-like activity was measured with fluorogenic peptides. The data are expressed as mean ± SD. n = 3. (F–H) Synchronized populations of WT worms and mutants of atfs-1 were cultured on plates that were seeded with HT115(DE3) bacteria. Total worm extracts were separated by SDS-PAGE or native PAGE (G) and analyzed by western blot using specific antibodies. Western blot analysis was repeated in 2 biological replicates. Underlying numerical data are presented in S1 Data. ATFS-1, activating transcription factor associated with stress 1; EV, empty vector; ns, not significant; RNAi, RNA interference; WT, wild type. (TIF) [file pbio.3001302.s007.tif]

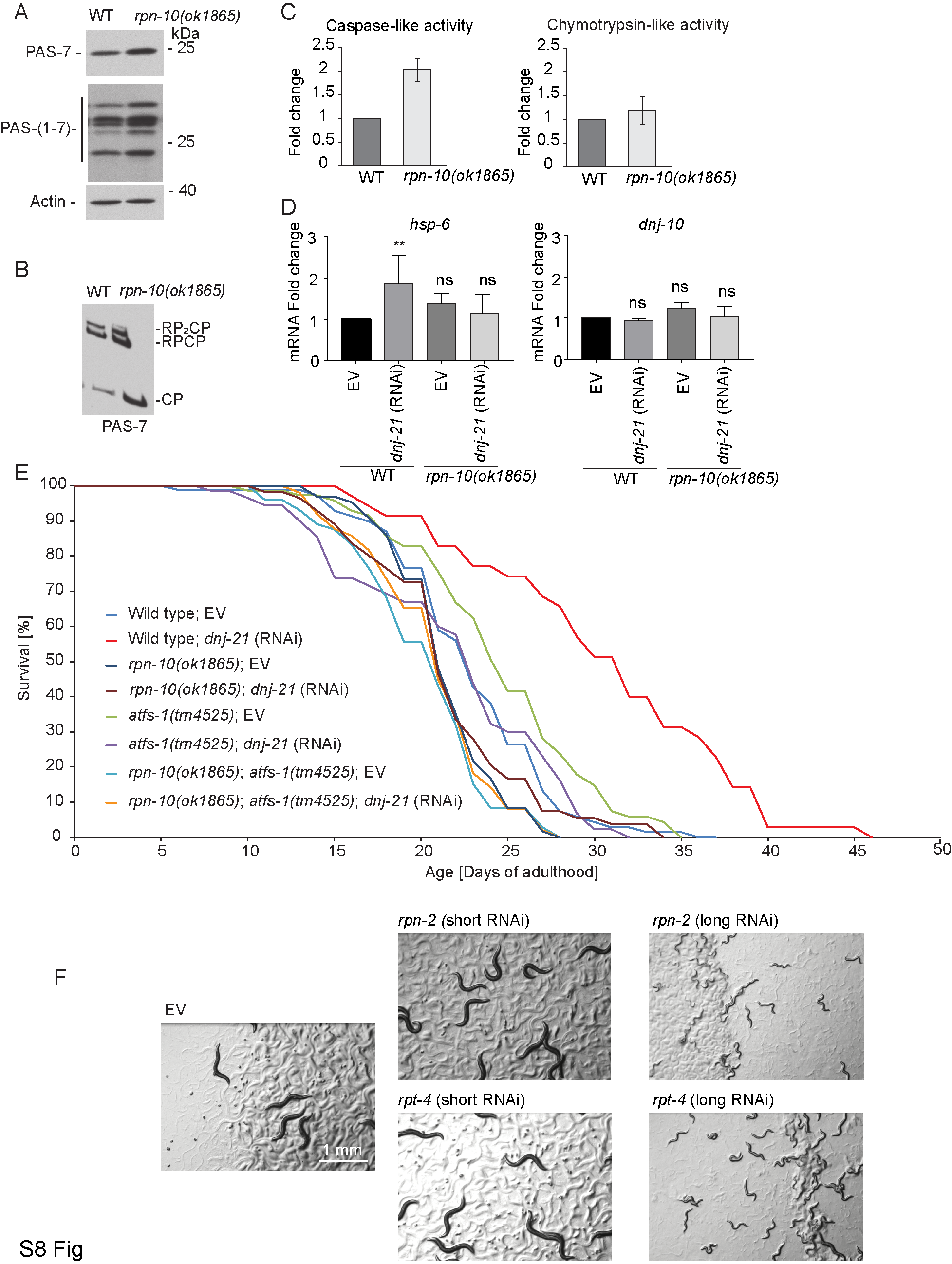

Supplement: S8 Fig — (A, B) Total worm lysates were separated by SDS-PAGE or native PAGE and analyzed by western blot. Western blot analysis was repeated in at least 3 biological replicates. (C) Synchronized worms were fed empty HT115(DE3) bacteria. Proteasomal activity was measured with fluorogenic peptides. The data are expressed as mean ± SD. n = 2. (D) WT and rpn-10 deletion mutant worms were fed bacteria that contained indicated RNAi from the L1 larval stage until young adulthood. RT-qPCR was performed to quantify levels of the indicated mRNAs. **p < 0.01. Kruskal–Wallis test was used for statistical analysis. The qPCR analysis was repeated in 3 biological replicates. (E) Worms were kept on RNAi plates throughout the experiment. Survival curves upon the depletion of DNJ-21 depending on RPN-10 and ATFS-1 function are shown. Life span values are presented in S2 Table. (F) Representative images of worms according to short and long RNAi treatment schema (see also Fig 7C). Images were taken at the same magnification as worms that were continuously kept on control plates (EV) until adulthood. Underlying numerical data are presented in S1 Data. ATFS-1, activating transcription factor associated with stress 1; EV, empty vector; ns, not significant; RNAi, RNA interference; RT-qPCR, quantitative real-time PCR; WT, wild type. (TIF) [file pbio.3001302.s008.tif]
