# Supplementary material for: Proteasome activity contributes to pro-survival response upon mild mitochondrial stress in Caenorhabditis elegans
Source: PLoS Biol. 2021 Jul 12;19(7):e3001302. doi: 10.1371/journal.pbio.3001302 (PMC8274918; doi:10.1371/journal.pbio.3001302)
Supplement: S2 Table — Related to Figs 2A–2C, 5D, 5E, 7B, 7E and 7F and S2B, S2E, S2F, S5B, S5D and S8E Figs. (PDF) [file pbio.3001302.s010.pdf]

S2 Table. Adult lifespan analysis. Related to Figs 2A-C, 5D, E, 7B, E, F, S2B, E, F, S5B, D, S8E.

Strain/ treatment	Mean lifespan ± SE (Days)	75th percentile (Days)	Number of worms observed/ Total initial worms (number of independent experiments)	Change in mean lifespan in % compared to control	Adj. p-value against control	Adj. p-value against specific groups
**Lifespan dependence on components of the mitochondrial import machinery**						
RNAi in N2 background						
EV*	20.99±0.18	24	420/540 (4)	NA	NA	
dnj-21	28.1±0.24	31	342/599(4)	+33	0	
timm-22	21.97±0.28	25	199/207 (2)	+5	0.004	
timm-23	25.7±0.30	28	126/202 (2)	+22	0	
RNAi in *rrf-3(pk1426)* background						
EV*	15.88±0.22	18	211/ 278 (2)	NA	NA	
ZK616.2	19.24±0.23	21	80/156 (1)	+21	0	1 #1
ZK616.3	19.33±0.3	21	83/157 (1)	+22	0	1 #2
ZK616.2; ZK616.3	19.32±0.32	21	107/160 (1)	+22	0	
F11C1.1	16.85±0.30	20	128/146 (1)	+6	0.000031	1 #3
F42H10.2	15.26±0.26	17	107/118 (1)	-4	1	0.0044 #4
F11C1.1; F42H10.2	16.12±0.39	20	111/128 (1)	+2	0.0044	
**Lifespan dependence on *atfs-1* (UPRmt activation)** [in N2 background]						
EV	26.41±0.22	29	401/600 (4)	NA	NA	
dnj-21; EV	31.51±0.40	36	286/450 (3)	+19	0	
atfs-1; EV	23.49±0.23	26	284/450 (3)	-11	0	
dnj-21; atfs-1	24.49±0.20	26	238/450 (3)	-7	0	0.0153 #5
**Lifespan dependence on proteasome** [in N2 background or as indicated]						
EV	21.16±0.48	24	79/151 (1)	NA	NA	
dnj-21; EV	28.79±0.76	34	58/150 (1)	+36	0	
rpn-2; EV	14.16±0.13	15	108/151 (1)	-33	0	
rpn-2; dnj-21	16.72±0.20	18	109/150 (1)	-21	0	0 #6
rpt-4; EV	15.18±0.15	16	117/142 (1)	-28	0	
rpt-4; dnj-21	16.61±0.18	18	165/172 (1)	-22	0	1.1E-07 #7
N2; EV	26.41±0.37	29	401/600 (4)	NA	NA	
N2; *dnj-21*	32.07±0.59	36	203/450 (3)	+21	0	
rpn-10(ok1865); EV	24.36±0.25	27	297/450 (3)	-8	1.5E-08	
rpn-10(ok1865); dnj-21	24.45±0.46	27	192/450 (3)	-7	0.0003	1 #8
**Lifespan dependence on UPRmt and functional proteasome**						
RNAi in N2 background						
EV	22.91±0.57	27	68/86 (1)	NA		
dnj-21	30.69±1.26	37	35/88 (1)	+34	0	

RNAi in *rpn-10(ok1865)* background						
EV	21.53±0.38	23	62/83 (1)	NA		
dnj-21	21.6±0.64	24	54/88 (1)	+0,3	1	
RNAi in *atfs-1(tm4525)* background						
EV	24.69±0.63	28	68/88 (1)	NA		
dnj-21	21.84±0.9	27	44/85 (1)	-11	0.2357	
RNAi in *atfs-1(et17)* background						
EV	21.95±0.98	27	30/85 (1)	NA		
dnj-21	23.33±0.76	25	18/90 (1)	+6	1	
RNAi in *rpn-10(ok1865); atfs-1(tm4525)* background						
EV	20.19±0.47	23	72/81 (1)			
dnj-21	20.8±0.52	23	49/83 (1)	+3	1	

*Cumulative values are given because single biological repetitions were comparable to each other

#1 vs. ZK616.2; ZK616.3

#2 vs. ZK616.2; ZK616.3

#3 vs. F11C1.1; F42H10.2

#4 vs. F11C1.1; F42H10.2

#5 vs. *atfs-1*; EV

#6 vs. *rpn-2*; EV

#7 vs. *rpt-4*; EV

#8 vs. *rpn-10(ok1865)*;EV

NA, not applicable
