# Supplementary material for: Proteasome activity contributes to pro-survival response upon mild mitochondrial stress in Caenorhabditis elegans
Source: PLoS Biol. 2021 Jul 12;19(7):e3001302. doi: 10.1371/journal.pbio.3001302 (PMC8274918; doi:10.1371/journal.pbio.3001302)
Supplement: S3 Table — Listed are proteins identified by MS (see S1 Table) upon dnj-21 RNAi, which were shown previously in the literature to be up-regulated on the transcriptional level upon induction of the UPRmt. Related to S1 Table. MS, mass spectrometry; RNAi, RNA interference; UPRmt, mitochondrial unfolded protein response. (PDF) [file pbio.3001302.s011.pdf]

S3 Table. Targets of the UPRmt. Listed are proteins identified by mass spectrometry (see S1 Table) upon *dnj-21* RNAi, which were shown previously in the literature to be upregulated on the transcriptional level upon induction of the UPRmt. Related to S1 Table.

	**Protein ID`s**	**Gene name**	**Log2 Fold change *dnj-21* RNAi vs. EV**	**p-value**	**Previously identified by**
Mitochondrial protein homeostasis	Q8TA83	*dnj-10*	0.064	0.457	[25, 26, 55]
	P11141	*hsp-6*	0.478	0.003	[26, 55]
	V6CLG8	*hsp-60*	0.436	0.014	[55]
	P50140;G8JYF5	*hsp-60*	0.260	0.034	[55]
	G5EDB6;G5EGR7	*ppgn-1*	0.462	0.141	[26, 55]
	P54813	*ymel-1*	0.496	0.019	[25, 55]
Mitochondrial protein import	Q19766	*tomm-20*	0.178	0.335	[26, 55]
	Q9XVQ2	*timm-23*	0.041	0.781	[29, 55]
	O44477	*timm-17B.1*	0.839	0.002	[25, 26, 55]
Innate imunity	O62416	*lys-2*	0.618	0.078	[25, 86]
Metabolism	H9G2T4;Q21032;H9G2T3	*idh-1*	-0.003	0.964	[47]
	O17643	*idh-2*	-0.182	0.187	[25, 55]
	B0M0N9;B0M0P0;B6VQ90;H2L2H2;H2L2H3;U4PRW7	*glna-1*	0.702	0.001	[26, 55]
	P48376	*clk-1*	0.629	0.449	[25, 26, 55]
	P54216	*aldo-1*	0.895	0.046	[47]

References

86. Pellegrino MW, Nargund AM, Kirienko NV, Gillis R, Fiorese CJ, Haynes CM. Mitochondrial UPR-regulated innate immunity provides resistance to pathogen infection. Nature. 2014;516(7531):414-7. Epub 2014/10/03. https://doi.org/10.1038/nature13818.
