# Supplementary material for: Proteasome activity contributes to pro-survival response upon mild mitochondrial stress in Caenorhabditis elegans
Source: PLoS Biol. 2021 Jul 12;19(7):e3001302. doi: 10.1371/journal.pbio.3001302 (PMC8274918; doi:10.1371/journal.pbio.3001302)
Supplement: S4 Table — (PDF) [file pbio.3001302.s012.pdf]

S4 Table. *C. elegans* strains used in this study

Strain	Description	Reference
N2	Wild type	[71]
SJ4100 (zcIs13[*hsp-6::gfp*])	reporter strain for UPRmt	*Caenorhabditis* Genetics Center (Minneapolis, MN)
ACH2 (*rpn-10(ok1865*))	VC1369 *rpn-10(ok1865)* backcrossed 4x with N2	This study
ACH5 (xzEx3[unc-54p::UbG76V::Dendra2])	AGD1033 *glp-1(e2141)*; xzEx3[unc-54p::UbG76V::Dendra2] crossed with N2 to remove *glp-1(e2141)*	This study
ACH89	wacIs14[myo-3p::tomm-20 1-50aa::attB5::mGFP::unc-54-3'UTR, unc-119(+)]	This study
NL2099 (*rrf-3*(*pk1426*))	RNAi sensitive strain	*Caenorhabditis* Genetics Center (Minneapolis, MN)
QC117 (*atfs-1*(*et17*))	*atfs-1(et17)* V; *atfs-1* Gain-Of-Function mutant	*Caenorhabditis* Genetics Center (Minneapolis, MN)
ACH11 (*atfs-1(tm4525)*)	FX04525 (*atfs-1 (tm4525)*) backcrossed 4 times with N2	This study
ACH200 (*rpn-10(ok1865); atfs-1(tm4525*))	ACH2 (*rpn-10(ok1865*)) crossed with ACH11 (*atfs-1(tm4525)*)	This study
