# Supplementary material for: Proteasome activity contributes to pro-survival response upon mild mitochondrial stress in Caenorhabditis elegans
Source: PLoS Biol. 2021 Jul 12;19(7):e3001302. doi: 10.1371/journal.pbio.3001302 (PMC8274918; doi:10.1371/journal.pbio.3001302)
Supplement: S5 Table — (PDF) [file pbio.3001302.s013.pdf]

S5 Table. Primers used for gene expression analysis

Gene name	Forward primer (5'3')	Reverse primer (3'5')	Reference
hsp-6	GCATCATTATTCTCCTAAAACTTG	GTGCCTGAAAGAAAATTGCATC	[55]
dnj-10	GCGGGCTCATTCATCGATCTGTAC	CAGATTTTTTGTCGACACCCAAAG	[25]
timm-23	CAACTGAAATCTGCTGGAGTAGGAG	GGCATAATGTATTGCGGCTGC	[55]
gpd-2	TGAAATCCAATGGGGAGCCTC	GGAGCAGAGATGATGACCTTCTTG	[25]
aldo-1	GCACAAGATCTCATCTACGACTCC	GAGAGCGTGGTACACATATGAGAG	[87]
acs-2	TGTGAAGGGTGGTGAGAACG	GCGCAGATGTTCTCTCCGTA	[88]
T14G8.3	GCCAGTGGAGCCAAAAGCAAA	CCAAGCGGTTCATAGCCTCTT	[49]
crt-1	ATGACGAGATGGACGGAGAAT	CTGACTTGACCTGCCACAAAT	[49]
hsp-4	GGGGACAATCATTGGTATCG	ACGCAACGTATGATGGAGTG	[48]
hsp-3	GAACCATCGCTGGATTGAAC	CCTCCAAGATCGAAGACGAG	[48]
hsp-16.2	CTTTACCACTATTTCCGTCCA	TAACAATCTCAGAAGACTCAGA	[89]
hsp-70	TGCACCAATCTGGACAATCT	TCCAGCAGTTCCAGGATTTC	[90]
pbs-1	TGTGCCGTTCTGGATCTG	TGTAGAGGAATTGGCGGA	[89]
pas-5	GCATTGGAATCAAGACGAG	GAGTCGGCAATCAAACCT	[89]
rpn-6	GATGGAGATATTAAGGTTGGAC	TCATTATAGAGGCGAACAAGAC	[89]
rpt-6	GGTTCCTGATTCAACATACGA	TCCAAAGAGCAAGACTCCT	[89]
act-1	GTTGCCCAGAGGCTATGTTC	CAAGAGCGGTGATTTCCTTC	[91]
cdc-42	GGTTGCTCCAGCTTCATTC	AACAAGAATGGGGTCTTTGA	[48]

References

87. Pujol C, Bratic-Hench I, Sumakovic M, Hench J, Mourier A, Baumann L, et al. Succinate dehydrogenase upregulation destabilize complex I and limits the lifespan of gas-1 mutant. PLoS One. 2013;8(3):e59493. Epub 2013/04/05. https://doi.org/10.1371/journal.pone.0059493.

88. Bennett CF, Kwon JJ, Chen C, Russell J, Acosta K, Burnaevskiy N, et al. Transaldolase inhibition impairs mitochondrial respiration and induces a starvation-like longevity response in Caenorhabditis elegans. PLoS Genet. 2017;13(3):e1006695. Epub 2017/03/30. https://doi.org/10.1371/journal.pgen.1006695.

89. Chondrogianni N, Georgila K, Kourtis N, Tavernarakis N, Gonos ES. 20S proteasome activation promotes life span extension and resistance to proteotoxicity in Caenorhabditis elegans. FASEB J. 2015;29(2):611-22. Epub 2014/11/15. https://doi.org/10.1096/fj.14-252189.

90. Ooi FK, Prahlad V. Olfactory experience primes the heat shock transcription factor HSF-1 to enhance the expression of molecular chaperones in C. elegans. Sci Signal. 2017;10(501). Epub 2017/10/19. https://doi.org/10.1126/scisignal.aan4893.

91. Hendriks GJ, Gaidatzis D, Aeschimann F, Grosshans H. Extensive oscillatory gene expression during C. elegans larval development. Mol Cell. 2014;53(3):380-92. Epub 2014/01/21. https://doi.org/10.1016/j.molcel.2013.12.013.
