# Supplementary material for: Proteasome activity contributes to pro-survival response upon mild mitochondrial stress in Caenorhabditis elegans
Source: PLoS Biol. 2021 Jul 12;19(7):e3001302. doi: 10.1371/journal.pbio.3001302 (PMC8274918; doi:10.1371/journal.pbio.3001302)
Supplement: S1 Raw Images — (PDF) [file pbio.3001302.s015.pdf]

Figure 1

Fig. 1A

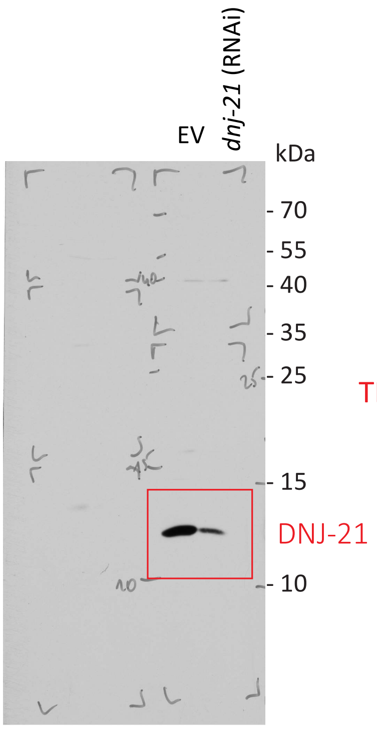

SDS-PAGE

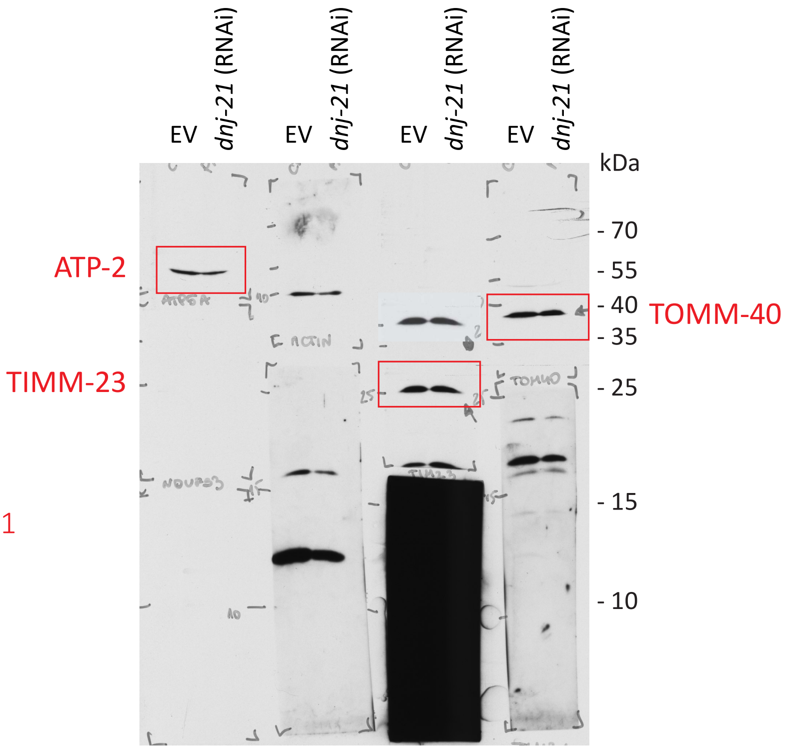

SDS-PAGE

Fig. 1G

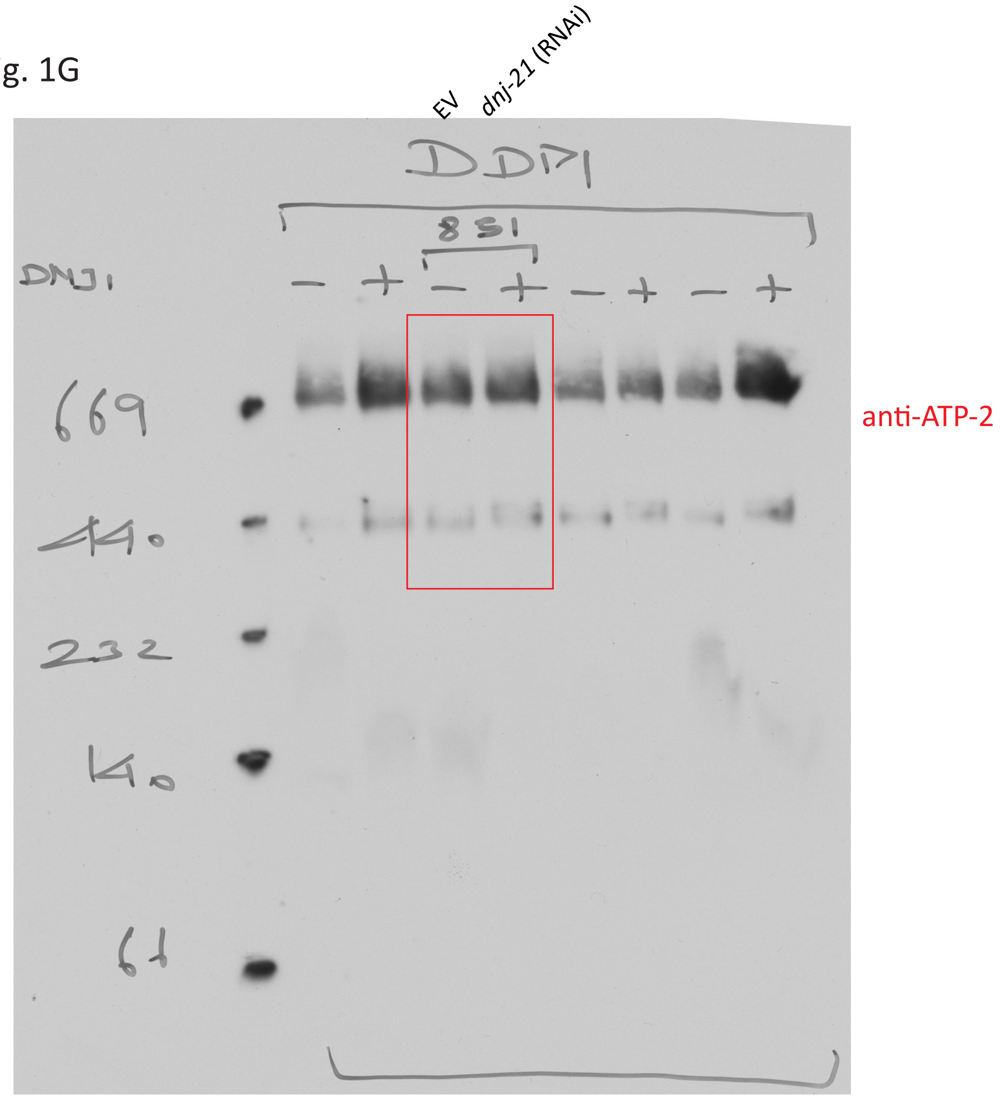

BN-PAGE

After protein transfer membranes were cut and their fragments were incubated with antibodies against indicated proteins.

Figure 3

Fig. 3B

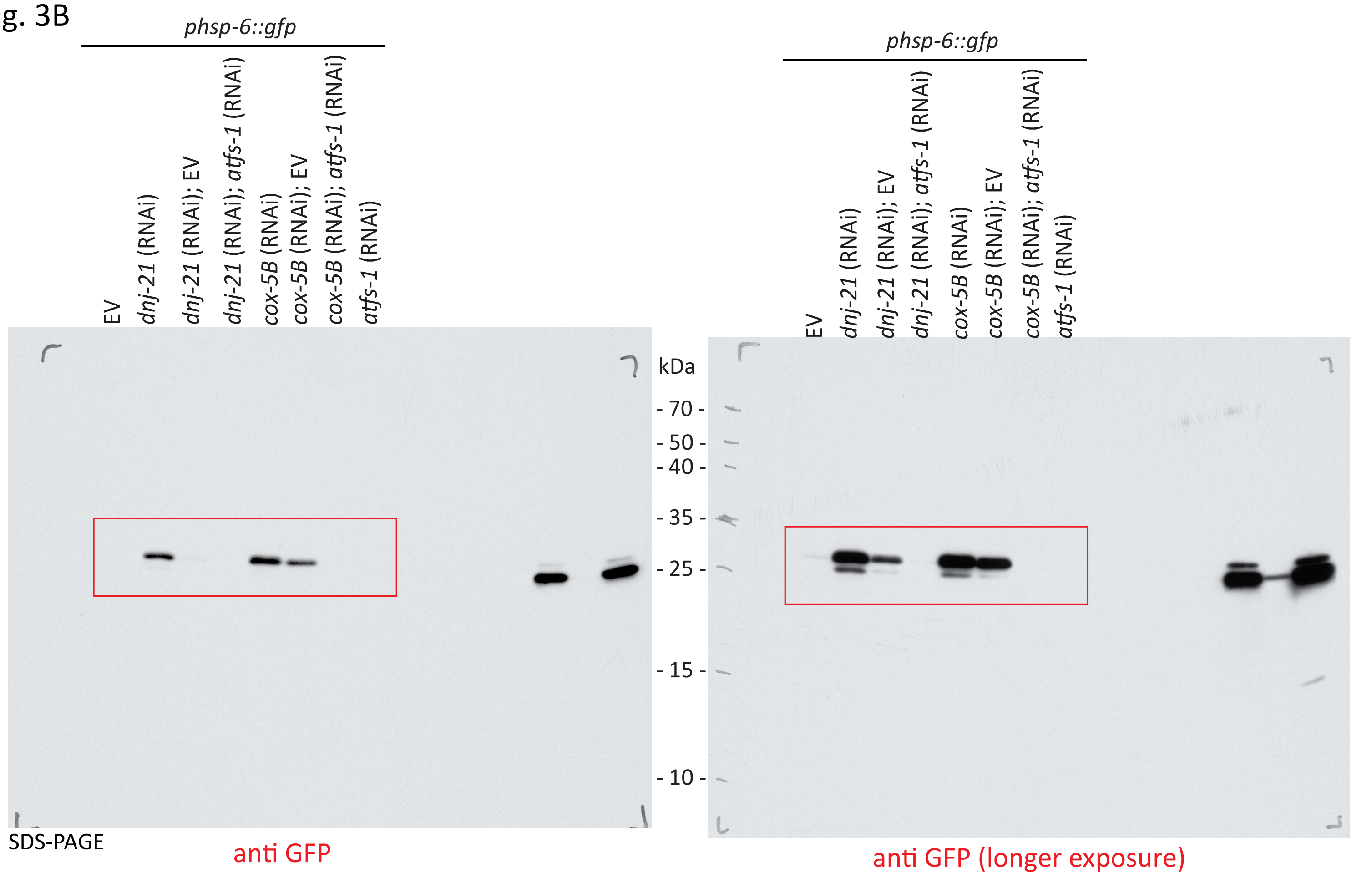

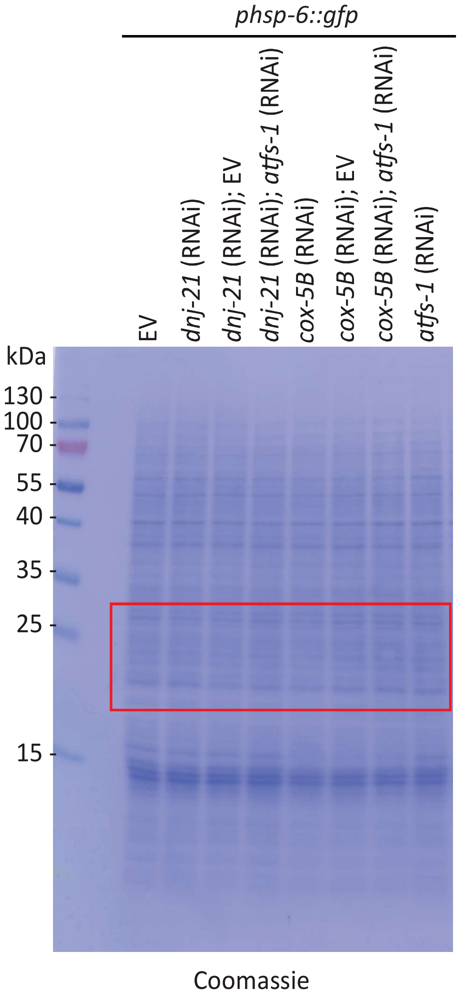

Fig. 3D

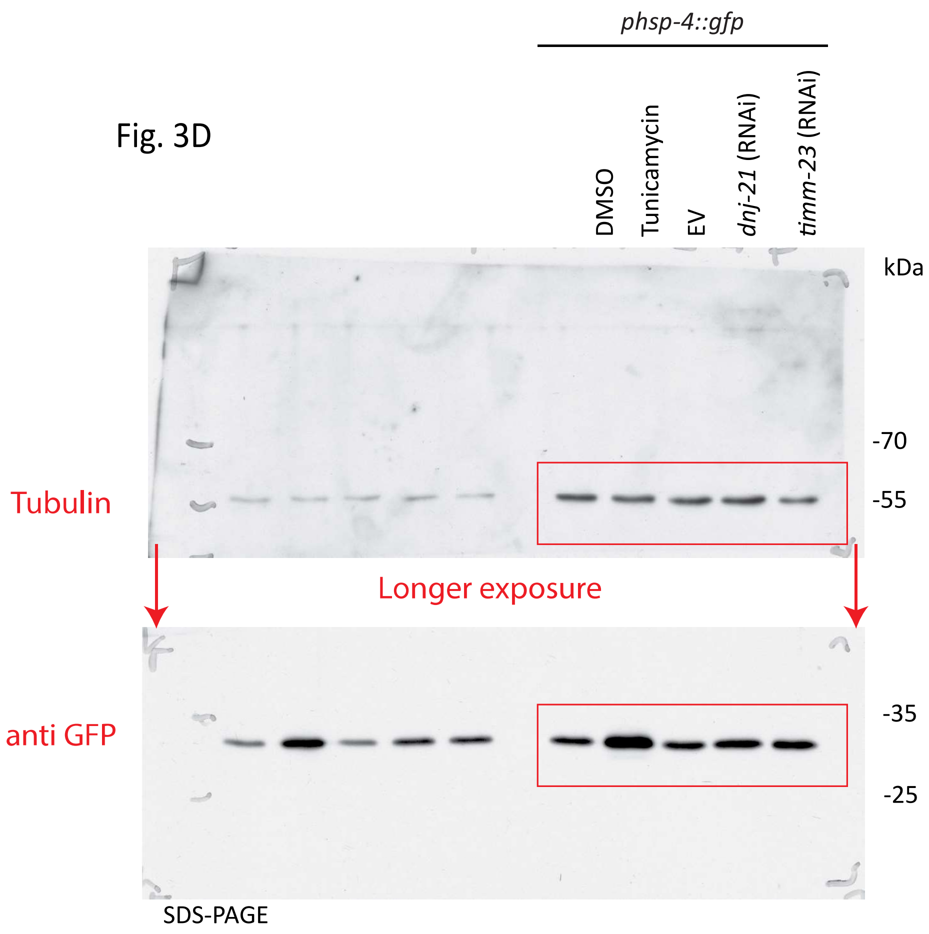

After protein transfer membranes were cut and their fragments were incubated with antibodies against indicated proteins.

Figure 5

Fig. 5A

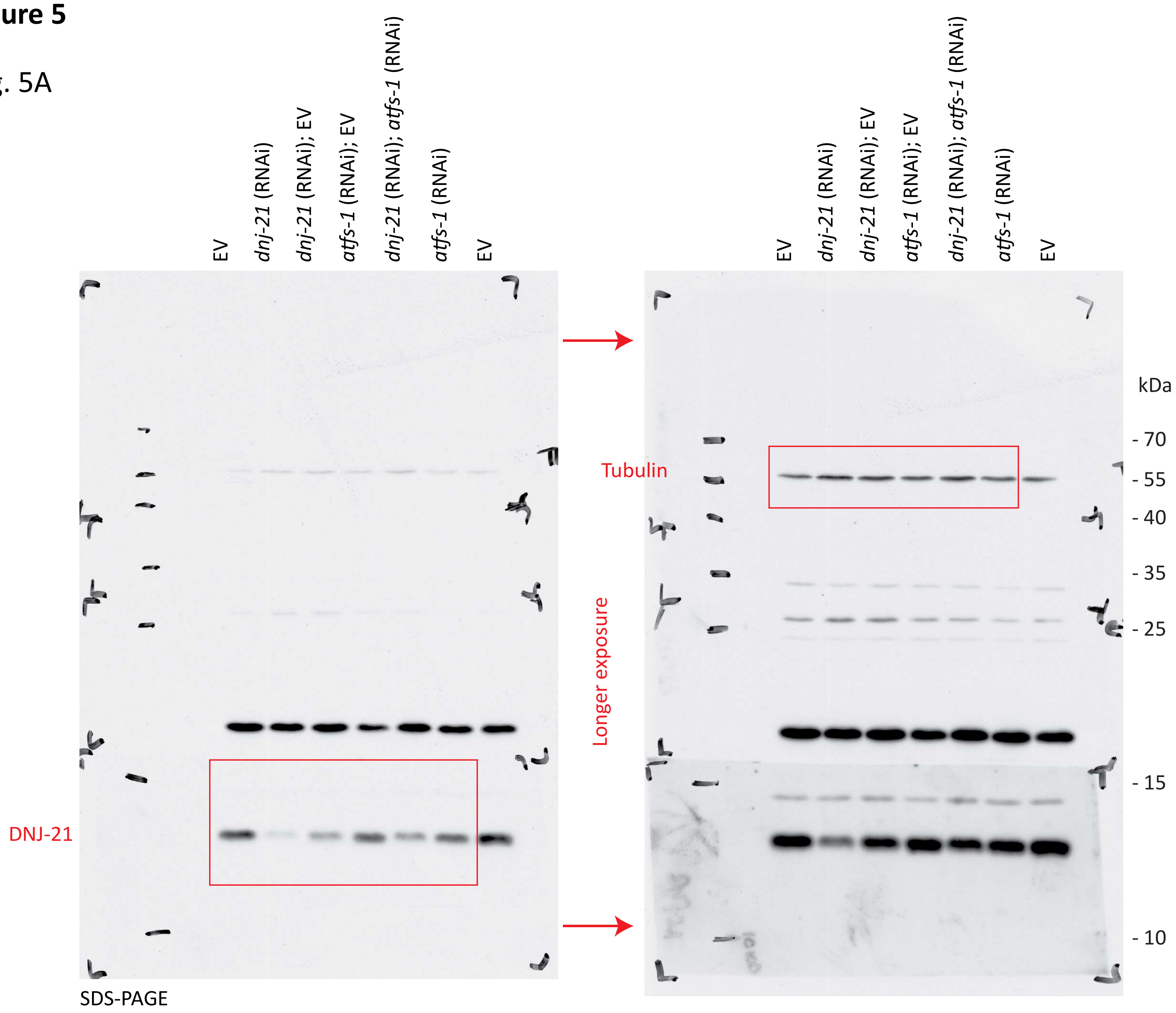

After protein transfer membranes were cut and their fragments were incubated with antibodies against indicated proteins.

Figure 6

Fig. 6B

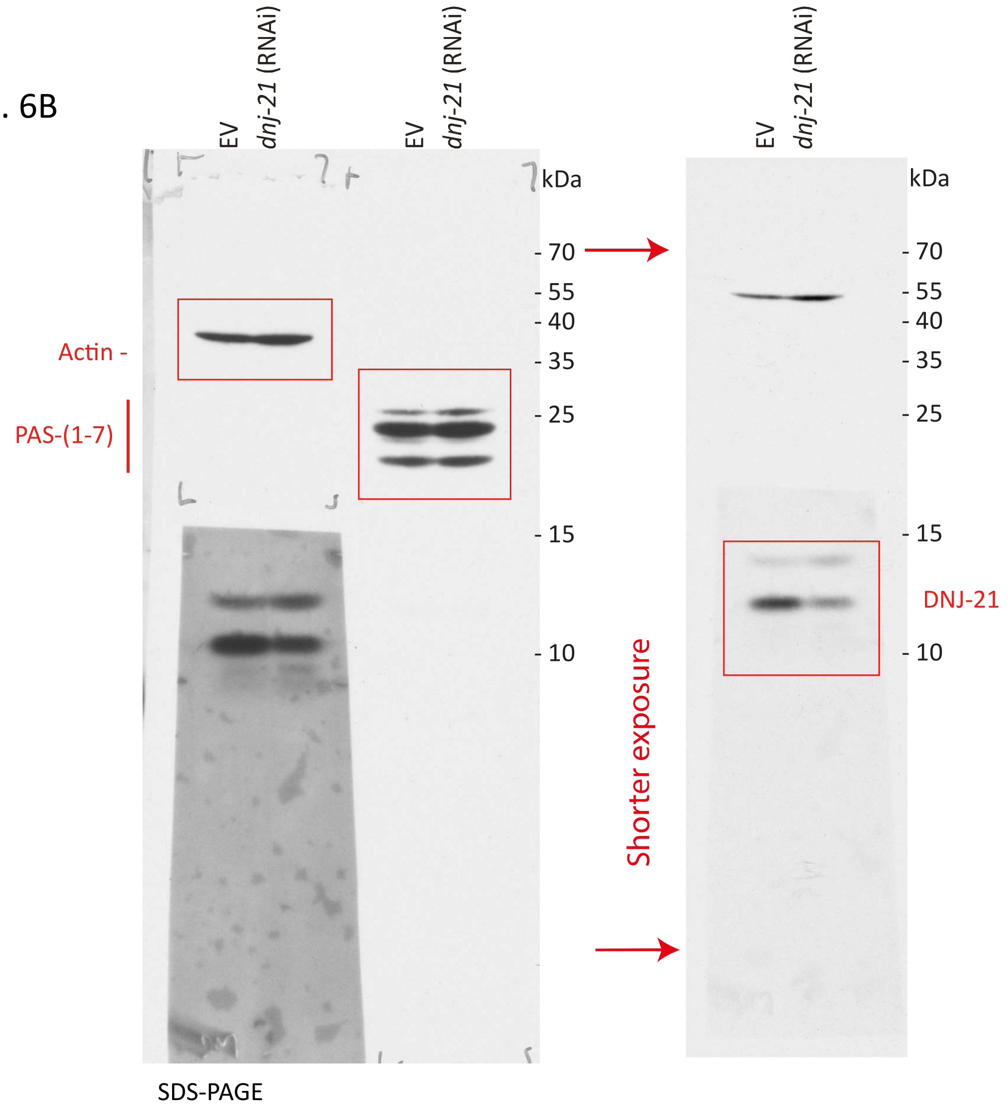

Fig. 6C

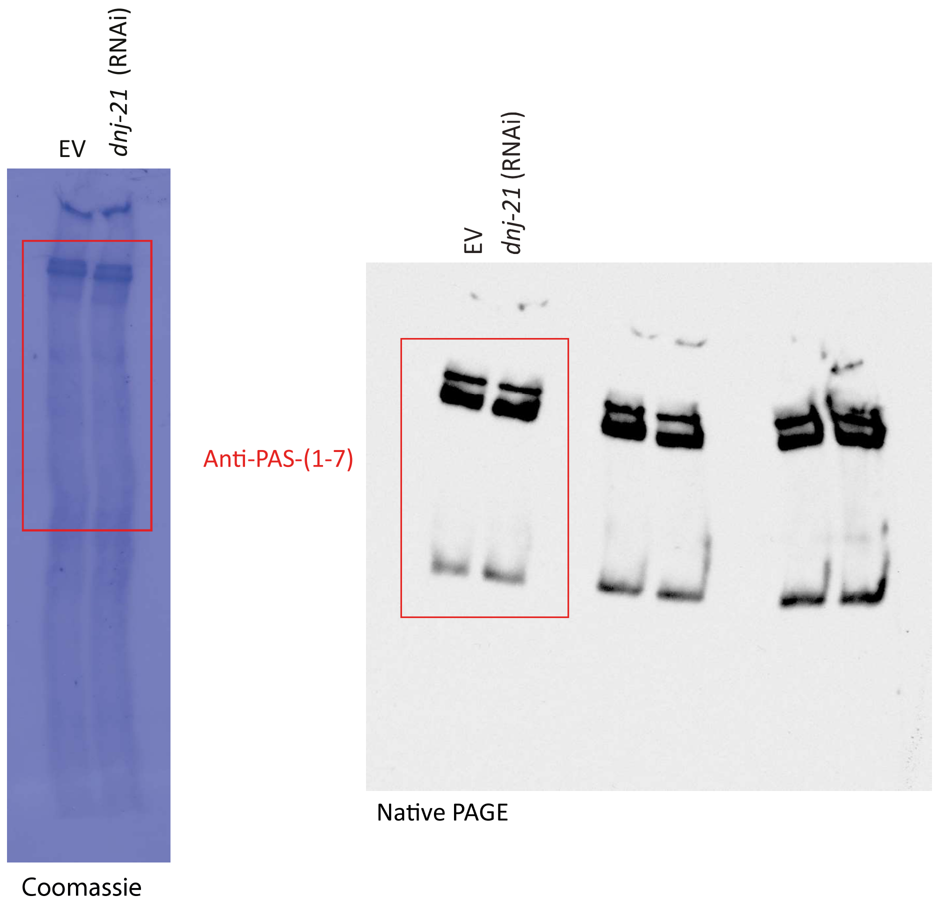

After protein transfer membranes were cut and their fragments were incubated with antibodies against indicated proteins.

Figure 6

Fig. 6E

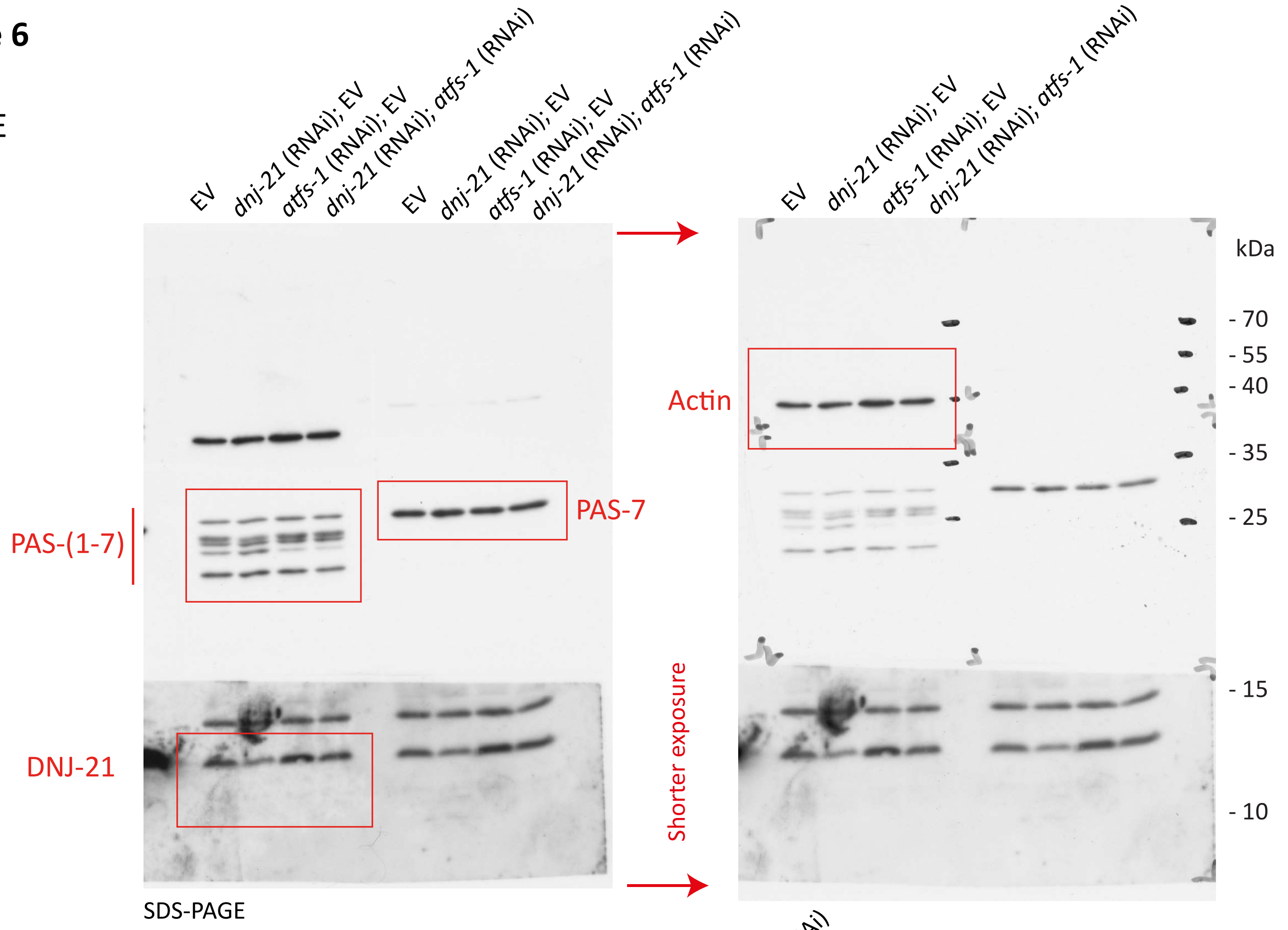

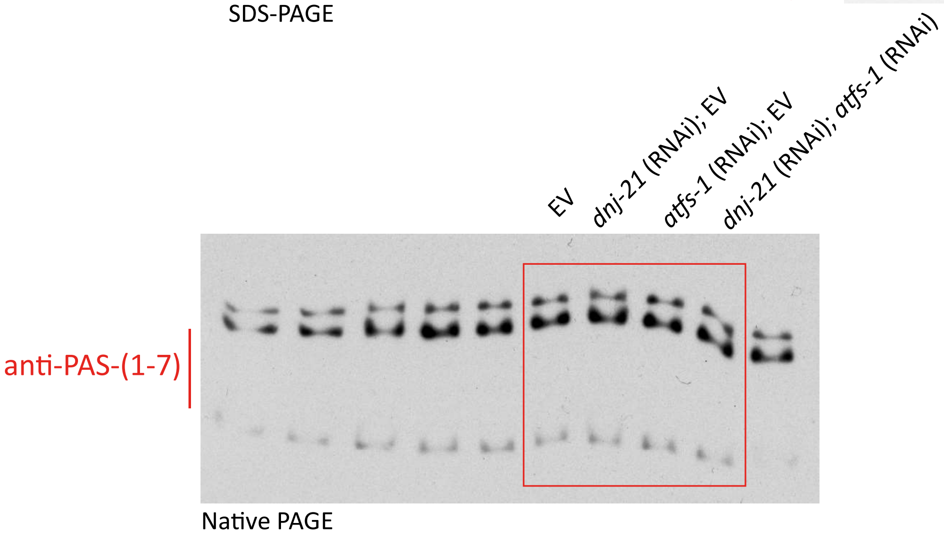

Fig. 6F

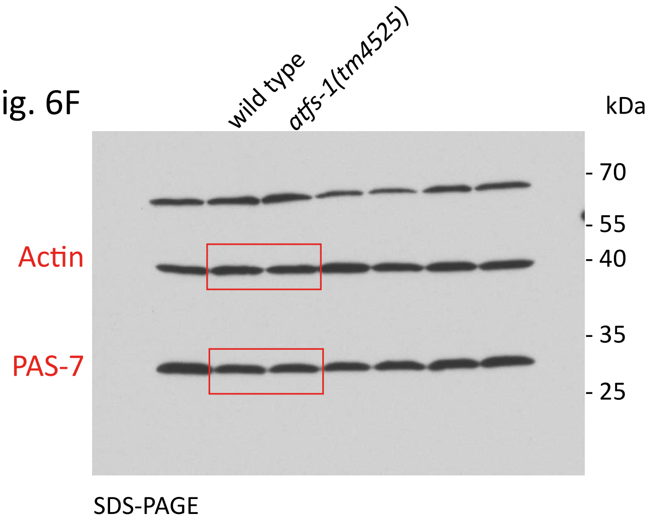

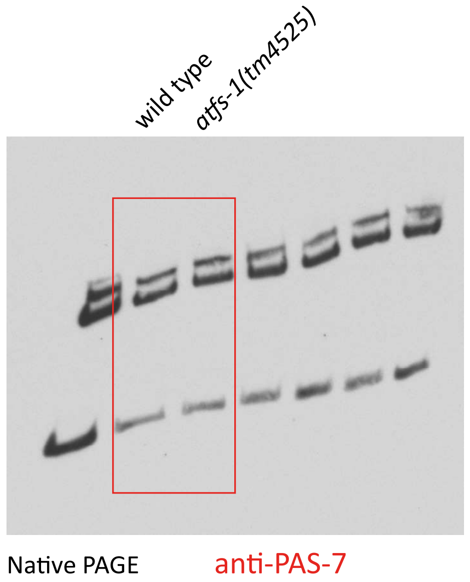

After protein transfer membranes were cut and their fragments were incubated with antibodies against indicated proteins.

Figure 7

Fig. 7A

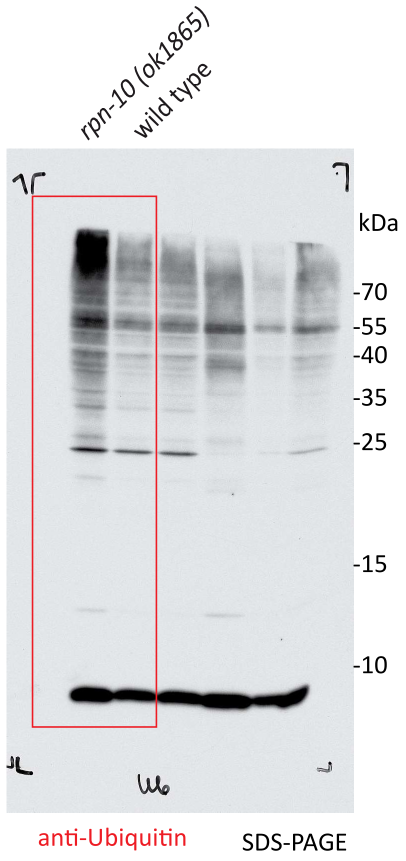

Fig. 7D

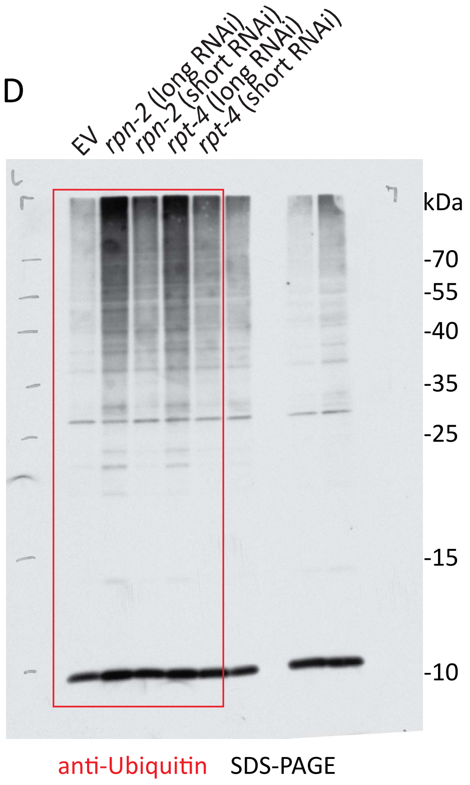

After protein transfer membranes were cut and their fragments were incubated with antibodies against indicated proteins.

Supplementary Figure 1

Supplementary Fig. 1C

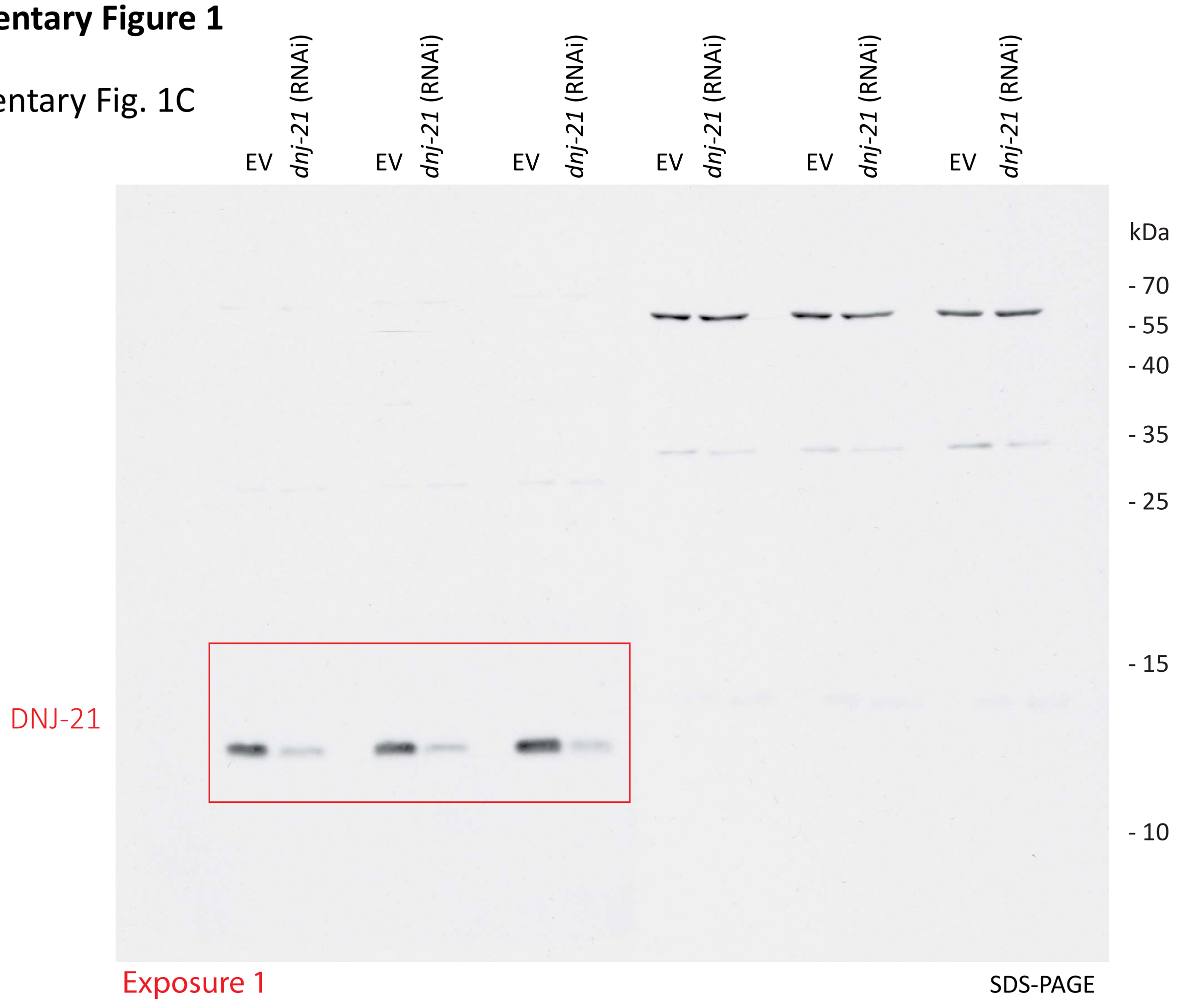

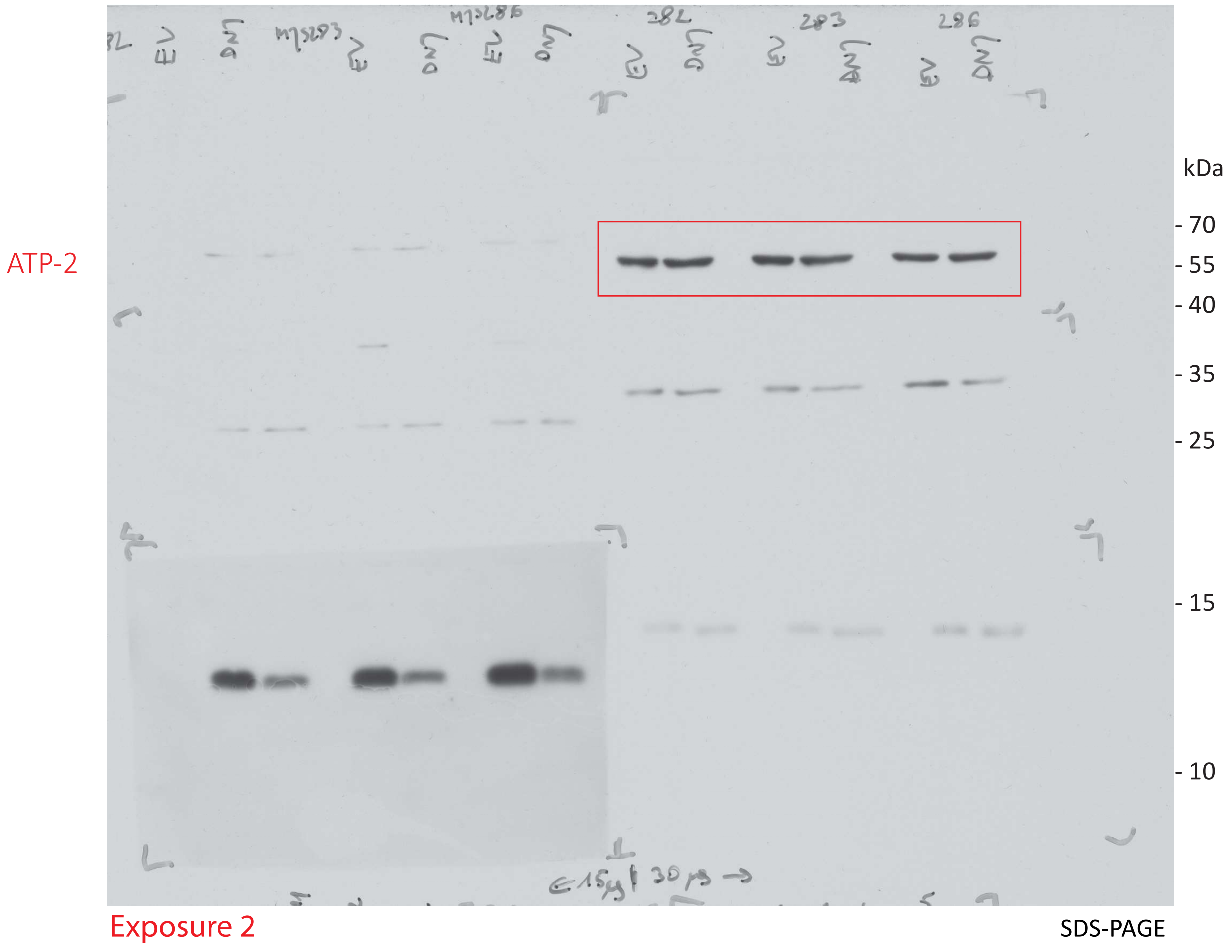

After protein transfer membranes were cut and their fragments were incubated with antibodies against indicated proteins.

Supplementary Figure 1

Supplementary Fig. 1C

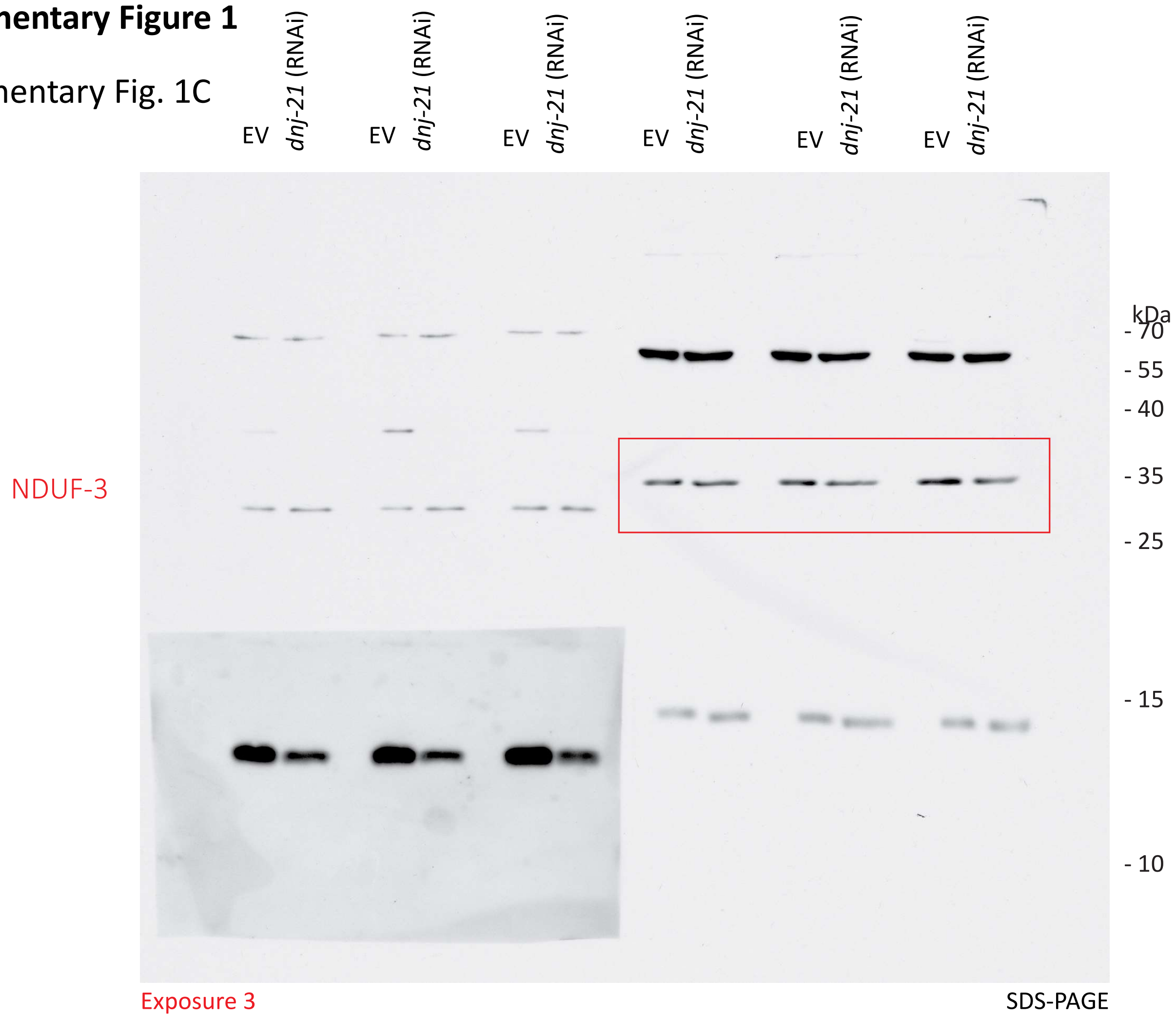

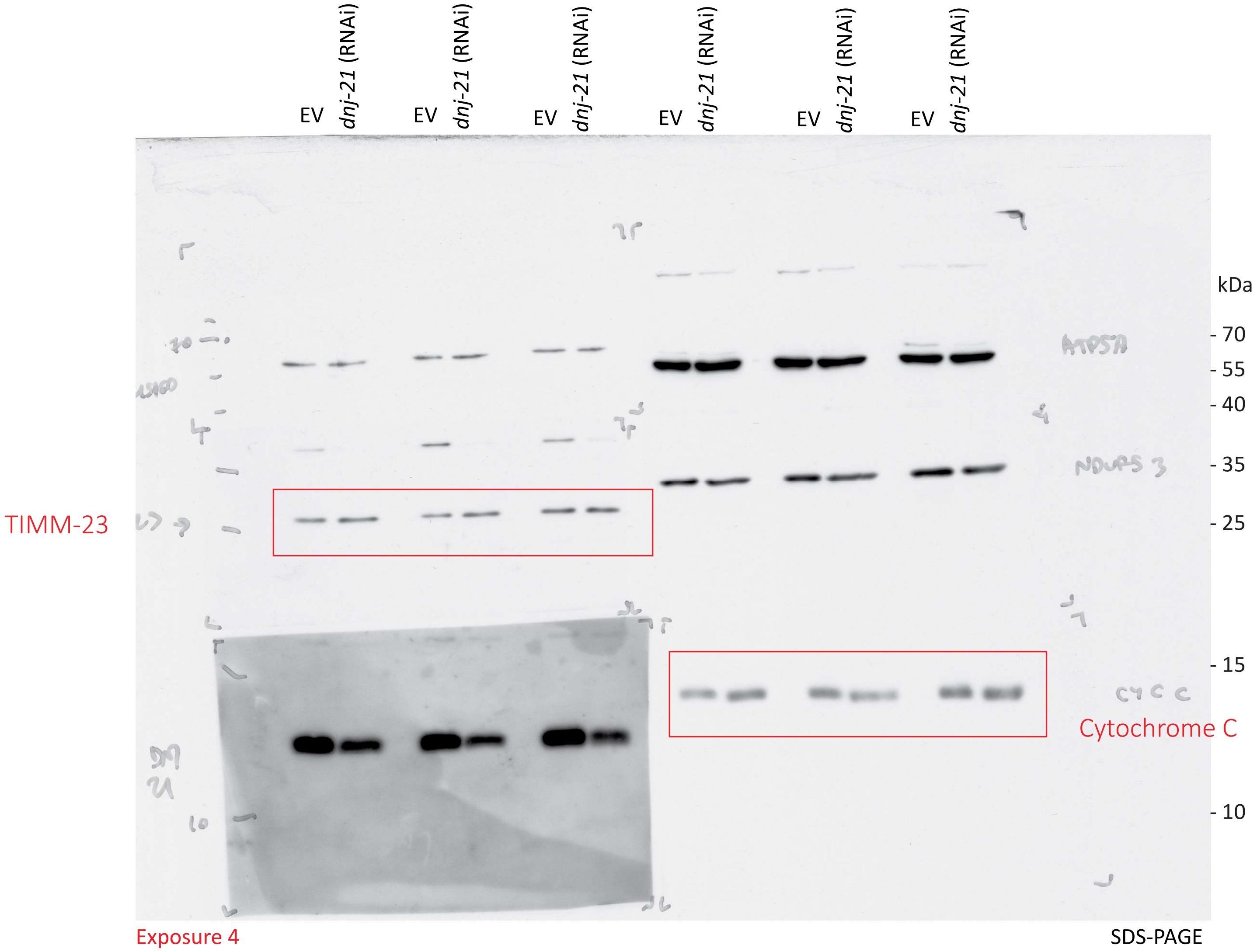

After protein transfer membranes were cut and their fragments were incubated with antibodies against indicated proteins.

Supplementary Figure 2

Supplementary Figure 2 A

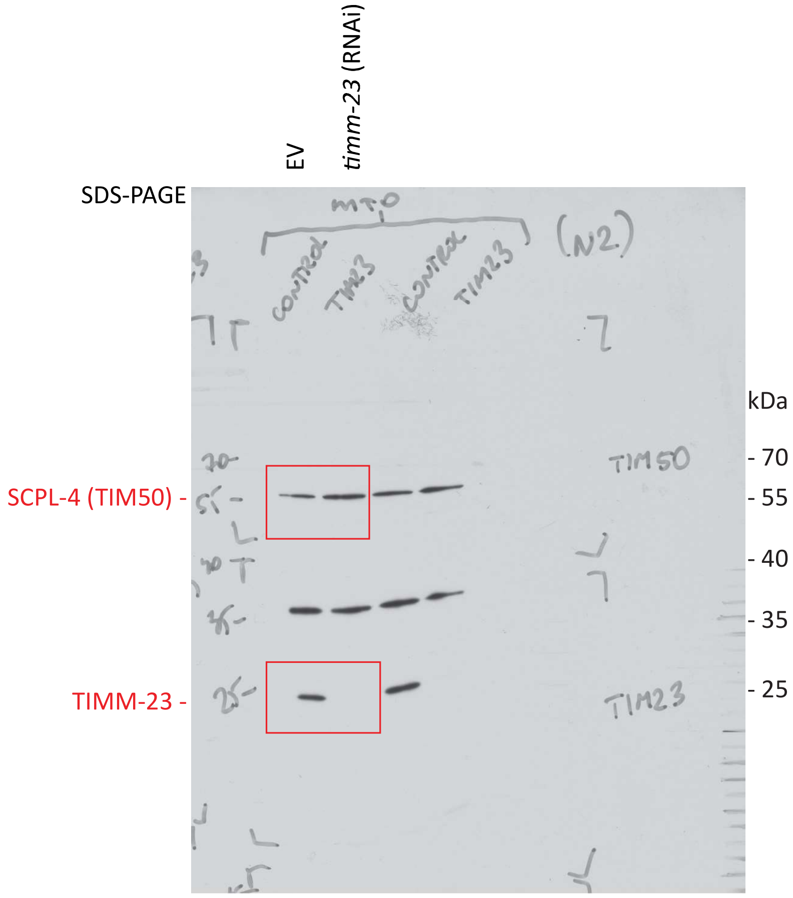

After protein transfer membranes were cut and their fragments were incubated with antibodies against indicated proteins.

Supplementary Figure 7

Supplementary Fig. 7A

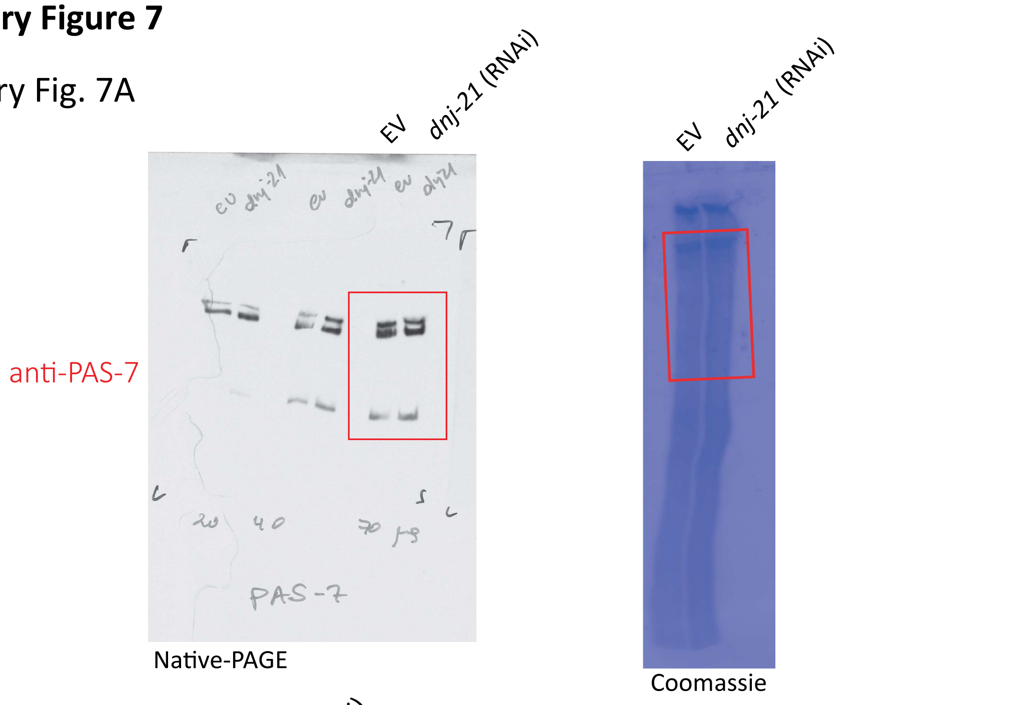

Supplementary Fig. 7B

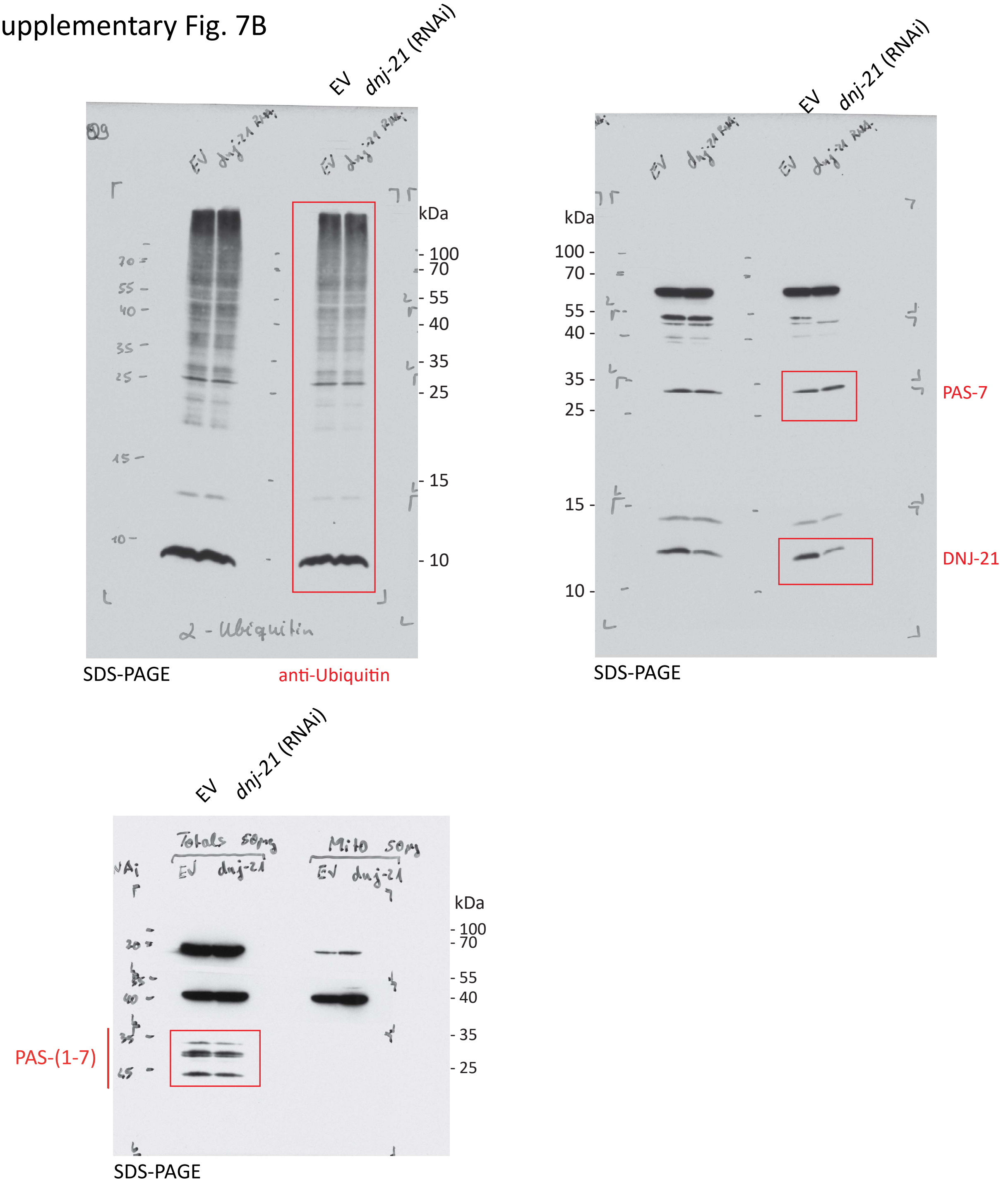

After protein transfer membranes were cut and their fragments were incubated with antibodies against indicated proteins.

Supplementary Figure 7

Supplementary Fig. 7E

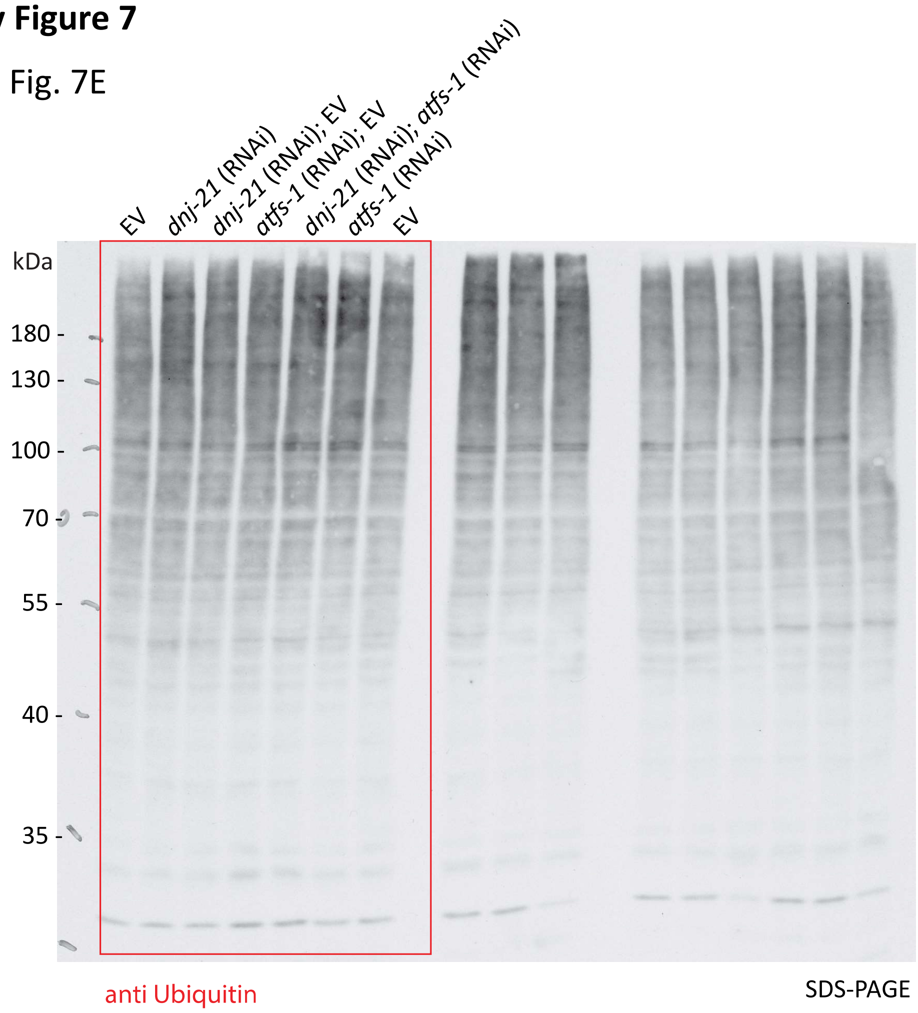

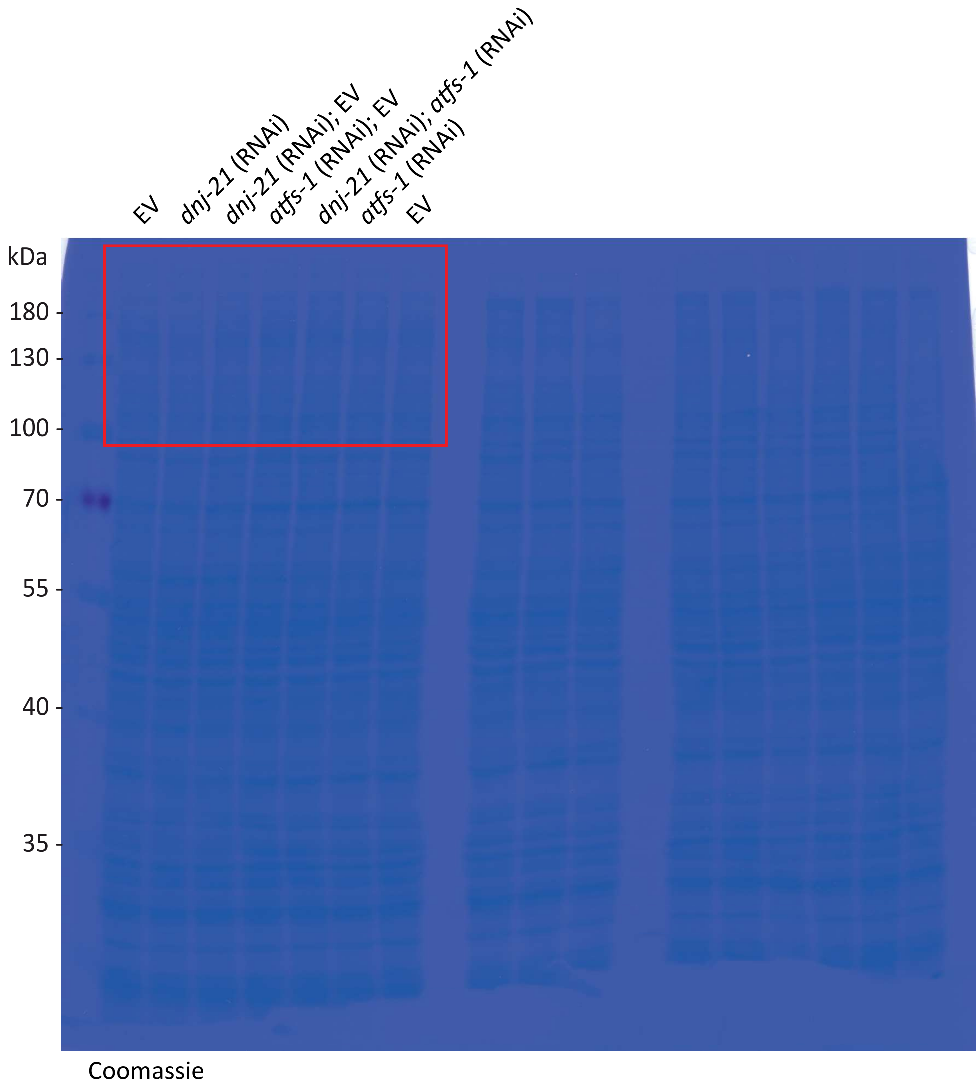

After protein transfer membranes were cut and their fragments were incubated with antibodies against indicated proteins.

Supplementary Figure 7

Supplementary Fig. 7F

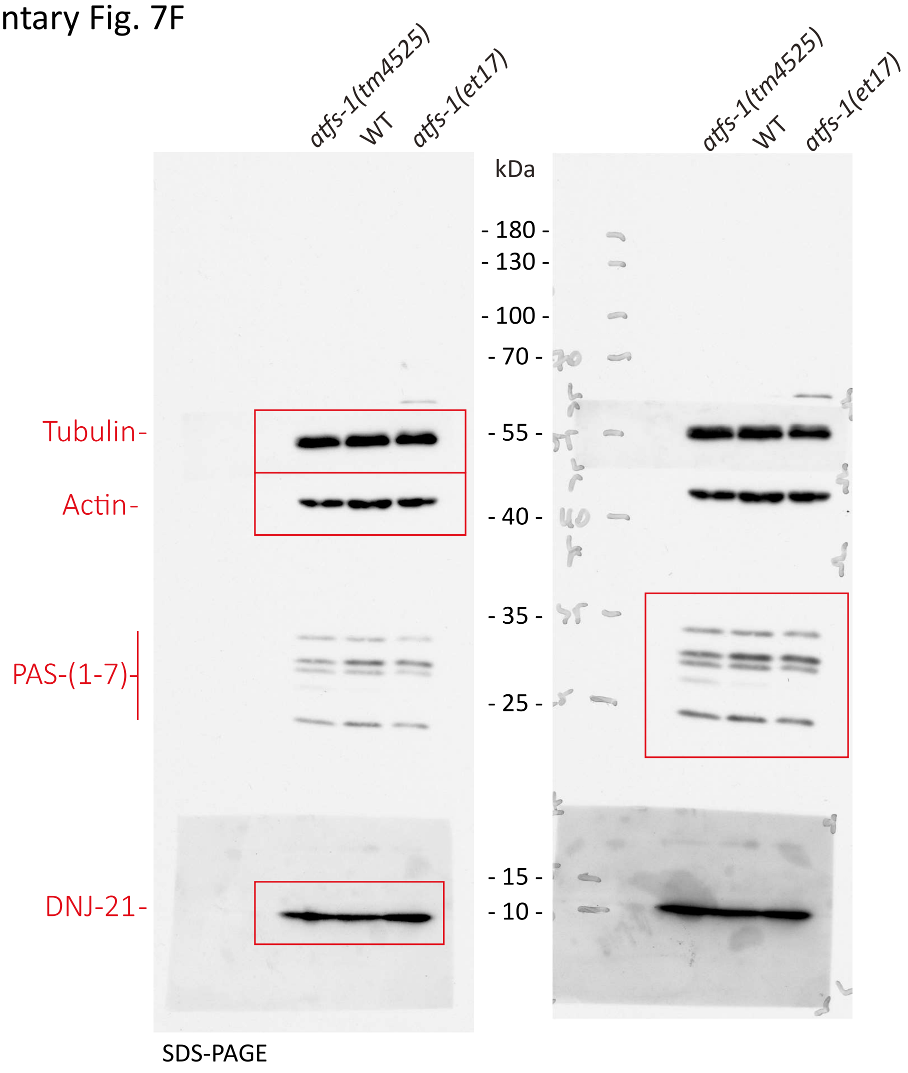

Supplementary Fig. 7G

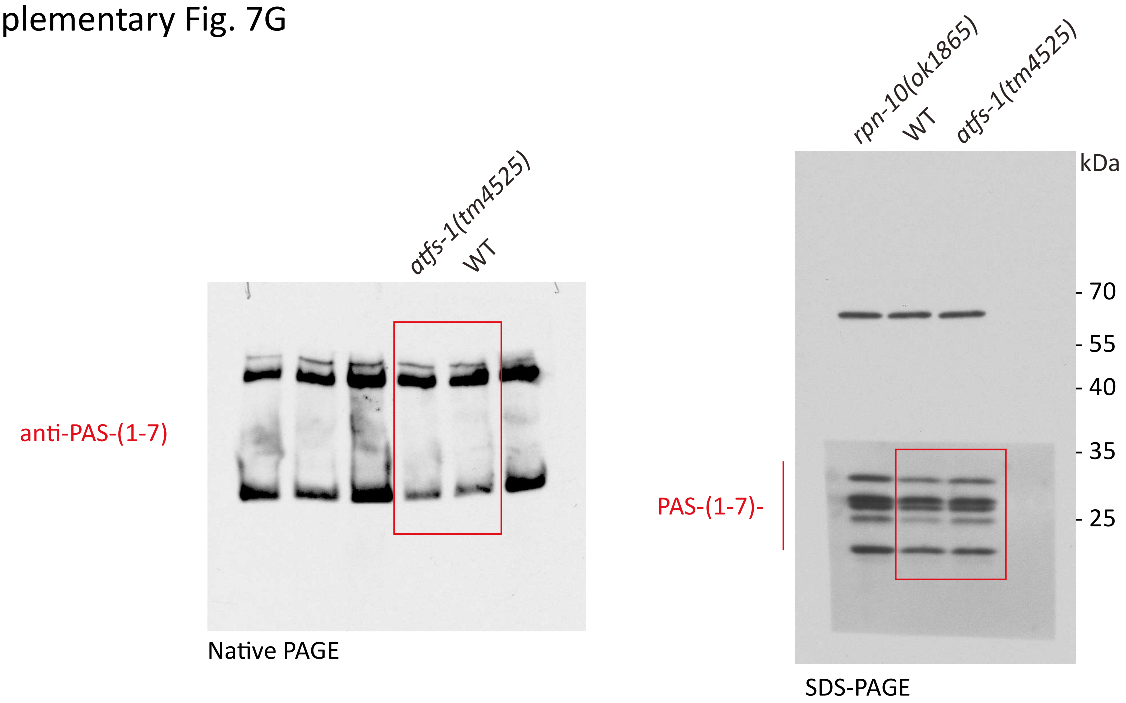

Supplementary Figure 7

Supplementary Fig. 7H

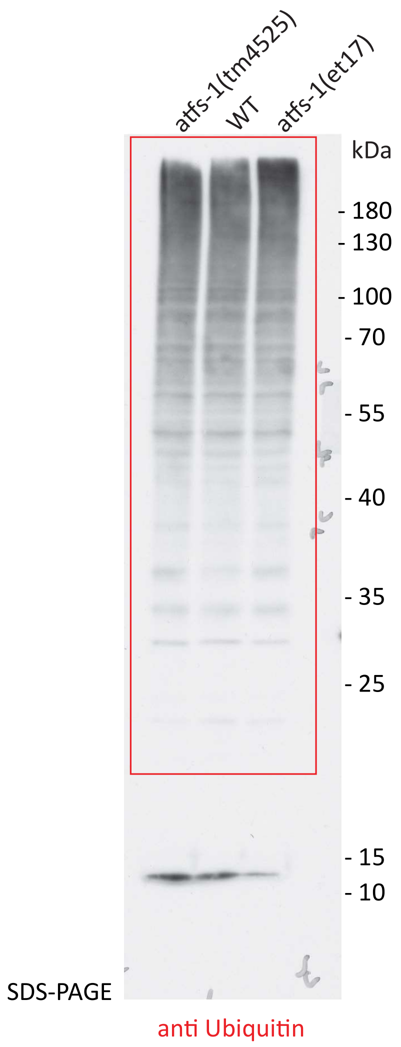

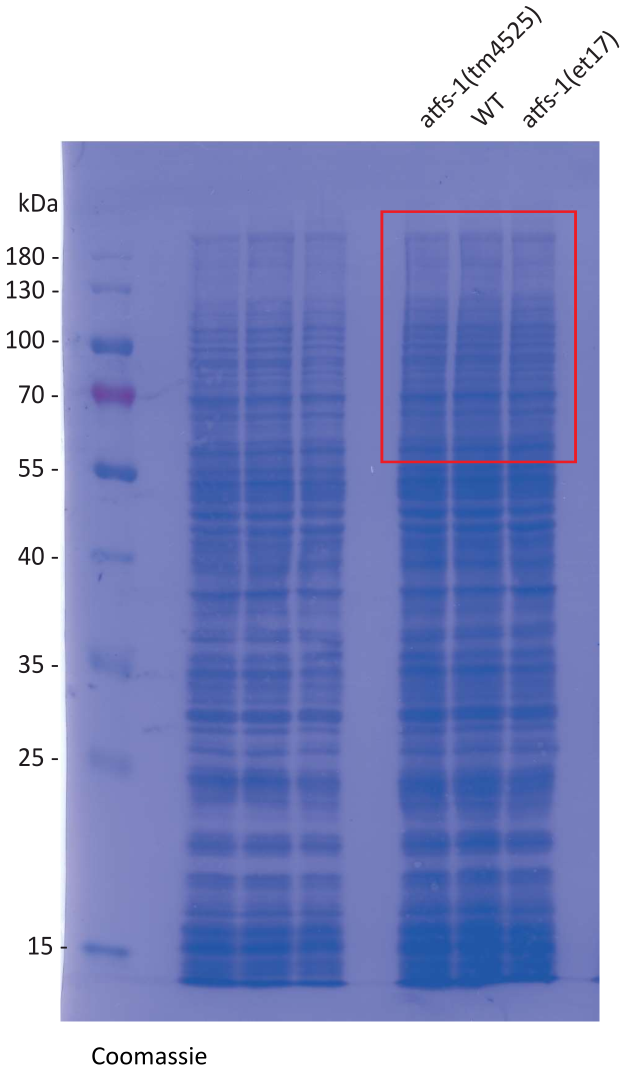

After protein transfer membranes were cut and their fragments were incubated with antibodies against indicated proteins.

Supplementary Figure 8

Supplementary Fig. 8A

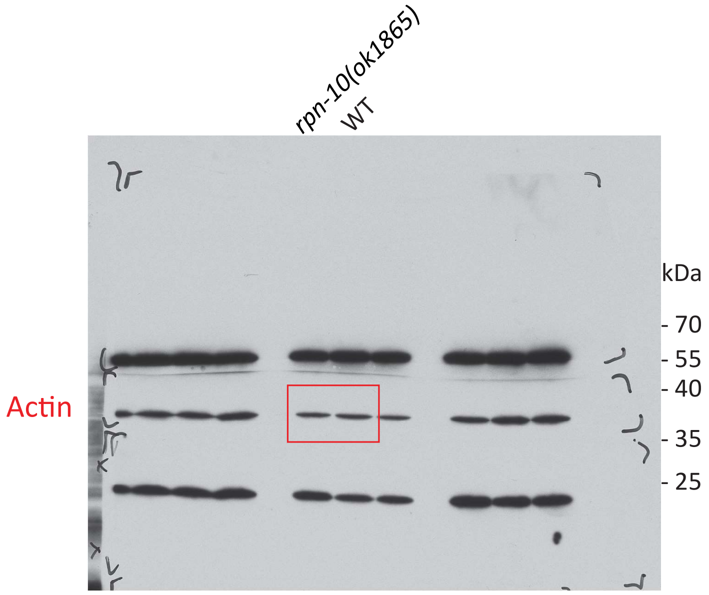

SDS-PAGE

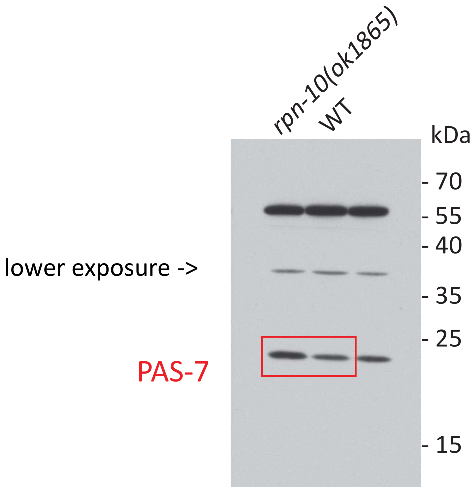

SDS-PAGE

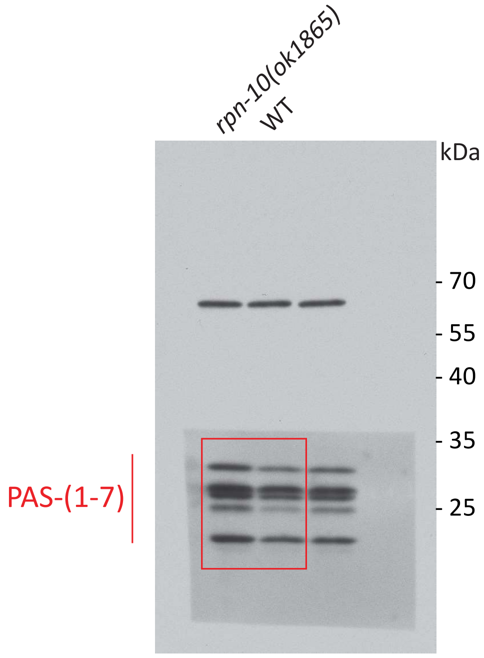

SDS-PAGE

Supplementary Fig. 8B

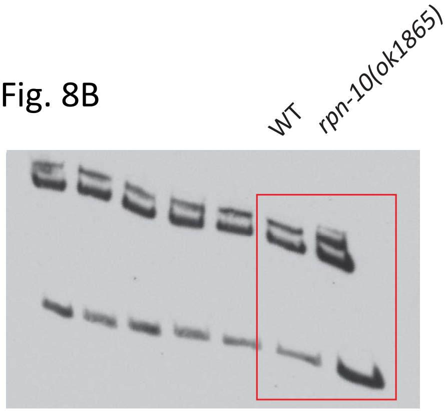

Native-PAGE anti-PAS-7

After protein transfer membranes were cut and their fragments were incubated with antibodies against indicated proteins.
